# Supplementary material for: “Getting pregnant during COVID-19 was a big risk because getting help from the clinic was not easy”: COVID-19 experiences of women and healthcare providers in Harare, Zimbabwe
Source: PLOS Glob Public Health. 2024 Jan 8;4(1):e0002317. doi: 10.1371/journal.pgph.0002317 (PMC10773929; doi:10.1371/journal.pgph.0002317)
Supplement: S2 Text — (DOCX) [file pgph.0002317.s002.docx]

**Study Title: Unintended consequences of the COVID-19 pandemic on Prevention of Mother-to-Child Transmission of HIV and Syphilis in Zimbabwe**

**Study PI Professor Mutsa Bwakura Dangarembizi**

**Phone + 0772601735**

**Topic Guide:** **Pregnant and lactating women**

**Introductory notes for facilitator:**

- We want to gather your experience and opinions, so that we can learn from you going forward
- Since this is the first time a lockdown has been implemented in Zimbabwe, we want to be able to learn from it so we can inform the communication strategy.

**Introductory questions**

1. To start, can you tell me a bit about what your yourself? (where you stay, age, marital status, household composition, employment status).

**Knowledge and perception about COVID- 19**

1. Can you please tell me what you have heard about COVID- 19/ Coronavirus?
2. What do you understand about it?
3. Can you please tell me how do you feel personally about the COVID- 19/ Coronavirus?
4. What measures or changes are you making in response to COVID- 19/ Coronavirus?
   1. Personal level
   2. Family or household level
5. From your perspective, how are healthcare workers perceiving the situation?

**Experiences of how COVID- 19 impacted their lives and PMTCT services**

*Inform the participant that you will be using a timeline approach. Show the participants a piece of bond paper with 3 marked events (pregnancy, HIV testing, Delivery and lactation).*

*Ask the participants to narrate their experiences from the time they discovered that they were pregnant to the current day. For each time period ask the following questions.*

1. In terms of living your everyday life, in what ways was your everyday life different from before the COVID- 19/ Coronavirus pandemic?
   1. Probe for daily activities, family and social life, income/finances
2. In terms of service and service delivery how have your experiences changed from before the COVID- 19/ Coronavirus pandemic?
3. How has the COVID- 19/ Coronavirus affected your mental health and general well-being, if at all?
   1. Probe on anxiety, depression and stress.
4. Can you please tell me about the PMTCT services you or your infant have been accessing from this facility?
   1. Probes - when did you start accessing the services, have you always used this facility
5. Could you please tell me what PMTCT services you or your infant received from the service provider(s) in this facility since the onset of COVID- 19/ Coronavirus pandemic?
   1. Probe for impact on scheduled clinic visits/ routine monitoring
6. What were your experiences of accessing services like since the emergency of the COVID- 19/ Coronavirus?
7. In the community you live how has the COVID- 19/ Coronavirus affected other people’s access to other health services?
8. Did you feel like you had all the information you required during the national lockdown?
   1. Probe information on where to go during pregnancy for your own health check-ups, for delivery and to access PMTCT services (including EID services).
   2. How to get to the clinic including the travel requirements
   3. What to do when you get to the clinic
9. Have you noticed any changes in clinic care since the onset COVID- 19/ Coronavirus pandemic including waiting time, quality of services?

**Social Issues at home**

1. Did you have any challenges at home regarding coming to the clinics during the national lockdown/
   1. Probe for disclosure issues, financial situation
2. Did you encounter any challenges with regards to taking your medication or giving your child medication during the national lockdown?
3. In your opinion, how do you think the following gender dynamics and factors affected women’s access and utilisation of PMTCT services in your family and community?
   1. Child care roles and responsibilities
   2. Access and control of resources
   3. Decision making powers at both household and community level
4. Do you have any other social concerns regarding your or your child’s health as a result of the COVID- 19/ Coronavirus pandemic?
   1. Did share your concerns with anyone else (probe for family members, HCWs).
5. The Zimbabwean government has implemented major social changes including social isolation, travel restrictions, closure of schools and boarders. What impact do you think they might have on you and other women in your community?
   1. Probe impact of roadblocks and having to disclose reason for travel in a bus with other people/ disclosure/ stigma
6. In terms of community relations and child care arrangements, have the community interactions changed from before the emergency of the COVID- 19/ Coronavirus pandemic?
7. How much of a concern is COVID- 19/ Coronavirus in this community?
8. How do people in your community feel about accessing health services from hospitals and polyclinics?
9. What measures or programmes are needed to mitigate the negative impact of COVID- 19/ Coronavirus pandemic within your community?

**The end**

**Study Title: Unintended consequences of the COVID-19 pandemic on Prevention of Mother-to-Child Transmission of HIV and Syphilis in Zimbabwe**

**Study PI Professor Mutsa Bwakura Dangarembizi**

**Phone + 0772601735**

**Topic Guide: Healthcare workers/Community Healthcare workers**

**Introductory notes for facilitator:**

- We want to gather your experience and opinions, so that we can learn from you going forward.
- Since this is the first time a lockdown has been implemented in Zimbabwe, we want to be able to learn from it so we can inform the communication strategy.

**Introductory questions**

1. To start, can you tell me a bit about what your yourself? (Age, marital status, qualification, and designation).
2. Can you please tell me how do you feel personally about the COVID- 19/ Coronavirus?
3. How has the COVID- 19/ Coronavirus affected your mental health and general well-being, if at all?
   1. Probe on anxiety, depression, and stress
4. Can you please tell me about your portfolio of work?
   1. Probe for current reassignment

**Healthcare context in Zimbabwe**

1. Can you please describe the healthcare context that you work in?
   1. Probe for any concerns about the healthcare context
2. What measures or changes are you making in response to COVID- 19/ Coronavirus?
   1. Personal level
   2. An organizational level
   3. How do you cope with these measures or changes? In your life or work
3. From your perspective, how are healthcare workers perceiving the situation?

**Experiences of how COVID- 19 impacted PMTCT services**

1. How has the COVID- 19/ Coronavirus impacted the delivery of PMTCT services?
   1. Probe for supply related bottlenecks in the whole PMTCT cascade
      1. for both testing commodities and the medicines
      2. Retention in care of both new and old patients
   2. Sample transportation
   3. Ability of HCWs to come to work.
   4. Fear of getting infected with the COVID- 19/ Coronavirus?
   5. Lack of PPE
2. How does the COVID- 19/ Coronavirus situation compare to your experiences of other disease outbreaks?
   1. Probe Cholera, typhoid
3. Describe the changes to your services provision as a response to the COVID- 19/ Coronavirus pandemic?
4. What challenges do you think mothers encountered in trying to access PMTCT services during the national lockdown?
5. Did you think your patients had all the information they required during the national lockdown?
   1. knew where to go for PMTCT services during the national lockdown?
   2. How to get to the clinic including the travel requirements
   3. How to handle themselves at the facilities
6. Have you noticed any changes in the number of patients seeking care since the onset COVID- 19/ Coronavirus pandemic?

**Social Issues at home**

1. In your opinion, how do you think the following factors affected women’s access and utilisation of PMTCT services?
   1. HIV status disclosure
   2. Childcare roles and responsibilities
   3. Access and control of resources
   4. Decision making powers at both household and community level.
2. The Zimbabwean government has implemented major social changes including social isolation, travel restrictions, closure of schools and boarders. What impact do you think they might have on women in your catchment area?
   1. Do you think these measures are feasible especially in your catchment area?
3. What measures or programmes are needed to mitigate the negative impact of COVID- 19/ Coronavirus pandemic within your community?
4. What do you think are some of the health impacts of the COVID- 19/ Coronavirus, including beyond the infection itself?
5. What do you think are the socio-economic impact of COVID- 19/ Coronavirus (short term or long term?
6. What do you recommend should be done as a national response to the COVID- 19/ Coronavirus pandemic?
7. What measures or programmes need to be put in place to help mitigate the negative impact of the COVID- 19/ Coronavirus pandemic with the workplaces and within communities

**The end**
