# Supplementary material for: “Getting pregnant during COVID-19 was a big risk because getting help from the clinic was not easy”: COVID-19 experiences of women and healthcare providers in Harare, Zimbabwe
Source: PLOS Glob Public Health. 2024 Jan 8;4(1):e0002317. doi: 10.1371/journal.pgph.0002317 (PMC10773929; doi:10.1371/journal.pgph.0002317)
Supplement: S1 Data — (ZIP) [file pgph.0002317.s003.zip › Data/Health Promoter/Health Promoter 1.docx]

Interviewee’s Gender: Female

Interviewee’s Age: Around 45

Interviewee’s Initials: HP 1

Length of Interview: 37:55

ZM: Alright, so the first question that I am going to ask you is can you tell me a bit about yourself how old are you, are you married you work here at the clinic as who?

RES: My name is XXX I am Muzvare XXX, and I'm around the age of 45 years I was born in XXX, I learned in XXX, I did my ordinary level and I passed 5 levels here I work as a childcare worker, I also work representing Mavambo as a community linkage. I also work as a behavior change facilitator at ZICHIRE, I am a volunteer I do many jobs in the community.

ZM: All right, I want you to tell me what you feel or what you think about the coronavirus, how do you see this disease?

RES: Ahh coronavirus disease is a scary disease that I have never seen ever since growing up to now, that a person stays with their mouth closed wearing a mask, a person stays with his/her nose closed and mouth that we never expected,

ZM; Hmm

RES: it’s a scary disease because people are being encouraged that we must always do social distancing, and not to greet each other with hands we were used to that in our tradition if you don’t greet a person with hands, you will be arrogant or spiteful but because of COVID we know that people are no longer greeting each other with hands.

ZM: Hmm

RES: It either you raise hand to each other like this (Demostrating) or cross-leggs or greeting each other with an elbow which is not what we grew up doing, also the issue coughing they said cough in your elbow so that you don’t spread the disease, this is a scary disease of course we knew about the flues were always there from back then

ZM: Hmm

RES: And when it started we heard that there was a flue outbreak in China we thought it would end there, later we saw it was now in Zimbabwe people were starting to die because they got sick, it frightened us a lot and it affected many things in our country even in or community, the way of living to the extent that other were writing on their gate that no visitors

ZM: Hmm

RES: We stopped doing home visits before the government announced the lockdown because people did not want us to come into their homes. We would come across notices at the gates or doors saying, ‘do not enter we do not want visitors here’. Many people in the community are still afraid of the virus considering how it is spread so they are not entertaining any visitors.

ZM: All right, did it affect or trouble you mentally or have depression or stress?

RES: Yeah it troubled me as a person who works with the community our work walking round, but we were limited to walk even at the clinics we were nolonger going we were working on phones but at that home lets say you have been visited you will start to think where this person is coming from how did she travel what if she has come with it and infected us. So, what is going to happen to my children what am I going to start quarantine?

ZM: Hmm

RES: Sometimes where we stay accommodation in the location is a problem maybe you use one room you are now thinking where I am going to be quarantined, so am I going to be removed from here what about my children who will I leave them with, all that I get to a point where I was troubled mentally, I was afraid for sure.

ZM: Hmm

RES: If I get infected. when I had flue haa I was stressed thinking that it was COVID, so I started thinking about that If I was going to quarantine who would I leave my kids with, how are they going to survive when I was there if they say 2 weeks there, it scared me a lot, and it touched me I don’t want to lie.

ZM: How old are your children you were fearing who you leave them with?

RES: There is the eldest who has 17 years then there’s the other one is 10 years then the youngest the last born has 3 years that’s the one I was fearing for the most who has 3 years.

ZM: All right, can you please explain to me we talked in short when we talked about the work at this clinic and community, what are the jobs that you do as a community linkage or as a volunteer?

RES: Okay In the community we work with…we teach people like at ZICHIRE there is what is called SRH (Sexual Reproductive Health) we teach them that if they are having sex what they must do, they must protect their health they must not do unprotected sex because they might get infected with other disease other diseases like HIV/AIDS, STIs so we will be teaching that they must have protected sex they must use….there are male condoms there are female condoms all those they can help especially to young mothers they can wear female condoms.

ZM: Hmm

RES: They help a lot so that they don’t get infected disease because we know that most men, refuse to use condoms so you as a woman just wear your female condom.

ZM: Hmm

RES: We will be telling then that if you wear it an hour or 2 hours before, it takes the shape of your body it will be warm to the extent that if a man enters, he will not feel that theres something

ZM: Hmm

RES: Then on linkage, we will be teaching again the issue that we link with the community and clinic we follow those who are positive like the mothers who are positive wanting that their children who were born positive are aware of their status the ones one that they gave birth to

ZM: Hmm

RES: Because someone might be positive taking medication and has a baby that was born positive but didn’t get him/her to be tested, so we go and we refer them to come to the clinic to get their biological sons and daughter tested not the ones they are taking care of but theirs the ones then born

ZM: Hmm

RES: We give them referral slips that go and get tested its helping for sure because some children are being caught positive when they come to the clinic some wil be negative, so some will say you have helped me because I was relaxed not knowing that it my be possible that my baby is also positive

ZM: Alright, what about looking at PMTCT what is the work that you do?

RES: Okay we will be teaching the women that if they are pregnant they must quickly get tested for the sake of the babay that if the mother is positive they will be given medication to prevent the baby from contracting HIV because some women if the get pregnant they don’t go and register some will relax until they give birth,

ZM: Hmmm

RES: So we teach them the advantages of that if you get registered early then you enter into ART you will be givem medcation that you take and will prevent the baby that when you are delivering the baby doesn’t contract the disease

ZM: Hmm

RES: We tell then even on the day of delivering when they are due when they are feelin labour there is a pill that they are given that when they are delivering the baby doesn’t contract HIV, we teach them again that if they have deilivered that baby must stay for 6 months not giving her milk….ah giving her milk not giving him/her anything even water, or what or porridge what is called exclusive breastfeeding

ZM: Hmm

RES: Because if they give the that child food it might scatch his/her mouth when he or she drinks because milk has more virus, when the baby is drinking milk he/she might contract the virus from the mother

ZM: Hmm

RES: So we say its either if youre thinking of breast feeding makesure its 6 months not giving the baby water,not giving the baby porridge or what, giving the baby milk only if you have thought of not breastfeeding they dont breastfeed at all, then you find good food for that baby that he/she will be eating

ZM: Alright, looking at the issue of getting treatment in Zimbabwe without looking at the HIV pandemic, just looking at access of treatment services in Zimbabwe looking at maybe what was happening long back, can you say there something that has changed what can you say about our health context right now, looking at treatment only without anything to do with preganat mothers without anything to do anything with be it children or be it adults, looking at what was happening back them soon after independence and what was happening in between and what is happening now ,what can you say our health context is standing like right now

RES: Right now looking at treatment long back it was better it didn’t want too much money so now yes they are treating there that many things that have changed but the issue that is troubling is about money that if you don’t have money if you don’t have cash you can die at home

ZM: Hmm

RES: Because you can arrive and told that to stamp your card they need 5 dollars maybe you don’t have that 5 dollars or where to find it for you to stamp the clinic card ,so you will go back home , sometimes you are supposed to go to private, they will be calling for a lot of money you, cant find anything to do but long back you were treated for free without paying any money the card was stamped for free then you get the services then do what then go home

ZM: Hmmm

RES: But not they are treating but the issue of money is the problem and also the issue of our contry’s economy its not standing well so its not everyone who affords to get the money to pay for them to get treated, to get treated some people…..last time I saw a man who died at the gate coming when we were coming to the clinic

HM: Hmm

RES: He was lying down then we said this man who is lying down is everything okay, after we have seated we were working outside we saw the grandfathers entering with him in a stretcher bed covered with a white cloth the whole body then we saw that the person ahd what had died

ZM: Hmm

RES: He died outside the gate because of the issue of opening the clinic late that’s another problem

ZM: Alright, looking at that issue you talked about the isuue of shortage of money, late opening of the clinic what about looking at how people were handled even information that was given to people when they come to get treatment nad the time they took is there anything that has changed looking also at how people are being treated in Zimbabwe comparing with what used to happen back them

RES: What I see personally is that what was happening back then was good now maybe its because sometimes the nurses will be saying the money that I am getting is little the way you are treated sometimes you see that ahh it’s a bit different long back for sure if you see a nurse or a dictor you would feel that yeah I now going to get treated but now I see that there is a differencethat is there

ZM: What is happening that is causing that difference?

RES: I dont know but I hear peole saying that I work according to the money that I am getting because looking at back then they getting paid with money that buys, like now a person will be saying I work according to my pay I think that is what is affecting people not to do their job wholeheartedly if I am looking at it that’s how I see it

ZM: Alright looking at the issues of there were many measures that were done when COVID happen you personally standing on your own what measures did you take in trying to reduce contracting Coronavirus or trying not to infect other people is there anything that you did or that you changed on your way of living you as a person alone at your home

RES: I can say what I changed on my way of living is that I was staying at home, at our work most of the time they were saying lets work on phones we were told to protect ourselves, use phones reporting system was done using phones, i would be found home the everyday, we were also using sanitisers

ZM: Hmm

RES: We were given sanitisers at work they are the ones we were using that if a child goes out if he/she goes to the toilet when he/she comes back sanitise,was monitoring my children that they should not walk going out side because you might take it outside then bring it home, I was telling them that people should play in gate, thats what I changed and fearing not to infect others and also for me not to get infected by the disease

ZM: Alright, what about looking at the organisations that you work with you talked bout ZICHIRE, you talked about her at the clinic what changed here trying to protect the workers and trying to protect the patients

RES: Here I can what change is that people were given PPE to use even when entering the gate, enetering the get you are taken temperature at the gate nad sanitized then you anwer what you are going to to, at the gate you will be sceened before you enter that what do you want to do inside at the clinic of which people will be in a que outside getting in one by one ,they are nolonger getting in like people crowd in the clinic like they used to do long back there is a difference

ZM: Hmm

RES: A person is taken temperature first the get inside then sanitized but don’t just enter all at once you stay out side the gate

ZM: Alright looking at that was there any lowering of number of people who were helped per day or who were seen or the number remained the same but people were now entering in few numbers , but if they were serving 50 before COVID came now are they still serving 50 people?

RES: Yeah that right, I cant say the number of people who were being served has decreased or increased I see like its still the same but they are just limiting the way you get inside,everyone who had come to the clinic are being served well

ZM: Hmmm

RES: So they are nolonger doing what they were doing that you would get in going straight to where you want to go, if you want to go to OI you would get in if you want to come here to out patient to get treatment you would come but now they first screen you at the gate but the number of people who are being treaeted is till that same there is no one who is going back without being treated

ZM: Okay looking at you that you said you are nolonger travelling we going back to you, you said you implemented measure you are nolonger walking, youre sanitizing your children can you say thee is anything that these measures changed or affected you in your life

RES: So far for me there is nothing, there nothing that was affected because my job I was doing over the phone, but if we were not allowed to work on over the phone I was going to be affected a lot on work it was going to affect me, so our boses were afraid and by following the law they sad you will stay at home working with phones you will report on the phone,

ZM: Hmm

RES: They would put what they wanted on the phone then we do the, clients we were sending them messages on the phone then reply on the phone again then I report on the phone so I wasn’t affected

ZM: Alright what about looking at the clinic now people are being screened there can you say there is a change that it has brought in treatment of people or that there is anything that has been affected or did it affect be it people who get treatment or the clinic

RES: It affected peole who hgt treatment like especially at OI where we work at sometimes doing linkage ahh people were complaining because they were saying they were being called at the gate that those who want pills go there, those who want to be taken viral load go there some people they don’t want with their statuses they don’t want to be known, like they take the books of the patients there so some people will get his/her book taken by grabndfather then disappears they will then call so and so……

HM: hmm

RES: They will come back and calling those cards at the gate so people will be mixed those who didn’t come to OI and those who came, so for a person to be called his/her name some they don’t want some disappear for sure they might come in the afternoon around 2/3 he/she will pretend like he/she is going then go to take their pills

HM: Hmmm

RES: And people are not liking it to tell the truth especially those on ART those of OI they don’t want because they dont want their statuses to be known with people of their area

ZM: Alright looking at you as a person who work with health and nurses can you the problem of COVID or the COVID disease how are people taking it

RES: COVID disease in the community or here?

ZM: Yes in the community here at the clinic we want how the people are taking it people of health not people, the nursese how are they perceiving it, how are they seeing like you health workers you work with people how are you accepting it how are you seeing it?

RES: We have accepted it as a disease that has came there is nothing we can do, what we can do the most is to protect ourselves if you look at our nurses they know that COVID is there, if you look at them they are wearing enough to protect them from up the head to the toe masks they are wearing, they are wearing those ontop to prevent so that they don’t contract the disease, they are afraid they know that COVID is there they are supposed to protect thenselves very much

ZM: Okay coming to the issue of that looking at PMTCT services can you say corona has anything that it affected on the delivery of PMTCT services

RES: Yeah has affected

ZM: Hmm can you explain to me what it has affected

RES: Okay like long back when there was no COVID if a person got pregnant if we teach that come and register whilst the pregnant is still small even at 2 months people would go to register

ZM: Hmm

RES: But now a person would go sometimes and returned saying there are no cards,we are not registering here then they go back home until they deliver their pregnancy so if you had HIV its obvious that the baby you will give birth to will be positive

ZM: Hmm

RES: Of which the baby was supposed been done PMTCT and born without getting infected with HIV because PMTCT is making children come out negative so that they do not contract but because of COVID truly they gave birth without registering preganancies and they were giving birth at homes without even protection the midwives would not have even the gloves or what

ZM: Hmm

RES: Where someone who had given birth that’s where you will be going there they will say lie on the same plastic there are no gloves or what of which the chances of contracting the virus will be high

ZM: Alright what about looking at the issue of that people can be able to be taken viral, can be able to be taken tests to see if you are HIV positive when you do to register your pregnancy was that affected

RES: That was affected again because the nurses were not coming like I think they were giving each other duties that you youre the one who goes to work this day you youre the one who goes to work that day

ZM: Hmmm

RES: So the nurses were few so for people like testing it wasn’t moving even VL viral load people were not taken, I think I don’t that the kits were not there or they just didn’t want to work the grandmothers I don’t know but testing they were saying we don’t have testkits

ZM: Hmmm

RES: You see those who was on emergency emergency was the one who was allowed to be tested for HIV not just coming that I came to get tested they were told that go back because of COVID

ZM: Alright, what about looking at transportation of samples those few who has been tested were the samples moving well to go the lab and also for the results to come back

RES: Ahh I don’t see like they were moving well because we have children some we work with, the clients that we help to do adherence support who will be positive they will always be told that they are pending upto now they are not yet out those results they said they are pending meaning that maybe where they are taken its also on going well, they didn’t go well because of COVID

ZM: Alright, what about looking at the nurses here at the clinic were they able travel well coming to work or in the national lockdown the one that happens at the beginning?

RES: Ahh travelling they were not able to travel of which they were said they can travel bcauase they were the ones who were allowed to travel but you would hear a person saying that they failed to come to work because they didn’t find transport

ZM: Hmm

RES: I failed to come to work because I didn’t find transport meaning that it was hard, transport was a problem, for sure when they said total lockdown it was not easy to travel, people would stay wherever they were, so you would see that the some nurses that stays near its only a few who were able to walk on foot to come to work those were able to come

ZM: But those from afar were suffering?

RES: Haa those from afar the were failing to come so they were not coming

ZM: What about looking at you and nurses on the issue on fear, afraid of contracting coronavirus did that make you stay at homes and stop doing your job like everyday like the way you are supposed to be doing

RES: Haa the truth is we were scared not even playing because you will be knowing that thats were everyone comes, you can get infected there was someone who was infected and was told to quarantine was told to stay home, he/she was working here doing voluntary work as well

ZM: Hmm

RES: So that time the fear increased to the extent that coming here was a problem that ahh for me to go to the facility today although we were not needed everyday but here and there you would see that fear was there even the grandmothers the nurses they were afraid of COVID there is no one who was not caught with fear of COVID

ZM Can you say the accsess of things to use like PPE affected the way nurses work?

RES: When they had not yet come?

ZM: Hmmm

RES: Yes affected because a person would be fearing that so if I touch this what if I touch a person without protection maybe I can get infected by the disease, you would see that the way they work before they found PPE and now PPE is there it’s a bit different, because now a person is now working they are now working the grandmother freely knowing that they have protected themselves they are wearing protectives

ZM: Alright we want to hear your thoughts you have seen cholera that happened in Zimbabwe, you have seen zimbabawe having a problem of typhoid comparing what was happening during the days of cholera and days of typhoid and now can you say corona is different with your experinces that you had before, this disease comparing it with other outbreaks that had happened before what can you say corona is like

RES: I can say that corona is too much of course typhoid was killing but ah corona is too much on that the spreading is that everywhere typoid would have an area that it infected lets say if its Old Mabvuku you would know that you don’t play in Old Mabvuku there is typhoid if you are from Tafara then stay in Tafara

ZM: Hmm

RES: But COVID is said everywhere if you go somewhere you can get infected so ihhh corona is too scary I don’t want to lie

ZM: What about looking at the way the clinics were working, the way nurses were working looking at the time of cholera and now is there a difference?

RES: Ahh during the time of cholera we workers didn’t need PPE that much, time of cholera the nurses were working for sure, they would work putting people on drips those who were being transferred were transferred there and there they were working,its dofferent from COVID the way it came that if you get in contact with a person if you do this you have been infected with COVID it came with a lot of fear

ZM: Alright we have talked about the manty changes that happened we want to see that the mothers when they were at home because of corona those mothers who were preganant, those who had just given birth who are supoosed to be accessing PMTCT services what can you say are the problems that they has encountered during lockdown pertaining being able to access the services the mothers

RES: The mother who were pregnant and those who had just given birth the problems they encountered are many,because after giving birth you would want to go to the clinic with the baby or to be given services that he/she is supposed to be given

ZM: Hmm

RES: For you to travel and go with the baby you woukld be scared that sometimes I might get infected with that disease, then you stay at home sometimes when you go to the cinic there was a problem that is happening that there are no cards there is no what scale cards, we don’t have enough things because things can not be transported beause of COVID

ZM: Hmm

RES: So they were affected too much some ended up staying with the child now there are children who doesn’t have scale cards even for them to get baby records written they were born at homes they didn’t access BCG the baby was stayed with at home because people were not travelling

ZM: Alright what about looking at the mothers or the patients can you say they enough information during lockdown about PMTCT services where to get them and how I can travel

RES: They had the information because in the community there health workers that are there we help each other, We walk around following these preganant mothers teaching them they will be knowing

ZM: Hmmm

RES: But I see that issue of fear that sometimes I want to go im pregnant then contract COVID at the clinic, because there were some discussion that people were doing that at the clinic thats were you can get it, they were saying sometimes you can leave home fine but when you go to the clinic that were you contract COVID

ZM: Hmm

RES: You would see that a person is sick but will be saying that I don’t go to the clinic If i can get to the clinic they will say that I have COVID,the same with the mothers who were preganant they knew that I can get PMTCT at the clinic but they were afriaid of going they were afraid of getting infected with COVID

ZM: Alright, Can you say they had knowledge on how they could to travel to the clinic because during national loockdown everyone was not allowed to travel except essential services, the mothers were they aware that if you want PMTCT services how do they travel were they on essential or they are not on essintial how to they go about it pertaining travelling

RES: We encouraged them that they should not travel far its better to go to the clinic that is near you to seek the services there, go to the nearest clinic or where you are able to walk by foot if you stay in Tafara go to Tafara

ZM: Hmm

RES: If youre from Mabvuku walk and go to Mabvuku clinic you will get services not travelling or boardering a bus or wanting to go to town or Prirenyatwa we were telling then that its not encouraged

ZM: What about looking at the issue that when the arrive at the clinic did they know how they are supposed to do, how are they supposed to handle themselves when they arrive at the clinic

RES: We were telling them, yes we were telling them that if you arrive there you should say that you want either to register pregnancy or to be given medication my things are like this, I want to able to protect my child who is in my stomach he nurses will be knowing that if a person ios like this we are supposed to treat her like this

ZM: Hmm

RES: They knew but I see that the issue of fearing COVID was the one affecting fearing that if a person is infected, I heard someone saying if I get infected with COVID whilst I am preganat will I give birth to a child who has COVID

ZM: Hmm

RES: Then we said that’s not it COVID is not infected new born babies its infecting adults you must go but a person will be scared that I may leave there infected, im already pregnant if I contract that disease what is going to happen after I have infected by covd whilst I am pregnant

ZM: Hmm

RES: So people were preffering that its beter to stay home

ZM:What about looking at the number of people who were coming to the clinic to get treatment can you say it decreased or it increased looking at the time of COVID during the first lockdown that happened,can you there anything that changed on the number of people who were going to to the clinic

RES: Ah during the first lockdown people were few, people were afraid of traveling for sure, and people were not going…a person would say if its flue I will treat it at home if its headache a person would look for pills saying I don’t even want to go to the clinic, people were staying at homes but on the second lockdown people were doing like thay now had knowledge they were not increasing at the clinic going to seek the services

ZM: Alright looking at the issues that were happening at homes the social issues do you think is there anything that affected the mothers because they were now staying at home with the fathers looking at the issue iof status disclosure

RES: Yeah that was difficult for sure because you find that the husband will be working up and go to work everyday sometimes these men were taking medication at work some their pills stays in the car that the wife doesn’t know that the father on ART , but now because people were staying together it then came out

ZM: Hmm

RES: Some ended up fighting or eneded up divorcing that the father had not told me that hes positive sometimes the wife wil be negative the husband didn’t disclose his status to his wife teliing her his status, because lockdown said you shoud stay at home you would end up finding out that that’s what is happening here so haa it was difficult for the couples it was a bit troblesome

ZM: What about the issue of child care, the roles that all the children are there no one is going to school is there anyhthing that changed or the challenges that the mothers had on the issue of that has childcare increased peole who would spent their day at school they were now spending thier day at home

RES: Yeah sure it was a problem because if the children are there noise and fightings, you would be knowing that children wakes up and go to school it will be quite at home.So you would see that for sure in these houses…plus food if a child is going to school they eat in the morning then they spent the whole day at school ten comes back in the evening

ZM; Hmmm

RES: At that house they will be wanting to eat from morning, afternoon and evening youre looking at each other so that when you would see that the father and the mother will end up fighting that children are crying with hunger the husband will be saying what do you want me to do, the husband will say I don’t have the wife will say I don’t have,because you are just looking at each other sometimes lets say the wife was a vendor lockdown said you must not walk stay at home you would see that it was causing GBV at home

ZM: What about looking at the issue of who makes decesions at home the mother is there the father is there was that affected because of lockdown?

RES: Yes

ZM: Uhm looking at decision making at home

RES: Decision making at home its obvious that the father will be saying im the one who does decision making but sometimes when the father is not there the mother will be the one who will be making decisions

ZM: Hmm

RES: You would find that when the father is there its difficult like my children likes to watch wresting, the youngest likes to watch cartoons, the elder the one in the middle likes to watch wrestling their sister likes to watch Indians, I like to watch African movies you see, so you cant find who will touch the remote to put the station they want it will be troubling, in other houses the father will be saying that he wants to watch soccer it will end up with noise this one is saying put this channel the other is saying put this channel

ZM: Hmm

RES: Or the father can say today in this house we are eating with okra then the other children will say I cant eat sadza with okra I want meat but the father had passed the decision that no we are eating with okra,

ZM: Hmm

RES: So its the father most of the time who makes decisions if the father touches the remote and says he is putting soccer whether you like soccer or not you will watch soccer because thats what has been put that’s what there ,so its the father who makes decisions but it will cause problems at homes

ZM: Looking at your community where you stay can you say the measures that were iimplemented in trying reduce the spread of….like people stay at homes, don’t move around, the closure of schools and that people must social distance, they must do social isolation they must do quarantine do they work in your community where yoou stay these measures looking at them can you they can be done not that they work but is it practical, can it be done in your community?

RES: We can say that when the lockdown started the first one I don’t want to lie that time everyone was afraid and they followed the masks were worn, social distance was done but when we had the second lockdown I don’t know what has entered into people

ZM: Hmm

RES: Ahh at the shops you would see peole crowded there no one is wearing masks even social distance. We even teach about social distance at boreholes we suffered because if you would pass through that you would say ahh see you didn’t do social distancing they pretend to listen that moment you leave then go back to crowding

ZM: Hmm

RES: Same same at the shops you would see people seated drinking beer but we are In lockdown but for people to follow I dnt want to lie in location hmm they never followed they only followed on the first lockdown that when they had alot of fear but this time people were doing whatever they want even mask…there is someone who came doing this other work shop that boy said to me that is there lockdown in Mabvuku Tafara is there COVID I say why then he said there is even 1 poerson I saw who was wearing a mask, people are walking without meaning that people all that fear has ended I don’t know why

ZM: Whats making people not to wear masks walking doing as if this diseases not there?

RES: Not fearing plus I see that people wakes up if they had seen that there is a person who had died because of COVID at their next door,if they are hearing that a person has died is from Mbare or Glen view or where they are not taking it serious to know that COVID is real and its there and it kills they are not taking it serious

ZM; Hmm

RES: They will panic after It has happened at their next door or in their house that it has infected a person and died but right now if you talk about COVID a person wll tell you that ahh leave us ,they don’t what to follow they are not afraid I don’t know why

ZM: Alright looking again going back to the issue of social distancing, that of isolation, quarantine do you think that it’s feasible looking at how people live in your community

RES: That it is….

ZM: Can it happen, is it something that can happen is it feasible that you can say that ah people are able to this and people this they cant looking at the houses that peole stay is it a thing that we talk about and its happening or some of them are dreams it can’t happen

RES: The issue of quarantine to me for it happen they would have come and taken the person like what they were doing when COVID started that a person was taken to quarantine somewhere, that a person must be quarantined at home it cant, there is a father who got sick at my next door and after fallen sick that father it is said that he was feeling shortness in breathing and difficulty in breathing he was doing all the signs of COVID

ZM: Hmmm

RES: He stays in the car sometimes, they use 2 rooms sometimes he would spend the day in the house, that’s the house were children are entering, that’s he housee were everyone is entering so I saw that quarantine doesnt work in locations it doesn’t work

ZM: Hmmm

RES: Because there are no houses for him to stay alone and use all things alone even the toilet will be one at home, that is used by everyone so him to get his own toilet to use there is nothing so people would be sharing so you will see that sometimes its God who is keeping us fou sure that father at the next door I was afraid

ZM: Hmm

RES: You know he stay close close that I was always saying that igh its God who protect the children from that house were coming a lot at our house,if we chase them that you go back and play at your house they would keep on coming so you will that you will end up troubling people they will be saying that they are chasing our kids, maybe they are thinking that that have COVID ahhh its hard some of the things you will just say only God is our protector

ZM: So what can we say that the government must do , what are the measures we have talked about the problems that were encountered by mothers because of not getting services ,fear of coming to the clinic, the nurses were not coming what can the goverment do lets say another disease happens like this in future, we want to say that no we learned from corona we are supposed to do this and that so that the services don’t get disrupted, so that they keep on working well what is supposed to be done what are the measures or programs that are supposed to be donm so that the services of PMTCT can never affected

RES: I think that if they can introduce the hospitals that stands for those who are pregnant only they should be many for the sake of those who are pregnant they should not travel long distances going to get treatment, then the government workers if its possible they must be able to be given cars to use, that they can be given fuel with the government it will be easier that you get into your car then you go to work there is no fear that you say ah if I get a lift maybe I will get infected

ZM: Hmm

RES: But do you know that you get in your car you are going to work and then also PPEs that are enough to use I think this can help

ZM: Looking at this disease and saying people should stay at homes can you say this disease has affected anything on social economic, the impacts that it did be it on families be it on country, is there anything that it affected looking at the issue of wealth, getting things, getting money did this disease affected?

RES: Yeah it affected because we see that our economy was already bad then COVID came it became worse because those were doing bussines they were now seated to do this there was no money that was circulating or do what things were just standing on place so on that COVID affected a lot in the country

ZM: I think you have answered all the questions that I had, I dont know if you have some that you would want to recommend or that you that you want to ask from me that how can we go about whar can youwe do pertaining this disease

RES: Okay I wanted to say that COVID we are hearing what is being said I don’t know if it’s true that there is a third wave that is rising and its coming with power of which people are being injected with injection to prevent COVID, so COVID doesn’t stop making waves, that 1^st^ wave 2^nd^ wave, meaning it’s a disease that is going on and on meaning it will reach to 4^th^ wave or 5^th^ wave

ZM: So this disease keps on changing and mutating like flue there are variance many flue variance even though it’s not deadly but I think they are many variants because it’s a virus, what happens with the virus is that it changes with time and air it doenst remain as it was at the beginning

RES: Okay

ZM: At fisrt we had the first variant that was known then we had a second variant now it is said there is the other one that is called South African variant there is another one called UK variant, so the variants are now different so we don’t know because it doesn’t stop it keeps on changing so we might get to variant 20 maybe it might be scary, right now its said theres another one that is more infectious than the first one and the time its sickening is now short so it keeps on changing

RES: So as it stands it means that we can live with COVID even for 10 years or, will this disease end or it will keep having these variants coming others coming others

ZM: The problem at this time is that we don’t have 1 because it’s a virus we don’t have the medication to cure we are hoping that what is going to help us is the issue of vaccine

RES: Alright

ZM: But we do not have the knowledge yet that we are going to stay with it until when, maybe depends on whether the medications comes or that maybe we will be injected a vaccine that everyone who has been vaccinated will never contract maybe that’s what will make it decrease or when God intervened, so the truth is that we don’t know where we will reach with it, we can do 20 years or do 15 years or we can live with it like what we are doing to HIV that we already know that’s what is there

RES: Okay eish

ZM: Yes there no one who knows if its going to to end, is it going to pass, is it staying, what is it doing there is no one who has that truth but peoples hope is that if many people gets vaccinated it means they has ben prevented from getting infected thats how it is going to be reduced, maybe it will just end but that knowledge that is it going to end no one has it at this moment

RES: Okay thank you

ZM: Thank you I am going to ask you to…
