# Supplementary material for: “Getting pregnant during COVID-19 was a big risk because getting help from the clinic was not easy”: COVID-19 experiences of women and healthcare providers in Harare, Zimbabwe
Source: PLOS Glob Public Health. 2024 Jan 8;4(1):e0002317. doi: 10.1371/journal.pgph.0002317 (PMC10773929; doi:10.1371/journal.pgph.0002317)
Supplement: S1 Data — (ZIP) [file pgph.0002317.s003.zip › Data/Health Promoter/Health Promoter 2.docx]

Interviewee’s Gender: Female

Interviewee’s Initials: HP 2

Name of the Transcriber: Rumbidzai Chisora

Name of the Translator: Rumbidzai Chisora

Length of Interview: 20:50

ZM: My first question can you explain a bit about yourself, are you married, who do you stay with, what’s your level of education, and your designation at the clinic?

RES: My name is XXX I am married, and I have 2 children 2 girls in grade 5 and form 1. I stay with my husband and my highest level of education is form 4, at the clinic, I work as a community facility linkage working with those who are on ART.

ZM: All right, when say community facility linkage can you explain to me what your job comprises of what are the things that you are expected to be doing?

RES: Our job looks like we work with children who are on ART mothers who are pregnant who are on ART and parents who are on ART.

ZM: What exactly do you do when you’re working with the mothers who are pregnant mothers?

RES: Pregnant mothers we will be encouraging them to do what is called PMTCT, to register early then they know their status they get on ART if they test positive.

ZM: Hmmm

RES: We also do what is called adherence support calling them asking did you go for a review, did you take your medication, if it is working, and if they are encountering any problems.

ZM: All right

RES: That is the same with children

ZM: We want to talk about the issue of COVID-19 how do you feel personally when we are talking about COVID-19? Before we look at the job that you do, if you hear about COVID-19 how do you feel?

RES: I am afraid of it

ZM: Yes

RES: I am afraid I can only say I am afraid of it

ZM: What makes you fear it?

RES: What makes me have this fear is I work with the public so I don’t know where I will get infected that’s why I am afraid.

ZM: When you first heard about this disease of corona can you say it affected your mental health, have you ever had anxiety or depression? Did it affect anything on you personally looking at your health and the way you live?

RES: It affected me a lot because I am a known diabetic and hypertensive patient, and I was afraid and anxious that if I go to work what if I get infected with the disease… some of my colleagues got infected and tested positive though with minor symptoms so I was worried about doing home visits that maybe I will get the infection from there, so it was difficult for me to carry out my usual duties

ZM: Hmm

RES: We were also afraid that our jobs they might end because we said that now we are not going to work so it…. Affected me a lot.

ZM: All right looking at how we are doing treatments in Zimbabwe at the clinics, can you say there is a difference that is there on what was happening from maybe independence till in-between and right now? Is there a difference that you are seeing in our health care system as a country or as Zimbabwe? The way people were being treated in the clinics before COVID-19 came and looking at the health context in Zimbabwe what are you seeing or what can you say about the way people are being treated?

RES: Hmm the way people are being treated depends on the clinic's treatment well some clinics ahh……...

ZM: What do they do?

RES: A person pays money for a consultation and a card stamps the card, but you do not get any medication because there will be no medication at the facility. You have paid the money, but you are told to go and buy medication at the pharmacy if you are lucky, you will be consulted and given medication.

ZM: All right what about looking at how people are treated, how they were treated long back and now are there any changes in the health system?

RES: Now we can say that the way people were treated long back they were right especially to those who were on ART, for those who are on ART they are saying we take books before 8 or at 8 o’clock.

ZM: Hmm

RES: If you come late with your book, they will no longer be taking the cards but long back you would go even at 2 o’clock and they would give you your medication but now they are no longer doing that.

ZM: What is happening that is making them say we are only taking at 2 …..They are taking cards at 8?

RES: They take in the morning

ZM: Yes

RES: We don’t know why they are doing that because they say 8 o’clock we are collecting books if you arrive around 9 or 10 they will say go back and come tomorrow be on time

ZM: Okay looking at from the onset of this disease of corona are there any changes that you have made you personally in your life in trying to protect so that you don’t get infected with HIV, is there anything that you started doing that you were not doing, is there anything that you stopped doing that you were before, you looking at yourself personally from the onset of corona virus is there a change that you saw that have changed or that you changed

RES: Me what I have changed from the onset of corona is that the clothes that I had come with from the clinic I arrive and remove then I wear another clothes if there’s is water I wash them, I am no longer wearing clothes the whole day that I have come with from the clinic no If I arrive I remove I’m taking other clothes and wear

ZM: Is there anything that you have changed?

RES: And at home?

ZM: Yes at your home on your living you personally

RES: Alright, at home I changed that if you have come back from playing wash your hands, before you enter the house wash your hands or sanitize your hands before you touch anything in the house

ZM: Alright what about here at the clinic is there anything that has changed since the beginning of corona?

RES: Yes they changed

ZM: What changed?

RES: That what I was saying that the books are being taken once, they are now doing that you enter one -one given your medication they are no longer doing that you enter one time together

ZM: That’s all that has changed of taking….what about inside is there anything that you know that has changed?

RES: Inside yes the medications are now available especially for those on ART even if you were collecting from Murehwa they are able to give you at the clinic that you had gone

ZM: Alright, looking at the issue of the disease of corona can you say that has anything that it has affected on delivering PMTCT services looking at PMTCT cascade ,that from when the mother is pregnant registering pregnant, testing of blood, put on ART, the baby is put on ART until the baby has stopped breastfeeding, until the baby has stopped taking the education, is there anywhere that you are seeing that has been affected on those services of PMTCT because there was corona, people entered into lockdown

RES: Yes the mothers who were pregnant who do PMTCT some would give birth at home, the person you delivered her doesn’t know her status

ZM: Hmmm

RES: For baby some to be given what we call cotri to the exposed some were not even going on cotri, when they come to scale and then asked what did you give the child then they say nothing

ZM: Okay is there anything else besides medication of children, what about testing were the children being tested on time those who have been born by pregnant mothers?

RES: They were not tested on time because some could have delivered at home, some deliver at the gate so for testing on time that was not there?

ZM: Alright, What about looking at access of medication or that was given to children can you say there was a problem on supplies of medication

RES: Ahh there’s nothing

ZM: There’s nothing?

RES: Yes

ZM: What about the mothers on mothers to come and collect their medication on time like they always do, those were already new or those who were already on ART were there any challenges that happened because people were in lockdown?

RES: Yes that’s when the challenges happened that these grandmothers were taking if they say they have taken the book at 8 you have come at 9 or 10 but your date was on that day, they would tell you to go back home and come tomorrow

ZM: Alright what about the nurses did they ever failed to come to work on time or coming at all because of the issue that there was lockdown people were not allowed to travel

RES: Yes they were not coming

ZM: What was happening?

RES: Most of the clinic were being closed because of transport problems

ZM: Alright, can you say fear of getting infected with the disease did it affect accessing of services, the nurses being afraid that if we go there will be infected then say we don’t know what it will do, but affects the PMTCT services that they were supposed to give people

RES: Hmm

ZM: What was affected because of fear?

RES: They were afraid they were saying if we go the clinic it is not sprayed you I don’t know your status, when I touch your book maybe you have it then I have been infected, there was a problem there

ZM: Alright okay, what about looking in our country of Zimbabwe there were outbreaks that happened, or other diseases that came back then, we had a disease of cholera, we had disease like typhoid comparing it to what you saw when COVID-19 happened, what can you say COVID-19 disease is like or comparing it to other diseases what can you say it’s like?

RES: COVID-19 is a difficult disease because for diarrhea and cholera people were treated plus it would let’s say a person has been infected by cholera in the house or diarrhea they were being treated but COVID-19 they should do isolation, stay alone but diarrhea you were able to cook for them that water they say drink then they drink

ZM: Hmm

RES: Are things changing they will talking seeing each other but COVID-19 we will not be seeing each other they will be isolation alone

ZM: Alright looking at mothers in the community that you come from what are the problems that they encountered because of the issue that we were in lockdown or because of the issue that there was this disease of corona virus, the women that you work with in your community. What can you say are the problems that they encountered in accessing services from the clinic because of the issue that there was corona

RES: Not getting those were the days which were said those on ART were paying 1 dollar some couldn’t find money for their cards to be stamped for them to take medication

ZM: Is there anything else other problems that they encountered the mothers at homes because of the issue that we were in lockdown

RES: At homes?

ZM: Yes at homes or coming to here seek the services

RES: At home they entered into what is called GBV, hunger the father is no longer going to work the mother you are seated looking at each other at home, what are you going to give children nothing then GBV started

ZM: Okay do you think that when first lockdown happened the mothers did that have enough information about how they can travel when they want to seek PMTCT services

RES: Ah they didn’t have

ZM: Hmm what was happening?

RES: They were afraid that alright if we go out we are told to stay at homes my book cannot work because they were saying they want letters that approves that who gave you, if only they had the knowledge they would have used their books they were able to even to Parirentatwa or where

ZM: The mothers do you think when then enter into the clinic that time like those who managed to come to the clinic did they know what they are supposed to do when they arrive at the clinic to reduce their chances of getting corona also reducing chances of infecting others with corona. Did they know during that time of first lockdown that if I arrive at the clinic I am supposed walk like this and that so that I don’t get infected with corona

RES: No they did not have the knowledge

ZM: What was happening?

RES: Some were entering without even a mask, even sanitizing people we were not sanitizing

ZM: Alright can you say that you saw the number of people who were coming to get treatment doing down during the time we were in lockdown or there is no change that took place

RES: Yes it went down because those who wanted to vaccinate children they were told that there was no medication, they might be told that you come we vaccinate once if its Friday or Thursday, even the scaling of children it decreased

ZM: Do you think that the mothers at home have encountered the challenges pertaining the issue of HIV status disclosure, because of the issue that everyone was in lockdown people were not allowed to travel do you think, do you think they had GBV that has something do with status disclosure

RES: Yes status discloser even up to now it’s still there, the mother can come to take medication but the father doesn’t know the father can be coming to take medication whilst the wife doesn’t know

ZM: So GBV will happen where in homes because of the issue of lockdown?

RES: During lockdown GBV was happening on that the mother I was taking pills whilst the husband doesn’t know, the father will be staying at home he will touch everywhere every corner of the house when he don’t have anything to do that’s when he will see the book then GBV starts

ZM: Alright, what about looking at the issue of taking care of children and responsibilities do you think there is something that affected the women, because the children were now home during lockdown they were no longer going to school, do you think it affected the mother on accessing their PMTCT services the closure of school, staying of children at home

RES: No haa it didn’t affect

ZM: It didn’t affect?

RES: Yes

ZM: Alright what about looking at the issue of decision making in homes that who makes decisions do you think that there is something that was affected with the issue of lockdown?

RES: Ah there’s nothing

ZM: Alright the government of Zimbabwe has implemented different things in trying to reduce the spread of this disease , the other law that was there was that people who are at a place they must be spaced what is called social distancing, another law that was implemented was that if a person is suspecting that they are sick you must put themselves in quarantine where you will be alone until you have healed, if you are suspecting that you met someone who has this disease you put yourself in self isolation where you stay for 14 days without meeting other people, you looking at these things or these measures do you think it can happen in your community, is it a thing that people are able do ,is it feasible , is it something that is easy to do ,all those things like social distancing ,like social isolation ,like quarantine looking at the community that you work in do you think these things can be done

RES: Social distance can be done but quarantine haa can’t be done because some had built one rooms others 2 rooms if the father has been infected by this disease we use one room what are we going to do, where will we put him when we are using one room there is no solution that will be done and there no that quarantine

ZM: Okay what about what people are encouraged not to go to gatherings do you think it’s feasible that is said avoid crowded places?

RES: It’s not feasible

ZM: Why do you say it’s not feasible?

RES: It cannot be done

ZM: Why do you say so?

RES: Because if you to the funeral there is no social distancing

ZM: There’s no social distancing?

RES: There’s no social distancing even at the shops there is no social distancing

ZM: What can we do as a country in trying to help the negative impacts of COVID-19 what can be done, that corona affected many people in homes what can be done in trying fix things that has been affected by corona virus

RES: Haa it’s difficult

ZM: Its difficult how do you think they can be fixed, must it be left like that

RES: It need to be fixed because many jobs ended even those who were selling be it in town they are no longer selling, haa so the country must raise those who lost their jobs by giving them something to do

ZM: What do you think can affect people’s health that has caused by corona, that even after corona passes people would be left with unstable health , what do you think that they are some of the health of the health impacts that even corona has passed but people would be left maybe with other things that had affected their health

RES: That of being told to drink zumbani (African herb) drink this you don’t know what will damage your body when corona ends you can have disease that will emerge

ZM: Okay what can we do so that in future that if another disease that surpasses corona comes for things to keep on working well, so that clinic don’t close for people to continue getting their services well, we want to prepare that we have learned from corona that that’s what has happened, the clinics closed the mothers gave birth in homes like you were saying, what can we do in future if another disease happens, maybe be there will come another disease that we don’t even know that might be worse that corona, what have seen that this must stay in place just in case we have another outbreak for things to keep on going well. People the mothers that are pregnant being tested blood on time what can be done?

RES: What can be done is that if those facemask if they prevent the disease that is going to come the nurses must always have them those sanitizer and they must always protect themselves

ZM: What if we are not focusing on corona only maybe another disease that doesn’t need a mask comes but for the clinic not to close is there anything that can be done by the government or that can be done in the community so that the clinic cannot not reach a point where it is said that It has closed

RES: Stay awake that if the disease comes that if the disease come they will be standing where, clinic must be protected

ZM: Alright thank you those are the questions that I had

RES: Alright
