# Supplementary material for: “Getting pregnant during COVID-19 was a big risk because getting help from the clinic was not easy”: COVID-19 experiences of women and healthcare providers in Harare, Zimbabwe
Source: PLOS Glob Public Health. 2024 Jan 8;4(1):e0002317. doi: 10.1371/journal.pgph.0002317 (PMC10773929; doi:10.1371/journal.pgph.0002317)
Supplement: S1 Data — (ZIP) [file pgph.0002317.s003.zip › Data/Health Promoter/Health Promoter 3.docx]

**Interviewee’s Gender: Female**

**Interviewee’s Age: +51 years**

**Interviewee’s Initials: HP 3**

**Length of Interview: 45:24**

**HM: We want to hear from you about your experiences and what you think about COVID-19 from when the lockdown was implemented in Zimbabwe what have you faced, were you prepared or were you not what has been happening since the lockdown started the first until now?**

**RES: When COVID-19 started there was nothing we had prepared we just got in it, and we survived by hustling during the lockdown.**

**HM: You were hustling doing what? we want to learn from you what was happening during that time because lockdown has never happened before this was the first time in our country, how people survived the experiences that you encountered, and your thoughts on what you think about this disease.**

**RES: Ha ha, the lockdown was hard, to the extent that there was hunger in many homes, there were no jobs and people were not getting any money. People were surviving from hand to mouth the little money they got on the day would go towards family food. Some were selling vegetables to survive.**

**HM: All right what your name is are you married, how old are you what is your highest level of education?**

**RES: My name is XXX I am a single mother; I am 53 years my highest level of education is Form 4**

**HM: How were your experiences of coronavirus?**

**RES: During the coronavirus experience, it was hard I don’t want to lie**

**HM: Just name each thing that was hard and what you encountered, or this is what those I stay with were encountering,**

**RES: Haa we encountered difficult problems.**

**HM: What was happening?**

**RES: Because children were not going to school, child labor increased in the homesteads, and the abuses increased in gender-based violence (GBV) increased. Rape cases also with close relatives and family abusing children**

**HM: All this was caused because of lockdown, GBV and abuse what was happening?**

**RES: Because most of us were always seeing each other from time to time there was no one who was going out of home, plus sharing rooms and you leave a child with your elder son, or you leave your child with uncles that’s what made abuse increase.**

**HM: Rape, were there many rape cases?**

**RES: Rape cases were there it happened**

**HM: It happened did you see how they were handle. Were they handled in the correct way that is supposed to be taken?**

**RES: Haa we tried to take them to where they were supposed go.**

**HM: All right, the issues were solved?**

**RES: Yes, they were solved some are serving their sentences in prison.**

**HM: All right what else did you encounter during COVID-19, what problems?**

**RES: Divorces, increased and many families were broken**

**HM: During COVID-19?**

**RES: They were broken the fathers who used to come with something didn’t have anything to offer to the family. The wives started to go out to look for pieces jobs or other things to be able to survive. You can start by doing hide and seek you get by your husband. …**

**HM: Get caught.**

**RES: Get caught because she will say I’m the one who is working and taking care of the family then they will later see that the money that she’s coming home with is from selling sex.**

**HM: All right, did COVID affect you personally on the way you think or your mental health in general in any way?**

**RES: Haa it affected me on the fact that I am on ART so the medication that I was supposed to be getting like Cotri I couldn't get them, for me to buy I didn’t have the money to buy.**

**HM: They were no longer given at the clinic?**

**RES: At the clinic, they don’t have even now they are saying there's no Cotri.**

**HM: So, it affected your health?**

**RES: They say we should keep on taking Cotri for the sake of our immune system**

**HM: Immune system?**

**RES: So, there was nothing if you couldn’t buyt it, you will be like maybe today you have a headache tomorrow stomachache those are some of the things we encountered**

**HM: Did COVID-19… affect the way you think, and thoughts did it affect anything?**

**RES: Hmm in affecting my mind**

**HM: Hmm**

**RES: What affected me is that life was ending, people were dying and families were lete shuttered. You would hear that so and so is sick tomorrow or 2 days you would hear that he/she is nomore.These are things that you will saying what if it infects my family what am I going to do,it disturbed me.**

**HM: It made you have stress or depression?**

**RES: It made me haver stress because…..**

**HM: Or metal instability that you will hear that something had happened?**

**RES: I ended up getting sick because I have a sister who died because of COVID**

**HM: So you entered into depression and stress?**

**RES: Yes**

**HM: She died because of this disease?**

**RES: With COVID yes**

**HM: Alright**

**RES: But she was not staying in XXX she was staying in Dzivarasekwa**

**HM: Alright, that’s where she contracted it??**

**RES: That’s where she contracted it**

**HM: Alright, we want to move on to you job, can you tell me about your portfolio of work, what do you do here as a health worker or the work that you do here at the clinic**

**RES: Here I am a community linkeage we see those who take ART and children we see if they are taking their medication correctly we do what is called adherence support, we also track defaulters**

**HM: Hmm**

**RES: If we track we bring them back if they are willing to come back on ART we come back with them then they go for counselling then they will be put back on ART**

**HM: So were you able to do that during COVID during lockdown?**

**RES: We were not able**

**HM: To do follow al those follow ups**

**RES: We were working online with…**

**HM: With the people were you reaching them?**

**RES: Yes were doing on the phone we were calling them**

**HM: What about now have you started work fulltime?**

**RES: It’s now better we not yet started full time but its better**

**HM: Alright let’s look at health context in our country of Zimbabwe right**

**RES: Hmm**

**HM: Can you explain to me how you work here looking at the health issues or health organisation that you work in are there any problems that you are facing now on heath there you are a health worker right?**

**RES: Hmm**

**HM: Can you explain to me what you are encountering looking at the nature of your job**

**RES: For us I cannot say I am a VH as such I am a case care worker CCW**

**HM: Okay**

**RES: So I am not too based here I came because of community linkeage I am based at Makombe**

**HM: Alright in town?**

**RES: Yes at Makombe but we work in the area**

**HM: Alright what exactly will you be doing?**

**RES: Will be looking at protecting the rights of children**

**HM: Alright**

**RES: Protecting children**

**HM: What else do you do with children?**

**RES: Children we teach them**

**HM: You work with what ages from what age?**

**RES: From an infant to 18 years**

**HM: Hoo up to 18 years what are the main things that you tuckle when you will be working with children?**

**RES: We will be teaching them their rights and responsilities also we will be encouraging them to……what can I say we will be asking if other children….some parents are not able to open up about the issues to do with ART so if know that the child….we work with children who are HIV positive we encourage them so that we can be able to come with them to the grandmothers that are here and be seen**

**HM: Hmm**

**RES: If we saw that the this child…..most of the time we do concern first that the child must know what the guardian id doing, so if we go to the parent first we talk to them if they agree and allow then they will come with thir child for testing after tested if we see that the child is positive we put him/her……will be put in support groups that others has entered they we will track what we call adherence support**

**HM: Alright were you said CCW who will you be working with with ministry of health or what will be happening?**

**RES: We are under social welfare**

**HM: Hoo you are under social welfare but but some cases of the cases we encounter they will be wanting you to work with the clinic or with health workers?**

**RES: Yes we go everywhere we work tohether**

**HM: You work together with Ministry of Health?**

**RES: Ministry of health and other stackholders we help by referring were we are supposed to refer,there are some that may be need food we go there, were there is need of police we go to the police, were there is need for the clinic we bring to the clinic, were there is need for shelter we go to Makombe**

**HM: Alright,** **what measures or changes are you making, are there any measures that you are taking or changes in response to COVID-19 looking at the issue of COVID-19 are there any measures that you are taking on your level personally, as an otganisation lets start with you personally are there measures that you doing ot changes that you are doing looking at COVID-19 this disease you personally?**

**RES: Personally what I was doing during lockdown is staying at home and protecting my family so that they don’t play outside and staying wearing face masks.**

**HM: Is there anything else you personally that you are doing that you would want to add on or that’s all that’s all you are doing so that the disease doesn’t keep on spreading**

**RES: Yes that’s what I am doing so that the disease doesn’t spread us not traveling**

**HM: What about your organisation as CCW are there measures that you are doing or the changes that you have done because of COVID-19?**

**RES: We were able to communicate with the parents on their phones we would have their numbers,so we would tell them doing like awarenesses that childrens rights,the importance of staying with your child,enlightening them that COVID is there for sure,COVID is killing ,protect children ,children must not go and play were you don’t know,stay with your children in good manner,don’t beat children beating children is not a solution you talk to the children**

**HM: Alright,** **how do you cope with the measures or the changes that happened how are you coping at your work, at your home?**

**RES: No this is good because now we are seeing a change because now here lets say in this community the last days people died here but its now better the has decreased**

**HM: So these measures are they working that you implemented or they are not working?**

**RES: No its working**

**HM: It’s helping?**

**RES: Yes it’s helping**

**HM: How about at work?**

**RES: It’s helping as well**

**HM: You are now working well?**

**RES: Yes we are working well**

**HM: Alright the health workers from your pesperctive how are they seeing the problem that has been brought by coronavirusfrom you point view?**

**RES: Haa I see like its was hard, as hard as it is for us especially to those who are here**

**HM: Nurses?**

**RES: They are on risk because the time when it was said there is COVID in XXX it took time to the extent that this place closed**

**HM: Hoo they closed?**

**RES: They closed saying that they found a person who tested COVID what what, so these are some of the things that hurts us that some of us has been infected with COVID at the facility what about us ,they are the professional what about us**

**HM: What am I going to do?**

**RES: Hmm**

**HM: That what they encountered?**

**RES: Hmm**

**HM: Alright you said it was closed what about the people who wanted help what were they doing?**

**RES: Haa it was hard here it was hard people were looking for informal midvwives home midvives**

**HM: People were giving birth in homes?**

**RES: People were giving birth in homes some of the midwives are those…**

**HM: They don’t even have any education or taught?**

**RES: They dont even have education on that they just say come let me deliver you**

**HM: Alright so what were they doing on PMTCT services those preganant mothers?**

**RES: Nothing what were they supposed to do, there was nothng that was done**

**HM: So that the children can be protected from being infected with HIV it is said that a preganant mother is supposed to come here to Anti natal care and get checked and given pills if they found that they are HIV positive they will be told to go on ART so that they don’t infect the child so what was happening?**

**RES: Here what they were doing is that if a pregnant woman the one who knows that she is HIV positive,the day when her pregnant is due when she came here that is if its open because most of the time they were saying that at 4.30 they will be closed**

**HM: Hmm**

**RES: When it’s open that when she was given that pill Nevirapin**

**HM: There once only when she is delivering?**

**RES: Yes**

**HM: What about all along?**

**RES: All along they were told that they should stay in their homes then come when it’s due**

**HM: There are no services that they were being given?**

**RES: No these were no services haa it was hard**

**HM: Alright what about getting tested and being given medication were they available during COVID for preganant mothers?**

**RES: Medication for ART?**

**HM: Hmm**

**RES: Medication for ART was availble everyone was getting their medication**

**HM: They were coming to collect?**

**RES: Hmm you would come to collect you medication and go back home**

**HM: Alright so who had problems are those with new pregnancies that needed to be tested so that they can be seen that they are**

**RES: That’s right**

**HM: But those who knew that my status is like this they were getting their medication on time?**

**RES: Hmm**

**HM: Alright, so were there others who were new who had not registsred or those who were able to get medication are the ones has been collecting already?**

**RES: Those who were collecting are the ones who are on ART already**

**HM: The new ones there is no one who was**

**RES: Because the services here at the clinic you would arrive here and hear that there are no nurses maybe there are 2 nurses so 2 nurses one will be at OI the other one at martenity so inside there is no one who doing everything**

**HM: No one is doing everything**

**RES: Hmm**

**HM: Alright what about those who were taken…lets say they were taken blood was it going for tests on time or all this was not happening because of COVID-19?**

**RES: I don’t know because during lockdon we were at home,we were at home they said you should not come to the facility so I don’t know if there are any who would come for tests and if their blood was going but what I know is about those you were already on ART**

**HM: What about workers how often were they coming to work?**

**RES: They were exchanging they were exchanging they were nolonger coming together**

**HM: Alright they were nolonger coming together?**

**RES: They exchange coming**

**HM: Were they afraid of getting infected with COVID-19 did they had fear what was happening?**

**RES: I think its fear everyone is scared of death, they were afraid dying leaving their family**

**HM: Or they were afraid because they didn’t have enough PPE to wear to protect themselves?**

**RES: Everything nothing….lets say you fearing for your health as well so it was hard for them**

**HM: So they were not coming?**

**RES: Hmmm**

**HM: Limited number was coming?**

**RES: Limited number yes you would arrive at the facility then ask if there are nurses they will say there are no nurses, like treatment the emergenicies were the ones who were being helped fast,saying that I have a headache they would say go and say that at the pahamacy**

**HM: You buy there?**

**RES: Hmm**

**HM: Alright**

**RES: Because the screening was done at the gate**

**HM:Do you think comparing this disease of COVID and other disease do you think COVID is a disease that has brought many problems or other diseases that happened back then like typhoid,cholera were better,looking at..Comparing other diseases that happened back then to COVID**

**RES: These ones were better they could be treated fast haa COVID was advanced, its advanced we can’t say it was bacuse COVID is still there**

**HM: Yes because if we look at typhoid, cholera and other disease that we have encountered**

**RES: Here in Mbvuku typoid entered but it was treated and it ended,you would hear that do not walk around this section it has people with COVID then people from Health they will go there and spray, people were treated in their homes**

**HM: With cholera**

**RES: Cholera they were taken with a cage they were taken to Nazareth or Wilkins but it would pass those who would…..theyre time will be due this one haaa**

**HM: Alright I want to explain how your job has been affected because of coronavirus, what are the changes that took place at your work because of coronavirus, the services that you were giving that failed to be happening because of this disease of corona**

**RES: The services that we used to do of awareness campaign they stopped opto now we are still stopping**

**HM: They are not doing awareness campaigns at all?**

**RES: Yes we are doing awareness on phones**

**HM: On the phone how many people are you reaching now?**

**RES: Nothing we reach to those caregivers who are available that we have reached and the family but those we didn’t not reach they will not be aware of anything**

**HM: Alright what else?**

**RES: What changed is that we were nolonger coming to work that like we used to do.We were nolonger coming to work we were seated at home even the money was not there that 2 cents you used to get,we were at home so why would we get paid whilst we are at home**

**HM: Nothing so all this was happening because you were not able to come here because of corona?**

**RES: Yes that right**

**HM: Alright, what do you think are the challenges that were encountered by women in trying to access PMTCT sercices during lockdown, what challenged did they encounter?**

**RES: The challenges are they were not able to come to the clinic there was no….it was closed so that is what was troubling them that alright how were they going to know they’re status**

**HM: So they were not getting any help?**

**RES: There was no help yes**

**HM: They were able to get?**

**RES: Uhm**

**HM: Do you that the patients that were there or those who were sick that time of COVID were able to get the information that they needed during the time of COVID?**

**RES: Ahh they did not manage to get**

**HM: Hmm why do you say so?**

**RES: Most of then did not manage to get because there was nowhere to find it,most of the patients they didn’t had anywhere to find it ,if you get like what I was saying that if you reach that one person that’s it what about others who is going to tell them**

**HM: Alright,so those PMTCT women had no idea on what do during COVID time during this lockdown?**

**RES: That’s right**

**HM: There was no one who could help them?**

**RES: They…those who registered to private hospiitals were the ones who got help because the doctor have commited themselves they were always open because doctor is a surgery they will be wanting money,those who have registered like us who don’t haver money we want local clinics it was difficult**

**HM: But did there know where they can go to get help?**

**RES: To know where the are supposed to go?**

**HM: Yes that they can get help during lockdown looking at women who were pregnant**

**RES: Getting help or when they are due to give birth?**

**HM: Hmm**

**RES: When they were due most of them were being returned at the gate like I said some were giving birth at the gate when the gate will not be opened then people deliver their babies at the gate**

**HM: Alright**

**RES: So it was difficult**

**HM: What about coming to the clinic or traveling was it easy?**

**RES: Haa it wasn’t easy**

**HM: For person to go to the clinic a mother who is pregnant was it easy for her to travel, the restrctions of traveling were they able to travel?**

**RES: The issue of traveling those of the clinic..**

**HM: The women who are pregnant**

**RES: If you had a card that was showing that you are going to the clinic you were able to travel but if you didn’t have a card no it wasn’t coming out**

**HM: Police was not trobling along the way?**

**RES: Noo for health, they were troubling those who were lying because they know that everyone says im going to the clinic, so they wanted to see a card that you are going to the clinic no they were allowing you to travel but at the clinic you would be returned because no one will be there**

**HM: It will not be opened?**

**RES: Hmm**

**HM: What about on the day they say it opened when they came were they how did they operate?**

**RES: The clinic admin staff will go to the gate and collect the patients’ cards, and after they look for their green cards and check them. They then go back to the gate and start to call their names out whilst everyone is listening, and they will be a lot of people there. It’s like forced disclosure because you have no option because everyone else who will be at the gate will get to know that so and so is on ART. In the communities you hear them saying they will not come until COVID-19 is over but who knows when this will go away, and you can imagine what will happen to those mothers and their babies**

**HM: Alright so you said maternity was the only one that was open and OI**

**RES: Yes**

**HM: Everywhere was closed?**

**RES: Yes**

**HM: Alright**

**RES: And at pills**

**HM: It was open?**

**RES: It was open because you were given your pills after coming from OI, but they reached a time when the pharmacy was open you were given you pills there**

**HM: You were collecting everything at one place?**

**RES: You were given there and there then you go out and go home**

**HM: So they were checking temperature and all at the gate before you enter?**

**RES: Temperature and sanitiser**

**HM: At the gate?**

**RES: At the gate or washing your hands with running water and soap**

**HM: Do you think the number of people who were seeking for help at the clinic or the women who were preganant decreased or are there any changes that took place on the number of people that came to the clinic to get help especially pregnant women during this period of COVID-19?**

**RES: Haa many people didn’t come it decreased because you would come wanting to register,wanting to register scale they could tell you that we want 5 only today we are taking 5**

**HM: These are people who were preganant?**

**RES: The rest they go back tomorrow they wake up again**

**HM: When they were saying some go back which criteria were they using to know that this is the one we are supposed to attend today?**

**RES: First to arrive they would just do that 12345**

**HM: Hoo first come first serve**

**RES: Hmm**

**HM: What about others what were going to do the ones who came late but wanting?**

**RES: That’s when others don’t come they then pay the informal midwives, they will end up saying there is no help at the clinic its better for me to pay these informal midwives**

**HM: Hmm alright**

**RES: I can pay there in advance that is its due they just go there**

**HM: Hoo that’s what was happening?**

**RES: That’s what was happening because haa a lot of people were being returned if they say today we want this number, today we want 5 we want 10 maybe you are 20 or you are 30 then you go back and see the time you woke up**

**HM: It’s not tallying**

**RES: Especially young children they would loose hope but the grannies those who are a bit older not saying grandmothers because there is age of giving birth they were the ones who had patience not our young children they don’t have patience**

**HM: Theres no patience**

**RES: They will go back and say whatever, what ever happenes or when it’s due than I will just come**

**HM: I wil come then will help me?**

**RES: Yes**

**HM: Alright so it was hard**

**RES: Haa it was hard we don’t want to lie during COVID it was hard, COVID affected haa it was hard**

**HM: Alright are there changes now since lockdown has been raised like this on the number of patients who are pregnant who are coming to seek help at the clinic is there change that you see like now things are…**

**RES: Ahh now things have changed even coming to scale they were nolonger allowed to come for scale they were told tyo come when its due so now they were given timetable that in the morning we start registering this time at 11-12 you come to scale it has changed**

**HM: So the number has increased of people who are coming to get help**

**RES: Yes it has increased even delivering in the afternoon people are getting and those who comes at night they are entering but those who have accompanied you no one enters,those who have accompanied they will leave you at the gate then you enter alone**

**HM: Alright, in your opinion looking at how people are living at home do you think preganant women were affected more on saying out their HIV status these preganant women**

**RES: Saying at their homes?**

**HM: To disclose status at home**

**RES: Haa most of then didn’t open up the women they didnt**

**HM: They didn’t open up why, why are you saying they didn’t open up?**

**RES: Because most of them did not continue coming to get educated some were like they get tested today they get education on that day so it needs to be …**

**HM: Continuos**

**RES: Continous so that they be able to open to your partner so it was hide and seek only**

**HM: There was no truth that was coming out?**

**RES: There was no truth that was coming**

**HM: Was it heavy to tell their husnband that I went to clinic and tested because I am pregnant I was taken blood the results came out like this**

**RES: Some of them they would tell you that….like it happens in my area the husbands would tell you that I don’t have HIV its yours you are the one who get it I don’t know were you got it showing that the baby that you have is not mine, this issue was problematic until they go for DNA then they saw that the child is his, they later discovered that this man has been taking pills at work**

**HM: He was hiding**

**RES: He was hiding then he didn’t want co come home with them to tell his partner that thas who he is**

**HM: So he was the first but he didn’t disclose**

**RES: He didn’t tell his wife**

**HM: Alright, so this issue of disclosure was difficult people were not telling their pregnant wives, the women were not getting support because you said they were supposed to continue coming for counselling and taught that the status has come out like this you are supposed to**

**RES: You will go like this you will go like this**

**HM: What about looking at child care during COVID, child care roles and responsibilities how do you think the women were doing it?**

**RES: Haa women did take care of children thats what I saying that it was child labour, the mother would wake up and seat then think of sending the child to go and sell vegetables saying if you don’t sell I don’t what you will you eat, you would see the child during this time we have water problems, you would see the child who is not supposed to be carrying a 25 litres of water will be carrying water**

**HM: What about the children lets say who were on ART who were born positive were they taken care of to see that their medication has been collected on time,they have eaten the food that they were supposed to eat for them to be able to take thier medication**

**RES: It depends with the parent what bond do they have with the child because some they don’t even have bond with children they will be saying whatever,they will go oue leaving that child but they will be knowing that shes the one one who gave birth that child they go out**

**HM: Hmm**

**RES: For the child to take medicationon on his/her own he might take but that will not work maybe the child was supposed to take medication 7 they will take at 9 tommorow at 12 so it wasn’t helping**

**HM: It wasn’t helping**

**RES: Hmm**

**HM: Alright what about on access and control of resources these women were they getting chance to get money and getting control of resources so that thins can be moving well?**

**RES: Haa no**

**HM: Why do you say so?**

**RES: No we were seeing some you would see with your eyes that here it’s hard there was nothing**

**HM: What will be happening?**

**RES: They can go from morning without eating anything you know,the whole day seating seeing that hunger is killing my partner telling you I don’t even have anywhere to start with**

**HM: Men were they being stingy with their pockets or will be no anything at all**

**RES: Sometimes there will be no anything sometimes we cant keep on saying men are problem no there will be no anything they will be saying I tried to look for something but I ddint find you start by getting vegetables from the until until its reaches the heart they you wll see that ehh**

**HM: There’s nothing there’s nothing**

**RES: If you beg this tomorrow you beg this you will get tired that you will see must the whole line know that I am begging for food**

**HM: What about on decision making who had more powers?**

**RES: Talking**

**HM: Thinking giving decisions planing this and that**

**RES: Home?**

**HM: Home**

**RES: Haa this was one man for himself**

**HM: One man for himself each was doing whatever they do**

**RES: They were doing one man for himself that’s why many divorces came out**

**HM: Yes**

**RES: No they were doing one man for himself**

**HM: Why are you saying so?**

**RES: Maybe the husband has gone to look for a dollar i don’t know the deals that he did they came out in a short period of time he will come and keeps quite**

**HM: He doesn’t tell that I have found money**

**RES: If the wife go to do laundry peace job she will not tell the husband that I got this, its like if she find something she will buy whatever she buys without telling anyone that this is what I have done if the other one sees that’s what you have done to ask where you have find that was not being done**

**HM: Alright what about at community level decision making powers how was it like?**

**RES: Haa people were not seeing each other so here nothing to say, each was at their place those meetings we were doing support groups what what they were not there we had stopped, that if they are to start suppot groups they are starting this weekend all along since last year there were not there**

**HM: They had stopped?**

**RES: There were no we had stopped all that**

**HM: Alright because of COVID?**

**RES: Haa Haa coronavirusis very scary**

**HM: Zimbabwean government has implemented major social changes including social isolation, people were nolonger allowed to meet each other, people are nolonger allowed to walk around,closure of schools,closure of borders how much did that impacted women who stay in your community**

**RES: This caused the women who were used to hustling alone you have your passoport they say we are going to Mozambique for bales, they know that I am going to South Africa for groceries or to do what or to work those who work there these was no all that**

**HM: Hmm**

**RES: It also caused that those women some of them they went and left families and the husband they went for good**

**HM: They didn’t come back?**

**RES: They didn’t come back**

**HM: Alright so most of the women in your area they…**

**RES: Some are still there in South Africa upto now**

**HM: They haven’t come back?**

**RES: They haven’t come back they are just saying we will come back**

**HM: They said they will come back?**

**RES: We will come back they left families who being taken care of by grandmothers, the grandmothers are old don’t have anything to give children**

**HM: So do you think that in your community these measures that was implemented by the government were helpful that they are nolonger allowing social gatherings, at funerals they nolonger allow many people, at churches they are no longer allowing many people or we are closing, traveling is nolonger allowed, closure of schools and borders, do you think it helped for te disease not to spread in your area**

**RES: It helped because if they didn’t do that by now there could have been nobody in our community because here there are people who like to party, there are families that like parties**

**HM: Alright**

**RES: Plus school children others came from homes were they do parties it’s a gathering right there will be many, then they will go to meet other children at school they don’t know who has COVID and who doesn’t, on that the government did well**

**HM: Alrightit did well, now what measures or programs that can be done to reduce impact of COVID-19 in your community what measures or what programs that are needed or that can be done to reduce the impact of COVID-19**

**RES: Now we can say that if they can continue maintaining that number, they continue maintain small number, few few no gathering they should stop**

**HM: They must remain on 30 funeral 50 only**

**RES: Only they must not exceed that also when we come here at the facility social distanc must keep on being maintained, facemask, they must keep on following washing hands must be like that**

**HM: What else can we do measures that can be done to protect or what can I say to mitigate negative impacts of COVID in this community,measures or programs that you think can be done so that impact what is being caused by COVID in this area to be reduced**

**RES: They need awareness campaigns but those of moving around not**

**HM: Gathering people**

**RES: Those of going around talking with a...**

**HM: Speaker**

**RES: With a speaker in a van going around that might be helpful**

**HM: Alright what are the disease that you think or health impacts of COVID-19 in this community without looking at closer things but looking at the future, that COVID-19 what are the problems that are going to affect us because of the disease?**

**RES: This disease the isue is that some of the problems are that education of children, learning children didn’t learn, this will cause problems in the futre is that the children was supposed to be somewhere might fail**

**HM: Hmm**

**RES: Because some of the jobs that they were supposed to do, people were supposed to be doing those jobs on that level cannot reach that time they will be a gap**

**HM: What about looking at the issue of health?**

**RES: On health I can say that if they continue following like HIV/AIDS if you continue being on me ART I don’t see any problem because you will continue getting medication as it is there noway you are going to break that there in no medication except if they had said that there is no medication but if its there you will continue taking your medication**

**HM: You will continue getting, there are no problems that we are going to encounter in future because of COVID-19?**

**RES: Ahh that is if you have been infected by it…**

**HM: This disease can happen now right but in future we will feel the effects of it or its impact but maybe here it may have ended, what it left for us that will affect us in future**

**RES: Haa**

**HM: Is it there?**

**RES: Haa I dont think so**

**HM: There is nothing**

**RES: Hmm**

**HM: If it has ended it has ended?**

**RES: If it ended lets say they have found mediation**

**HM: Are ther no other diseases that people will be sick with after, those who have been infected they might have healed now but it will keep on troubling them in future, is there anything that we are going to encounter?**

**RES: Stress and depression that we have been talking about youll be thinking that COVID did this and that that year COVID did this on health, on my family or on my relatives**

**HM: That what will be there?**

**RES: That what will be there myabe those who had lost their relatives back there they wil be thinking that if they had find the medication quickly maybe my so and so would have been here we will be thinking about that in future**

**HM: Alright, do you think that if the COVID ends in future what will it cause on the issue of social gatherings are things going to change after COVID has ended that will people go back to normal life they were living or it will be different things will never go back**

**RES: Haa it will be different**

**HM: On short term what do you think will happen?**

**RES: Haa for them to go back to normal now there is still time**

**HM: They will not go back?**

**RES: Haa they will not go back now haa there is still time for them to go back to were they were haa there is still time**

**HM:What about looking at the issue of wealth,finding money and working for ourselves and what the COVID impact do you think if COVID has ended will things be back to normal or on economic issues**

**RES: Haa it will take time because many nolonger had capital, they didn’t have anything to do so for you start rising again it’s a problem**

**HM: It will take time what about looking at the issue of getting jobs?**

**RES: Haa there are no jobs**

**HM: So will it be affecting on long term or short term that for us to be able to say our country has gone back to normal things are now okay or our families to gone back to were we can say things are okay**

**RES: Haa it is long term it can’t be short term it canr be short term**

**HM: Alright what do you think are some of the things that you want then to be done as a national response, response as a whole country looking at this disease of COVID-19 or coronavirus pandemic, what do you recommend that this as a country if we do this and that on national level it will help to stop this disease from spreading**

**RES: I can say that since they are saying that there is medication the injections that came I don’t know them but we are hearing that injectins, if its possible if we know that they are true injections I don’t know but every one must get vaccine for it to do because if they vaccinate 50 and the other dont get vaccinated haa it will not come out**

**HM: It will not come out is there anything besides that everyone must be vaccinated what can be done by the country as response to COVID-19 that haa I recommend or I think if the country can do this or that looking at the disease of COVID-19**

**RES: The country must help people with…..those who want to do hand jobs they must be helped so that they can start hand jobs, those with companies already if they are registered they must be helped with money frm the government for them to be able to rise that’s what I think**

**HM: What about on preventing the disease what do you reccomend that the dovernment must do this so that COVID-19 can end, what you think that I think that if the government could do this and that we can won on COVID-19**

**RES: That what I am saying that everyone must get injections**

**HM: Injections only is there anything else that you want to the givernemnt to do?**

**RES: The government if it could give everyone like what is it called …for us to keep on having them**

**HM: Masks**

**RES: We must keep on having them those who work in clinics must have PPEs**

**HM: Enough**

**RES: Enough I think it will do those who work in schools they must get enough to prorect themselves and also the children they teach**

**HM: They spend the days with**

**RES: Yes**

**HM: Aright, what measures or programs that you want to be put inplace to end the negative impacts of COVID at works,in community,what are the measures that you want them to be done in trying to end the problems that were brought by COVID-19 in community or at work**

**RES: At works its that we should continue maintaining social distance,keep on using hand sanitisers,keep on using masks,for them to be able to get equipment to use within workplaces so that they can protect themselves from COVID-19**

**HM: Equipment like what?**

**RES: Something to wear especially to wear that need to be protected because you will wera then take it off and leave it here**

**HM: What about in communities what are the measures or what programs that can be done to reduce the negative impacts of COVID-19 in community?**

**RES: In the community like I have said they can do awareness telling them to stay in their homes,do not travel ,if you are foing out carry your mask your sanitizer if you are able to have your small bottle if you don’t have when you aarive at a place were there is running water was your hands**

**HM: That is what is supposed to be done in the community?**

**RES: Yes**

**HM: Thank you with your time those are all the questions that I had pertaining COVID disease also pertaining preganant women that were they taking services during lockdown those who wanted them were they getting them what was happening**

**RES: That’s nice thank you**

**HM: Thay you let me hear how is the money going like**

**RES: There are still other 2 outside**

**HM: Yes they will come in, alright mother you can enter there they want to take you details in that room**

**RES: Alright**

**HM: Thank you mother**
