# Supplementary material for: “Getting pregnant during COVID-19 was a big risk because getting help from the clinic was not easy”: COVID-19 experiences of women and healthcare providers in Harare, Zimbabwe
Source: PLOS Glob Public Health. 2024 Jan 8;4(1):e0002317. doi: 10.1371/journal.pgph.0002317 (PMC10773929; doi:10.1371/journal.pgph.0002317)
Supplement: S1 Data — (ZIP) [file pgph.0002317.s003.zip › Data/Health Promoter/Health Promoter 5.docx]

**Interviewee’s Gender: Female**

**Interviewee’s Age: Around 50 years**

**Interviewee’s Initials: HP**

**Length of Interview: 47:22**

ZM: First I am going to ask you to explain to me a bit about yourself, how old are you, are you married, you work here at the clinic as who?

RES: Let me draw near, my name is XXX, I am around 50 years old, I am a widow my husband passed away,

ZM: Hmmm

RES: I am HIV positive I was tested positive in 1999, I have 1 child, but I had 3 children 2 passed away in 2000…….1995 and 2000 because PMTC was not yet there so I am left with 1 child who has 2 children which means I have how many grandchildren

ZM: 2

RES: 2 grandchildren

ZM: Congratulations

RES: Is it

ZM: Yeah

RES: Here at clinic, I work as a community health worker, I work on voluntary basis, I do households visits, I work with children, I also work with women and especially people who are HIV positive, adolescents who were born positive and adults who are living positively.

ZM: Umm

RES: Also encouraging adolescent girls or mothers who are pregnant to go and register for PMTCT. I did this because I’m one of the people who lost children, it pushed me to help others.

ZM: Hmm

RES: I feel the pain if a woman who is pregnant fails to seek these services because if it was there during the time, I was pregnant maybe as we are talking my children would have been here, so that’s what gave me the passion to work in the community as a volunteer.

ZM: Okay looking at the COVID-19 pandemic, you as a health worker how do you feel?

RES: Hmm this disease it’s a very difficult disease if I look at other diseases or other pandemics, they did not reach this stage, especially if you look at HIV/AIDS virus the way it spread is different ifrom how the COVID-19 is spread

ZM: Where is it different?

RES: It is different on the symptoms of COVID-19 to come out its different from HIV, with HIV you will be fit and living your normal life and people would not know thatyouare positive. With COVID-19 you will isolate yourself, you will be staying isolated alone and you will be doing social distancing, now think of it school children are not going to school.

ZM: Hmm

RES: With HIV children didn’t stop going to school, and the cloths that we put on our mouths (Masks) and HIV didn’t stop people from talking to each other and doing social distancing.

ZM: Hmmm,

RES: Yes, we say people must protect themselves so that they don’t get HIV, but there is nowhere you have heard that people were told to walk wearing condoms or men walk around wearing condoms or women were walking wearing condoms meaning that this disease is deadly

ZM: All right, looking at you as a health worker who works with people and at the clinic can you please kindly share how COVID-19 has affected you personally?

RES: A lot

ZM: Hmm

RES: Because I saw myself as one of the people who were at high risk and with my HIV/AIDS status, I saw that my immune system was compromised I had a high chance of getting infected with this disease.

ZM: Hmm

RES: Its something that really traumatized me, I was thinking that if got infected with HIV and if I get infected also with COVID-19 I survive will I survive, these are things that troubled me to the extent that I was afraid to do my work wholeheartedly.

ZM: Was there a time you stopped doing your job because of stress or feeling unstable?

RES: Yes, I stopped going to work whilst assessing the situation, we fear even to do follow ups in households we were just seated at home.

ZM: Hmm

RES: We spent a long time seated athomewe were afraid

ZM: Okay going back to your job as a health worker, I want you to explain to me that your job comprises of what, what are the things that you are expected to be doing as a health worker?

RES: As a health worker I am a community linkage meaning that I work with the community helping them to come to the clinic. Most of the time will be in the community.

ZM: Hmm

RES: So, I can say that I work as an intermediator I am in between, when I am at the facility at the clinic those who have failed to come to collect their medication following then at their homes doing what is called adherence support, visiting them seeing whats going on with the person.

ZM: Hmm

RES: Then at home those who have problems who need cycle social support I will be counselling and making them return back on what on ART or for them get help.

ZM: Alright what about looking at pregnant mothers your job focuses of doing what?

RES: My job focuses on that I’m there to see 1. If the mother who is pregnant has registered 2.also has she got the services that is if she was found positive that she is supposed be what to be given, also we know that the other way of mother infecting the baby is that baby can get infected by the mother by 7% what we call PMT what mother to child transmission

ZM: Hmm

RES: So I will be seeing if the mother has followed all the stages that are needed, also I am there to give them education that they know that the baby in their stomach is at risk how on getting the virus from the mother, also the baby is at risk through the mother who is pregnant when giving birth and when breastfeeding

ZM: Hmm

RES: Those are some of the things I see that I have helped the mothers

ZM: Alrigt okay, looking at now that there’s COVID-19were you given other duties that were not doing before that you were expected to be doing now,pertaining COVID-19that you already have your work that you were doing before this disease come but now because this disease came is there anything that has been added another work load on top of your job that you were already doing

RES: They added we have somewhere that we are reporting what we call COVID-19Awareness I am now sending awareness messages to the mothers, to people in the community so that they know that COVID-19is there,

ZM: Hmm

RES: And also still on COVID-19we are doing what we call sensitization of teaching people about COVID-19that how is it standing, especially at the boreholes we are there teaching, discussing with them about this pandemic

ZM: Hmm

RES: In the church we are there again teaching people in churches that it’s standing like this, be it it’s a funeral we are supposed to be there teaching about COVID-19doing awareness that people may know

ZM: Hmm

RES: And also that are people are following the COVID-19measures that are required, to see if they are doing social distance, are people wearing masks, are people not doing gathering tha are exceeding the required number that is supposed to be done

ZM: Alright

RES: The work load has increased because we are now doing COVID-19on the other hand we will doing the one that we have been doing

ZM: Okay lets look at you you have been a health worker for a very long time, its been a long time as a health worker here without focising on the COVID-19disease but looking at the way they are doing health services in our country how can you say you are seeing that context ,is it impressing you the way they are doing the services or looking at health situation in Zimbabwe now can you say there are any differences from back then and now or looking at that context of health the way people are being given services at the clinic and treatment of people in Zimbabawe without focusing on women but looking at how people are being treated in Zimbabwe are you satisfied ot there are places that you are not satisfied with?

RES: Ah there is a big difference I am telling you that I was tested in 95 and I started ART 2008 you were given time that as a person you can prepare yourself that I have commited myself that now I am going on ART, now they they are doing what is called fast track they just test and treat, and because of resources, because of sortage of nurses or the doctors a person is not given long time to prepare

ZM: Hmm

RES: Through that people are going to collect medication through force, so for them to do adherence and follow exactly what is needed they not doing what they are not able to do that you understand

ZM: Hmm

RES: There is no counselling at first you were given what was called HIV basic 1 and basic 2 ARV 1 ARV 2 to prepare a person, for a person to prepare to take their medication but now if you get tested and they found out that you are positive here are your bottles go and take what medication you understand

ZM: Hmm

RES: Then it comes to again that these days they have brought what they call self testing kits that a person can test him/herself at home, back then before you get testyed they would start by going through what they call pre-test counselling they start by testing you to prepare that when you get tested what are the things that you are supposed to look forward to, then there was post test counselling after that being tested about what you prepare when receiving your results

ZM: Hmm

RES: You were given a lot of time to do all that, so now they are not able to do that so it’s a work again to us health workers backlog that a person comes without fully baked in counselling,we will then follow that person giving them counselling and giving cycle social support

ZM: Hmm

RES: So you see that it’s now different, also at the clinic you would arrive and get served, your services were there you get help then you go where home

ZM: Hmmm

RES: But because of what is happening now if you would had come early you would see people crowding outside the gate, there is no one who is allowed to enter but they are entering one one or 2 or 3 the rest you spent the whole day on the sun out side

ZM: Hmm

RES: And these days there no confidentiality, you cannot hide it back then you know that if I arrive I am going straight to the medication if I am going to OI I am going straight to OI,

ZM: Hmm

RES: Now they go outside they take the grandfathers they take cards they go with them and take your green books they check everything were you coming to review what what they go back again then they call so and so they are outside people are crowded there they are mixed

ZM: Hmm

RES: Maybe its mother of so and she not supposed to know that I am on ART youre now doing forced disclosure,being forced to disclose there are people which yoou don’t want to do,meaning that some people will end up not coming to seek these services

ZM: Hmm

RES: Like last time there was a person, 2 men they’re cards were being called so and so they were nowhere to be found until I said grandmother and grandfathers these people that you are looking for they are there but the issue is that they way you are doing it, that’s why people are not answering their names, they will wait until everyome has finished they will come saying we have came

ZM: Hmm

RES: People are allowed to do disclosure stigma and discrimination is not ending because if you get called that those for viral load everyone will be knowing, those who have come to review the person is nolonger feeling comfortable

ZM: There is no comfort

RES: There is no confidentiality you understand

ZM: Hmm

RES: It’s now different by far

ZM: What about looking at children are there any differences that you are seeing that if a woman gives birth is there change that you are seeing that is happening in our country without looking at COVID-19but what is happening the way people are being given health these days in the clinics, is there anything that has changed focusing on women who are pregnant, focusing of new born babies and under 5

RES: Yes you are seeing that long back we would arrive the scale, to register they would say everyone who is there was being served these days they are saying you have to wake up in the early to catch the que

ZM: What time in the morning?

RES: Maybe around 4 they are some that I found wnhen arrived at were is called Tafara Satilitte clinic they were sleeping out side,sleeping like this women I arrived at 7 asking then why are you sleeping they said grandmother we came from Gazebo we came from where ,so we came to sleep here so that they take a limited number if they had said that that day they are taking 10 they are taking how many people 10

ZM: Hmm

ZM: Hmm

RES: Think of it all the women who are preganant they say they are taking 10 if you don’t catch that whole week for you to take a number amongst the 10 do you see it coming out you will finish the whole week without registering

ZM: Hmm

RES: Having done that you cannot find others you know them who will say for you to be served fast or for you to be helped quickly do me something then I will do you something so corruption will never end you understand

ZM: Hmmm

RES: It comes back again those women has given birth the injection that are injected to children BCG what what they are not there,certain medication is not there,come back then do what what,if you don’t have anyone you know or if you don’t have that for coke your baby might not be injected you understand

ZM: Hmm

RES: Now as we are speaking my daughter baby card that card that shows that this child was born at the clinic, is he a boy or a girl what we call baby cards they don’t have,if you find someone who has them they are asking for 4 dollars for you to get what…

ZM: To get a card

RES: To get a card you understand

ZM: Hmmm

RES: Back then just after given birth you would leave with your birth record,baby scale card if you are going for 10 days you would go and get help so see its now different

ZM: Hmm

RES: Back then we were told that our children less that 5 years they are supposed to be injected until they are 5 now they are limiting they are saying 1 year 6 months or 18 months that’s were they are ending their immunization but ours when we gave birth they were saying a child up to 5 years should be going to scale

ZM: But those injection are children getting the on time?

RES: They are not getting them that’s what I am saying that sometimes you can finish 2 months without getting that injection, telling you that you should come and vaccinate the child then you get there they will say there is no vaccine, if you want to come you will be told that we do not have electricity our medicine are not what what, we don’t have the syringes, there is no this theres no that you see

ZM: Hmm

RES: They will end up giving up,even to vaccinate the child they will not vaccinate they will leave it like that because she will be tired of coming back,she comes again then they say come back untils a person says I am not leaving it

ZM: Alright,coming to the issue of corona as a clinic what are the measures that the were implemented here at the clinic inorder to reduce the chances of getting infected by the virus or spread,we know that back then we were walking doing whatever we want going to get treatment and go back home,but because of corona a lot of people took measures to protect themselves as people,as a clinic or as a community, maybe we can start with you as a person are there any measures that you implemented in your life in trying to prevent yourself from getting infected by corona virus?

RES: Ehe cghekutanga ndakatoona kuti ndishande achipatara ndikuda maPP maprotective chii clothes, kubvira kumask kibvira gloves,kubvira zvese zvinodiwa kuti ndizvidzivirire uye zvekare ndinofanirwa kumaintainer social distance

RES: Yes first of all I saw that for me to work at the clinic I need PP protectivclothes, from mask from gloves, from apron, from everything that is needed to protect myself and also I must maintain social distance

ZM: Hmmm

RES: I am supposed to get sanitisers even when I am here at the clinic when walking in homes I must walk with my sanitizer showing that wherever I touch I sanitise then I maintain social distance whereever I am

ZM: Hmm

RES: Also at the clinic the same if a person is entering the gate they would want to know his/her reason of coming,they will be sanitise first whilst they are still at the gate and taken temperature to see how it is,

ZM: Hmm

RES: Even here at maternity they nolonger allow many vistors like what they used to do that is my friend has given birth the whole bed we will be surrounding her they are allowing one person per visit

ZM: Hmm

RES: Now if you are entering, long back they used to say when entering at the gate you would be accompanied by a car then leave you at the maternity door then you enter with your bag,now they are saying the one who is due fro the gate must carry her back alone and enter alone

ZM: Hmm

RES: I think you met when you arrived you saw people who were being discharged those waiting for her will wait outside the gate, they will be given the baby outside the gate

ZM: Alright so looking at at the issue of this disease we are not seeing that it is going to go soon, you as a health worker what do you think we must do looking at the situation that we have of COVID-19cause its not going

RES: I was encouraging that if its possible for everyone to get vaccinated to prevent this disease, we go through all these vaccinations that are being talked about,2 lets follow what is needed with COVID-19the whole regulations, if we were told to wear mask lets wear mask, if we are told to do social distance lets do social distance

ZM: Hmm

RES: I was hurt today as school children has opened,in a combi that I bordered children and parents are crowded there they are not wearing masks, I then said school children wear your masks are saying that you will wear mask when you have arrived at the school gate don’t you know that in this combi where you meeting with the parents thats were there is high risk you understand

ZM: Hmmm

RES: Meaning that from home the parents are not able to taking it serious that that children should protect themselves we just saying as long as the children are going to school,at school we don’t know if they are following what is required

ZM: Hmm

RES: Then it comes to us the elders if you walk round Kamunhu shopping center people has relaxed they don’t even have masks they are not maintaining social distancing these are some of the things that people should continue being taught

ZM: Hmm

RES: And people are like in the bible that they believe after seeing that someone died or that there is a problem for them to belive,right now If we walk shouting at the boreholes that social distance they will say haa excuse us who have you seen someone who died because they haven’t seen it happening

ZM: Hmm

RES: I don’t know they want to see many people who have died being buried four four like what was happening in other countriues so that they can belive that COVID-19is there, If we tell them that people died there are statitics that we have who have people who has died they will say that’s for yourself you looking for money,

ZM: Hmm

RES: You just want to get money there is nothing like that,if onlywe can remove that mind set in our living us personally I think that may help us, yes COVID-19is not going anytime soon but lets try by all means to protect ourselves

ZM: Hmm

ZM: Alright, going back to the servces pertaining PMTCT, looking at delivery that was done of PMTCT serives before COVID-19and now, can you say that there is a difference, is there anything that has been affected because of this disease of COVID-19looking at PMTCT services,from when the mother is pregnant until when the child is nolonger breastfeeding,has been removed on cotri has been tested,is there anywhere that has been affected because of this disease looking at our cascade of PMTCT

RES: There are things that have been affected a lot

ZM: Hmmm

RES: Because most of the children I can say starting from when a mother is pregnant some did not manage to register you understand

ZM: Hmmm

RES: They didn’t register,they did not register and the pregnant is due the fact that they didn’t register she don’t know her status the pregnant is due at the clinic ,this clinic reached a point where closed and this is the main hospital that deliver people here at Mabvuku poly,a person will then go to informal midwives those midwives they don’t know that person’s status,you don’t have glooves that will be be used you understand

ZM: Hmm

RES: They have no idea of the person’s status they will deliver that person she didn’t go on PMTCT, 2 also because of the clinic

ZM: This clinic closed for how long?

RES: It closed for 2 weeks

ZM: Alright

RES: Having said that, as many as people are this is the main hospital the beds are not enough they want to maintain COVID-19regulations they did not want many people there was a certain number of people that they wanted on a day the people they are delivering are like what, you would arrive and the grandgathers fail to understand that your pregnant is due

ZM: Hmm

RES: Then you say grandfather what… he will say no look for a car go with her to Mbuya Nehanda or go to Edith or to Chitungwiza, maybe the person doesn’t have money its the time of COVID-19that people are not allowed travel, maybe the person with the car doesn’t have enough papers they will say how will I get in town for me to go to Mbuya Nehanda many delivered at the gate

ZM: Hmm

RES: Someone at that area at New stands the person ended up giving birth in the well you undersatnd

ZM: Hmm

RES:Those are te problems that we encountered through the issue that people were not being accepted well those clinic we say the children have be born were they go to FSH its closed

ZM: To scale and what

RES: Ehe kutafara clinic vakasvika one time yekuti vakavhara, mabvuku satilite yakavhara hapana kana varikenda kunotora mushonga vana vacho, mushonga ewacho cotri chaiyo yakanga isingawanike yekuti mwana apihwe

RES: Yes at Tafara Clinic they reached a time were they closed, Red Bull,Mabvuku Satilites closed there was no one who was going to collect medication the children,the medication cotri was not there for a baby to be given

ZM: Hmm

ZM: Hmm

RES: Sometimes people would come to me then say that grandmother what should we do there Is no cotri, I couldn’t find what should I do with the child the baby is exposed he/she is on PMTCT

ZM: Hmm

RES: I will end up saying what can you do you have to buy, some even the money they will tell you that our job is of selling look right now we are not selling even on roads where will I get the money to buy cotri everything put the children on risk you understand

ZM: Hmm

RES: Someone went when they returned now there are other who we followed on defaulters and tested them some of the children were found that the child who was born natagive was found positive

ZM: What about looking at things like testing kits were they able to come to the clinics or there was a time they could not find these things and the medication in clinics

RES:There was a time that is was difficult and couldn’t be found,the resources reached a point that they were difficult ,that if a person wants to be tested today they will be told that they shoud come back

HM: Hmm

RES: That issue of being told to back there are times when a person has commited him/herself that he/she wants to do that thing at that time, you know if you have commited yourself that you want to be done that time if you fail to do that some will not come back they will say whatever then it ends like that

ZM: What about looking at children who were taken samples where they moving well to go to the Labs and what and what, were there any delays if we are looking at children who were done HIV testing children or mothers who were done HIV testing, there need for results after the mother has done viral load, on the transportation of samples was it affected

RES: On samples it was difficult because there are others who could come back twice or thrice and the results will still not be out. Me personally im not PMTCT but if I tell you that I was taken I now have a year from when COVID-19started there are no results for viral load that came out, I came back in January thinking that…they told me that we don’t have time to check because for us to do viral load we are chasing that we should give people medication you see

ZM: Hmm

RES: So its that were dfifficult

ZM: Alright, what about looking at the doctors, the grandmothers, nurses was there a time that they failed to come to the clinic because of transport issues, because of fearing that ah if I go there I don’t have adequate things I might get infected, did that happen looking at the side of the nurses

RES: They reached that time because you would try to… when you arrive then you say your pertition that grandmothers how are you doing things look at this they will tell that what can we do we are not enough, sometime sister incharge I arrived at one time at Tafara saying that I am alone at the clinic everyone is scared the nurses are not coming

ZM: Hmmm

RES: So I can’t book them all for them to register their preganancies or to do what or to inject children whilst I am alone will it come out

ZM: It will not come out

RES: It wont come out and that day when I arrived they were calling ambulance,at the ambulance they were saying at Parirenyatwa and Mbuya Nehanda they are not receiving,they wanted to know if the patient who is coming has a nurse who is accommpaning her for delivery to go there if there is no nurse who is accompanying her then no

ZM: Hmm

RES: The ambulance came and they said that if you don’t have that we are not carrying, you understand

ZM: Yeah looking at other disease like corona, other disease like those that came comparing, we had disease like cholera that troubled in Zimbabwe, we have the disease of Typhoid that troubled in Zimbabawe but looking at COVID-19comparing it with these disease and what was happening in the clinics during that time, can you say there is a big difference on what you encountered looking at covid, looking at other diseases that had happened before like cholera and typhoid

RES: There is a big different because looking at cholera and typhoid mostly we were working with our 2 hospitals infectious hospitals like Nazareth and Wilkings

ZM: Hmm

RES: People who got serious where going there but right now if you look everywhere most of the institutions ended up being COVID-19isolation centers,people being quarantined there,during COVID-19people were being quarantined they reached a time when borders were closed our realtives failed to come come here,we were afraid of visiting each other

ZM: Hmmm

RES: But on …what is it called

ZM: Cholera

RES: On cholera if you obseve the hygienic measures that were needed there was no prpblem, but COVID-19doesn’t have that, on your gathering you don’t know who has it or who doesn’t and you can infect many people be it at the funeral, be it at the party, be it where, schools ended up closing there was nohwhere you heard that schools are closed because of cholera, there was nowhere you heard that the schools are closed because covid..

ZM: Typhoid

RES: Because of typhoid, there are no churches that stopped praying because of typhoid this shows that this disease is deadly its different from others

ZM: We said many things that had happened in hospital,we want to look at problems that were encountered by mothers in homes and the issue of getting PMTCT services what else did the mothers face during the time we had lockdown the first one and when we had this one that we repeated what can you say are the problems that were encountered by the mothers?

RES: The problems that were faced by mothers are many my child

ZM: Hmm

RES: Countless times people were calling us to assist them to deliver their babies, but we also don’t know how to do it, so a lot ended up delivering on their own or with the help of traditional midwives. I know of a woman who reached a point of delivering by herself. The baby had a twisted umbilical cord and the baby died. She did not have the money to call an ambulance or even to look for someone to help, some of the traditional midwives were charging USD50.

ZM: Hmm

RES: She gave birth alone she coundnt find money to call the ambulance or even to look for someone, everyone was afraid, you will be alone in your house doing social distancing, locking yourself in your house there is no one who is coming in, and we have no idea that there is someone in that house or what is happening

ZM: Hmm

RES: Crying and what people said we don’t know what is happening there,until they heard that ah that’s what had happened,until she was carried like that going to the clinic but things will be hard, to the extent that you would lose your baby or the mother will end up dying during child birth because of that

ZM: She’s alone

RES: Yes

ZM: Can you say the mothers had enough information during lockdown that she knows how she can travel if they had a problem, can you say they had the information when the lockdown started the first one can you say the mothers who were preganant had information about that they are able to to go to the clinic what do I do on the first one

RES: There was no information, there were no awareness education they didn’t have, they were not aware people were told to stay at homes then it became stay at home there is no one who came….like me to walk around teaching people I was afraid, I was seated I didn’t manage to go to teach people at homes that you should do this and that, I was fearing for my life so there was no one who was teaching people

ZM: Hmmm

RES: Unless they had luck that they have found maybe on social media and reading those who have smart phones but how many have no one so things were hard

ZM: What about on traveling those who wanted to come lets say its hard were they aware of that I am supposed to have this and that for me to travel because back then people were not walking they were saying letters or what, so the mothers did they have them, the essentials that they can use for them to arrive

RES: They didn’t have and others didn’t even know what to do if they get sick, that my scale card is the one that I am supposed to show to show that I am going to scale or what

ZM: Hmm

RES: People were afraid of travelling they reached a point where by some who we were doing adherence support asking them why they are not going to collect your medication they said we don’t have letters to use when travelling.

ZM: Hmm

RES: Then I said your letter is your book that book is the one that talks when you get to the police, they would come back replying that the some of the police are rough if you give them your book to read they will tell you that I don’t know how to read

ZM: Hmm

RES: You know working with public that what it does those who are forced what, those who are frontlines some of them they have their stresses that they had during COVID-19to the extent that if you fail to communicate well you could fail to go where yuu are going the come back because you had failed you explain to them that I going for this and that

ZM: What about lets say you have managed to get to the clinic where they aware of the things that were expected, that if they arrived at the clinic that am supposed to to this inorder to reduce my cances of getting infected with covid,also reduce the chances of infecting others incase I have covid,people were they aware when we had the first lockdown that okay you have managed to walk to the clinic what are the things that you are supposed to do to reduce the chances of infecting others or for you not to get infected by corona virus

RES: It was little you would arrive or you arrive at the gate if there are municipal police they would tell you that mother you don’t have a mask you are not entering, get tested first or do this first that how they knew but all the way from home think of it you have come from as far as Mandara or you come from where is called Gazebo or you have come from where faraway then you arrive and returned at the gate because you don’t have a enough essentals

ZM: Alright what about looking at homes can you say that there is anything that troubled people at homes, can you say there is anything that troubled people at homes pertaining getting services maybe a person is staying with her husband maybe she didn’t tell him, at homes did they had issues that came because of the issue of covid, whilst people were locked down in homes looking at PMTCT services at home, the way people were living was it affected?

RES: Yes, it was affected a lot because if its PMTCT it’s encouraged that PMTCT is for the mother and the father its not for one person and you register together also do protected sex

ZM: Hmm

RES: Some were saying that few men were accepting that and telling him to go and register he will tell you that with this COVID-19where do you want us go ,also to tell him that that we should use condoms he will say I don’t have to buy condoms

ZM: Hmm

RES: At the clinics sometimes because there were condoms they were having unprotected sex, they are nolonger protecting so that they protect the child, father give me money to buy cotri for thye baby it has fininished they will tell you that where do I get it,

ZM: Hmm

RES: You are seeing that my market that I used to sell at Pakamunhu I am not selling because it’s closed so where do I get the money to buy cotri there was to much GBV in the homes because of covid

ZM: Alright, what about the children were they given medication well in homes because there was lockdown every one was just seated

RES: That’s what I was talking about at one moment it reached a point were others were saying grandmother so what do we do I don’t have money to buy cotri ,then If I find it then I will give them if I don’t theres nothing. Some will say the few that I got I will give her today then go for 2 days without givng so it to be able to finish a month so there was no adherence

ZM: What about looking at jobs at hom taking child care can you say there is something that has changed or that has been affected pertaining that there was corona people were in lockdown

RES: It was affected a lot the mother could have 5 children workload all the home chores are loking at her, all the children are waiting for you the new born baby wants also wants you,

ZM: Hmm

RES; I could leave then to play with the babay I am going to look for something to buy or what, Childrren because you were told that exclusive breastfeeding at six months the baby is not supposed to find whatever he/she finds

ZM: Hmmm

RES: Because the children you had left the baby with, a child is taking care of another child if the baby is troubling them or if the babay is crying you see he/she will be given water,youll see him/her be given porridge or given anything,because for the mother the family is now big ,children are not going to school,the father is not going to work everything is waiting for the mother, it was farming season maybe you want to rush to the fields to get pumpkin leaves or want to do to what fot the children to eat, you see

ZM: Yes

RES: You would see the baby given zapnax, the baby given freezits because the time for you mother to think that I should carry the baby to the fields you are fearing for his/her health so it was not possible, so you leave other children taking care the baby, the other childen might be 13 years or 14 years look after the baby, because he/she doesn’t want to be troubled the baby was being given food

ZM: Hmm

RES: Maybe the is on 6 months of exclusive breastfeeding

ZM: Alrigh what about looking on the issue of access to resources to use ,can you say COVID-19has affected people in their homes pertaining access to resources that they can have power and resources to get things like money,food and what what can you say there is something that has been affected

RES: It was affected a lot

ZM: Hmm

RES: Because the whole informal sector was nolonger there,some fathers stopped going to work or stopped to go to work, some of us our works its hand to mouth, they are vendors ,even to go buy goods in bulky at Mbare at the market it was not allowed,\

ZM: Hmm

RES: And if you were found without a letter or what you were beaten by police so most of them ended up seated it affected people a lot

ZM: Alright looking at the issue of different measures that were done that we entered into lockdown, they said youre nolonger able to travel, schools were closed, the borders were closed, what do you think are the impacts that it did on women in your community looking at closure of schools, closure of borders to say that you cannot travel, what did it affect on women in your area that you work in

RES: It affected that economically their business went down,ehh intimacy with the husband in homes became little you know spending the whole day looking at the husband 6 to 6 is not easy,and moreover the husband does not have anything that he is bringing at the end of the day,

ZM: Hmm

RES: Some ran away from their husbands and went to boyfriends because the husband was not able to provide something to give the children and then comes someone you can get something, many houses were broken some of the children were left be ophans some ran away from children children were left like that

ZM: Hmmm

RES: We have cases like that, that we ended up taking the children to social welfare, some we are looking for foester parents to take care of the children that have been left by their mothers, some the homes were broken they divorced in homes, some fathers were arrested trying to hustle for things to go on they went and left the families suffering

ZM: Hmm

RES: Some parents they were locked out side they didn’t manage to come back the mother and the father children were left child headed so it’s hard

ZM: Alright looking at these measures that people should not travel, closure of schools that we are supposed to do social isolation do you think looking at your community your catchment area is this feasible that you should stay in homes, do isolation,do social distancing are they feasible these measures looking at your community that you work with

RES: Looking at the community that I stay its not feasible, the houses that we stay are 3 rooms or 2 rooms some stay in 2 room the father get infected with COVID-19they said he supposed to go to isolation in that 1 room that’s the room that we cook in, we sleep in .we all eat in how do they isolate you understand

ZM: Hmmm

RES: The house you are staying you are renting, there are 3, 6, 4 lodgers you use 1 toilet everything the…they do together is there any isolation or qurantnine how are they going to do quarantine, at one point the other father at our next door ended up staying in the car after he was inftected with covid, he would sleep in the car there is nowhere to stay there is nowhere to sleep, he would spend he whole day locked in the car

ZM: Hmm

RES: Ahh the next door they are hearing that the father of that house has been infected with COVID-19and he is in the car, stigma and discrimination is now coming out hes being stigmatized they will be saying that why did they fail to build a house or to look for a big house now he is sleeping in a car,

ZM: Hmmm

RES: The one who is sick or the one with a sick patient how would you feel you see the impact that it gives,you wont feel free even getting fit you don’t get fit quickly at the end instead of dying with COVID-19you will die with stress

ZM: What about looking the issues of water, looking at how you get water can you say there are other things that were affected because of the issue that your catchment area doesn’t have the esssentials they don’t have what...

RES: Yes the time when COVID-19came that’s the time when water has not yet started raining,You always hear Mabvuku and Tafara has a reputation of not having water,the boreholes that we have are few,people fought and they were fighting for the boreholes

ZM: Hmm

RES: Fighting,pressuring at boreholes fighting that someone took someons position on the que they ended up saying what today this line is the one that is fetching water, if you look at the front there there is a bush that is there at New Stands there is a mother who woke up at 3 oclock with her child to go nad fetch water, the child was raped the mother took what she had then attacked that raper they he died at the spotpeople ran to see, peole killed each other,people were raped,people did GBV because of the issue of not having enough essentials

ZM: Hmm

RES: Water was being sold those who have the chance to get it they would tell that give me money be it 10 bond per bucket if you don’t have you would suffer so it affected a lot

ZM: Looking at all these problems that were brought by COVID-19our goal is that in future the services should keep on going,there should be no disruption like we said at the beginning,what do you think that is supposed to be done by the government to prepare that in future if we have another problem like COVID-19that it will not hurt people too much like what happened this year.Lets say 2 years down the line came another disease that is more serious than COVID-19we would want say we have learnt from other diseases that has happened before,what are the things that are supposed to be implemented be it in clinics,be it in community that services will keep on going well without disruption of services

RES: First of all prevention is better than cure, there is need for prevention measures first of all to prevent this disease before it even come not to prevent after the disease has already done what it did you understand

ZM: Hmmm

RES: 2. They must makesure that in the clinic the medication is there and it is enough that it will never have shortages it will be enough for everyone…if its posibble they must look for like what they were doing mobile clinics which were going on the center or at shops crowding giving people medication they must stay everything corrected like that

ZM: Hmmm

RES: Also if it is said that the Bauzers must go around the bauzers must go around giving people water, if its food we saw in South Africa they did grants that people were doing home delivery being given at each home especially the elderly and those with chronical illniess, they must be seen that those people have been consiredered by the government

ZM: Hmm

RES: Also again to frontline staff people who work who are at high risk they must be well protected not to hear that the doctor has passed or a person who works at the clinic was infected with covid,we have many cases some were infected by COVID-19who we work with here those things must always be protected

ZM: Hmm

RES: Without COVID-19only any crisis that will happen like floods you hear what they do, they must always know that the place it brings floods people must not stay on those places, not to wait or the floods to come then you say people leave go and stay to this certain place they must remove people on those places

ZM: Alright okay

RES:And they must look for help from other NGOs or other donors that when they get in other crisis people can get helped,if the people could give them humanitarians,give them food,givng then what they give them for people to get help

ZM: Alright, we talked about the issue of that they should stay well advanced right,what about on the issue of samples up today you are saying they are not moving well some haven’t gotten their results ,some maybe even being taken samples they haven’t taken ,what can be done with the government so that if a person was supposed to be tested at 9 months must be tested and get results,if the baby was supposed to be tested at six weeks then tested and get results

RES: That’s what I have said they must always be equipped if they see that they are supposed to employ a lot of people they must put a budget that if workers were 2/3 and there is a crisis there is a money that was put aside to pay other workers that will be doing this job

ZM: Hmm

RES: Alright there is one person here who comes with a motorbike to take samples or he is the one who comes then he fall sick there will be no one ot there is a spare, the death of Jonh company must not close there must be a spare who will come replacing the one who is absent things must keep on flowing you see

ZM: Hmm

RES: No that there is no one who is carrying your samples, there is no one who is doing that the one who is available is off or he has a sick person or he has bereaved they must always have people who will remain on standby, at schools they must see what they must do if there is a crising lke this how can children be able to learn

ZM: Alright thank you those are the queston that I had I don’t know if you have the questions you want to ask menow

RES: Hmm focusing on that you as researchers and the research that you are doing I think I saw where it was said you are going to review again

ZM: Yes we will come back again discussing with you seeing if there is a change

RES: Alright is it’s like that we would want to be told how our out come is

ZM: On that we will let you know we are encouraged that if the program has ended we are supposed to come back giving them results telling that it went like this, it might take a liitlee bit of time when the program has ended for people to gather all the information, the ideas and writing and what but coming back people will be coming back to tell them

RES: Also on that like I said we are volunteers we are willing if you have found other things that needs help ot to work with us we are here to give you information or help each other in the community if you ned anything we are here

ZM: Yes we thank, thank you foryour time
