# Supplementary material for: “Getting pregnant during COVID-19 was a big risk because getting help from the clinic was not easy”: COVID-19 experiences of women and healthcare providers in Harare, Zimbabwe
Source: PLOS Glob Public Health. 2024 Jan 8;4(1):e0002317. doi: 10.1371/journal.pgph.0002317 (PMC10773929; doi:10.1371/journal.pgph.0002317)
Supplement: S1 Data — (ZIP) [file pgph.0002317.s003.zip › Data/Health Promoter/Health Promoter 8.docx]

**Interviewee’s Gender: Female**

**Interviewee’s Age: Around**

**Interviewee’s Initials: HP 8**

**Length of Interview: 55.00**

HM: We are now starting, can you start by telling me your name, are you married, whats your highest level of education, also who do you stay with at your home that’s what you can start by telling me.

RES: My name is XXX, CCW ward 19, I stay at number XXX, in XXX location. I stay with my brother, my sister’s child, my sister’s younger child and my 2 grandchildren that who I stay with.

HM: Alright are you married?

RES: No, I am not married I'm single.

HM: All right you are single, what about school whats your highest level of education?

RES: At school, i learned from 1 grade up to7 at XXX primary school, form 1 to 4 I learned at XXX secondary school.

HM: All right, can you explain or tell me how you personally feel about the COVID-19 virus or Coronavirus, you personally how do you feel about this disease called corona virus?

RES: I personally to tell the truth about this disease called corona or COVID-19 it troubled me a lot and it also touched me because it just came suddenly and when it came it had no cure, it disturbed me a lot because many people perished. I lost many relatives, who we were not even expecting we just had they pass away because of COVID, and most of them we failed to go and mourn because there was a certain number that was required to attend funerals.

HM: Hmmm

RES: So, because of that I have never heard that there is a disease requiring, a certain number of people at funerals or you are required to do social distance when you go there, washing hands, and wearing masks.

HM: Hmmm

RES: I have never heard about that but because of corona it’s my first time to hear that there is a disease that comes in that way that if it takes a person or kills a person you are not allowed to go there or you are not allowed gather, you mourn and you pass condolences through phone calls and not attending the funeral.

HM: All right, is there anything else that you felt personally that COVID-19 did that affected?

RES: Personally COVID-19 affected me because other children were no longer going to school, so they were now misbeahaving. The, whole of last year children were not going to school, personally saying it affected me a lot because many children ended up getting into drugs and doing the wrong things because they did not have anything to do.

HM: Hmmm

RES: They could not go for extra lessons because of the lockdown restrictions andchildren ended up being astry.

HM: On drugs which drugs were they doing, do they do drugs here?

RES: Haa XXX community I don’t want to lie is the biggest home of drugs, in XXX they manufucture drugs soo much

HM: Which drugs?

RES: To the extent that we are asking that if there is a big organization that they will give to this community I think this community can improve because a grade 4 pupil is now on drugs.

HM: Where are they getting them?

RES: Right now, I can tell you that they make drugs, here they make ganja cakes, they mix concoctions and bake. They plant flowers and trees when you see them you think they are flowers or just a normal tree but in fact they are drugs and the children in this community are taking those drugs.

HM: Are there toxic drugs that are planted as flowers or trees?

RES: There are flowers that are planted in the yard but as parents, we will not be aware we will think that they have just planted a flower at home, or the children have planted their flowers, but those flowers and trees are being used as drugs.

HM: Hmm

RES: Also, right now we can see that many things have changed they have moved from what we call Mbanje (marijuana) they no longer see it as a drug, they are now seeing it as nothing. If they hear us saying marijuana tell you that you are still behind because they are other that are highly toxic than marijuana and we cannot talk about marijuana in this day and age

HM: So, what are they taking?

RES: Like right now they are taking...

HM: What is it called?

RES: They are taking pads, our pads that we use when we are on our periods us women, that’s what they are taking, and baby diapers that’s what they are taking as drugs.

HM: What do they do?

RES: On pads and baby diapers,they take the jell that is inside, They buy new diapers and boil them after boiling them there is a jell that is inside it will be sticky and thick that’s what they take and drink.

HM: Drinking?

RES: Yes

HM: Uhh is there anything else that they smoke, or they just do that?

RES: On smoking they are taking powder of….they take globes especially energy savers that’s what they are using they say especially the energy savers that had burned there is a powder that is there that’s what they are taking then they drink it

HM: Hmm

RES: Even if you they see TVs that had been thrown away or the thieves breaking in stealing TVs they are not stealing so that they can resale them but at the back of the TV there is a powder they are taking and drinking these children

HM: Alright

RES: And smoking

HM: Haa it’s hard, how has the COVID-19/corona virus affected your mental health and wellbeing, did COVID-19 affected your mental health the way you think or generally your health?

RES: On the way I think in my brain we were always troubled that alright if you think of it every day we were having sleepless night because everyday the numbers of people infected with COVID-19kept on rising everyday, so in my brain and thoughts you will be thinking that if the numbers keeps on rising tomorrow maybe its me or maybe tomorrow its my relative

HM: Hmm

RES: So becasuse it will be getting in your brain it kept on touching me that we are going to live like this until when without COVID-19medication that if you get infected if the medication is there It will be better you can get treatment, because if you get infected they were saying that there is no medication do this or do that so some of the things we were lying to each other so…

HM: Stress increases and depression

RES: Lowered down points, CD4 lowered because of that

HM: Because of that?

RES: Hmm

HM: What about at work can you please tell me your portfolio of work, your job what do you do?

RES: We are CCW standing for Child Case Worker we work with children in community the biggest thing that we do is identifying case and refer that what we do

HM: You identify a case and refer where?

RES: It depends on how the case stands

HM: You deal with which type of cases?

RES: Like for example we work with cases….the cases that we work with are of children without births if a child doesn’t have a birth we can refer here there are organisations that we work with, there are organisation that deal with children

HM: Hmm

RES: So if we identify a child without a birth we refere them to this other organisation called Mavambo, Mavambo is there to help that child without a birth they will ask you where was this child was born, if he/she was born here at XXX poly Mavambo gives us 5 dollars searching fee then after giving you 5 dolars searching fee they gives you $25 dollars for birth record

HM: Hmm

RES: Then you come here you get help they you go to Chizhanje to get a birth for your child

HM: Hmm

RES: Others that will be big we refer to JCT

HM: Like which ones?

RES: Sometimes if they say the mother and the father doesn’t have births they came from Mozambique but it’s the right of the child even if the father doesn’t have a birth and the child doesn’t have a birth but it’s a persons right to....

HM: To have a birth

RES: To have a birth so that they can be counted in the country so we will be referring to JCT

HM: JCT what’s that?

RES: Justice for Children Trust

HM: Okay

RES: Some of them we put them to department of Social Welfare

HM: What are they like?

RES: At the department of social welfare like a child doesn’t have……right now lets say a child doesn’t have a place to stay welfare comes back to us then they say lets go to the homestead and see how the child is living, if there is abuse the child might be removed then they look for a Home where he/she can go incase of safety.

HM: Hmmm

RES: Or if the child has been raped or if the person who had raped the child will be seeing her so the child will not be feeling free or if the person who raped her is seeing her and keeps on threatening the child to change statements so social welfare take the child and look for a place of safety as they will be fixing his/her case

HM: Alright, So now when you are in lockdown if thee anthing that has changed on your form of form?

RES: Yeah a lot we were now doing online work so some of the things were not coming out well,its different from that when you will go were the case had happened and take the child then you bring him/her to Mavambo then you will be given transport fare then you go to Social welfare.

HM: Hmm

RES: So on travelling we didn’t have the letters so that if there is a case we can travel with the case it was not possible because we did not have travelling letters ,so some of the cases they ended on the ground because there was nothing we could do,

HM: Hmm

RES: For you to leavehere with a case to XXX police you would meet police along the way then they will ask where we are going if you try to explain sometimes they don’t understand that you work with children not what you do in the communit they will say go back so the case will end like that

HM: Alright, let’s look at the health context in Zimbabawe about the health status in Zimbabawe can you describe the health care context that you work in, health status in your community that you work in how is it?

RES: In the community the sickness is there people are sick in their homes, some are walking sick, some are lying in their homes sick, some are crying because of the issue of hunger but like I said in XXX Tafara we have organisations that we work with, like for those lying sick patients we have Mavambo what we are calling Mavambo trust if helps a person to get medication

HM: Hmm

RES: There is also Mashambanzou Trust if the parient is HIV positive or has cancer Mashambanzou Trust comes at take the patient they go with them at their clinic they keep them treating them until they are okay then come back. Ireland Hospice again with cancer patients it comes and take the patient like that like that

HM: So during lockdown were people able to access these treatment services you were talking about?

RES: Haa during lockdown it wasn’t happening like that because we lost a lot of people because Mashambanzou wasn’t able to drive to come here even for a person leave home to come to the clinic here they were saying the clinic is closed or we are not working

HM: The clinic was closed during COVID?

RES: Yes I was not even wotking

HM: So in this community people were getting help where?

RES: They were told to go to private doctors

HM: What about those who does not afford what were they doing?

RES: They were dying in their homes

HM: Alright, what measures or changes are you making in response to COVID-19 or corona virus,you personally what measues or changes are you doing in response to COVID-19 or corona virus let’s start with you then we will look at the organisation then you community?

RES: Me personally I now working with what is there like are doing social distance mask up, sanitizer that’s what I am always doing

HM: That’s what you are doing even at home?

RES: Even at home, even in the community we will walking we are always putting awareness that mask up, sanitizer and social distance

HM: Alright, what about the organisation that you work who said they want CCW at organisational level what measures did they implemented in response to COVID-19

RES: The changes they made are that since people were complaining about hunger that there is hunger Mavambo trust started giving children porridge every month so that children can get something to help themselves with at home

HM: Hmm

RES: Then welfare was sending money on phones to those who are 65 and above so that they can be able to help themselves, but its not everyone who was able to get the chance to enter in the department of social welfare even in the community its not every child who was able to go to Mavambo Trust to get help with porridge.

HM: Hmmm

RES: So most of them were loitering around the story that was there, and being given jobs that children were doing child labour that the mother can go this side children the other side hustling so that they can to be able to survive but its not every child who was able to get in the program of prorridge

HM: What about measures that were done prevent ….looking at COVID-19 with your organisation

RES: The measures that were done are that they said now we are closing the offices and also that peole must not gather at the offices because there was no way they can open offices when peole were being retuned home so they said for now we are taking the measures of closing the office then we do online

HM: Hmm

RES: But sanitisers people were given, masks people were given they said you should stop travelling stay at one place, people must stay at their homes if the disease or the pandemic stabilizes then we continue

HM: How are you coping with these measures and the changes at home and at work?

RES: Ahh that’s what is there we are used we are working with that

HM: Hoo you are used?

RES: Haa its now okay we are working with that

HM: From your perspective how are health workers care workers perceiving the situation, your perception or your thoughts how do you think health care workers are seeing the situation that has been brought by COVID-19?

RES: Hmm for health workers it’s bad

HM: What is happening?

RES: Because those are people who are at fore front everyone comes to them so people like them they need a lot of help, they should get first preference and also for them to get things that protect them then others will follow but they must start with health workers

HM: Healty care workers

RES: Hmm

HM: Alright how has the COVID-19/corona virus impacted the delivery of PMTCT that we have talked about of pregnant women, how has corona virus imPacted delivery of PMTCT services in this community?

RES: On delivery it was very difficult because if a person would want to come to the clinic they were saying that the clinic is closed and the pregnant would be due for her to go somewhere…..sometimes they were told to go to Edith you will need transport, when you arrive at Edit they will say they are fully booked

HM: Alright

RES: If they say it’s fully booked, the person is coming back thats when people were giving birth at their homes because during COVID-19I don’t want to lie home midvives (informal midvives) increased they came out home midwives

HM: Hmm

RES: But to these home midvives (informal midwives) they did not have protective clothes although they were delivering but protective clothes most of them didn’t have. Most people were giving birth where…

HM: In homes

RES: In homes some gave birth in the road they couldn’t find a place because here they would be told that it’s full, they go to doctors 24 hour they cant afford the money they will be told

HM: They can’t afford

RES: I have someone who gave birth in a well

HM: In a well what had happened?

RES: They said her pregnant was due at night she just went out she wasn’t registered so she arrived at the well next door she entered there she gave birth to her child there

HM: Was there no water in this well?

RES: In October our water level will be low

HM: Is the well shallow it’s not deep?

RES: It’s deep

HM: How did she get in?

RES: She entered straight then stand pushed and gave birth to the baby and took the baby into her hands, so those at the next door who were sweeping they heard the baby crying plus they also seen blood going to the well, they were helped by another boy from next door who took his car then they came to the clinic and they get helped

HM: Alight, What about those who had preganancies for the first time, first pregnancies needs to be registered then get tested how were they doing it for them to know their statuses because we know that most people don’t go for tests to know their statuses they wait until they are pregnant they say if get preganant will see it there at the clinic

RES: Others were saying that lets say you had registered your preganancy then when its due because they were nolonger doing scale, scale was nolonger done

HM: People were nolonger coming for scale to get their KGs checked?

RES: People were nolonger coming so even if the pregnant is due they were returned back with the municipal police,they were told to go back by the grandpas municipal police, if a person arrives at the gate they were told to go back because the clinic is closed.

HM: Hmm

RES: People were being received by the municipal police they were the ones who were doing the job of returning people because they were the ones who were at the gate. They will be people here who were not knowining who were knowing that there people at the gate but the municipal police were receiving them and say go back the clinic is closed

HM: What about those who were pregnant who wants to register for the first time fot them to know their status?

RES: Haa it didn’t happen during COVID-19t hat’s why you are seeing that there a lot of people who are pregnant they are coming after COVID

HM: What about those who wanted to register that time?

RES: No one registered

HM: So it means that there are people who gave birth without being tested to know how their status is.

RES: Because when they came here to register their pregnancies who were going to register to .The grandpas at the gate were saying that the clinic is closed, how were they going to enter after being told that the clinic is closed there is no way you could jump in to get in

HM: No way

RES: It was not possible and they were going back after you have arrived at the gate tomorrow they say go back again you don’t come back again, because a person will be doing their job you cannot go forward

HM: What about those you were already knowing their statuses were they able to come and get their medication those who are on ART?

RES: Who are on ART?

HM: Yes

RES: No those who are on ART their card enabled them to travel freely to collect their medication

HM: So there was no a time they didn’t come collect their medication saying that they failed because of COVID-19or because of lockdown

RES: Because when you…. from what I saw everyone who was on ART had their cards even if you meet the police or soildiers if they ask you where you were going you could just show them your card or even here at the clinic you were given your medication what was not there is contri, they don’t have cotri up to now

HM: They don’t have cotrimoxazole?

RES: They don’t have but

HM: Other pills are there?

RES: They are there you would come and collect 2 minutes then go back home people were not allowed to gather, but medication people were getting it even if the person is a visitor he/she was getting it

HM: If he/she has a card?

RES: You could come and tell them that I am a visitor, yes

HM: So those whon were suffering the most are new clients those who wanted to how their status is, but those who already knew their status were being helped

RES: Those who already know their status they were being review date and they were getting their medication I didn’t see anyone who go back saying I went to the clinic and its closed we were returned home no,everyone who was on Art was getting their medication,the problem was on the women who were pregnant they were on hard time because haa….

HM: Others ended up giving birth...

RES: They would arrive here and told to go to a certain midwife that…..and there was someone who gave birth at the gate so ahh preganant people were having a hard time

HM: Alright, what about those who were taken blood was it being trasported well going for testes, the blood samples were they transported on time what was happening or they were saying the clinic is closed?

RES: On that people are being taken viral load what what hmmm theres no one who was taken viral load during COVID-19ahh theres no one they started taking viral load in January or February

HM: This year? But the whole of last year no one...

RES: Those who were taken were taken before COVID-19inbetween ahhh viral load

HM: What about health workers were they coming to work?

RES: Yes but…

HM: The nurses?

RES: Yes they were coming but…

HM: In fullscale or they were coming in few numbers or you could count 123 are for this week then 123 are for next week

RES: Yes they were saying these ones are on duty this week because it was said that there are nurses and grandpas and grandmothers who had tested positive for COVID-19at this clinic, so that’s what made them close it

HM: Okay

RES: So I think they were giving each other duties that if these go this week the other ones goes next week they were no longer coming in numbers like what they were doing at first they started to come in numbers after COVID

HM: Were they afraid, were people afraid even the nurses were they afraid of getting infected with COVID-19?

RES: Yes they are people like us they were fearing for their health since there was no medication to prevent COVID

HM: Alright

RES: So they were fearing for their health

HM: Even people were they not afraid if they got sick they stay at home saying that if they go to the clinic they can get infected by this corona

RES: People were not even going to the clinic, people were afraid to come of which they were lying to each other that 50 nurses has been infected at the clinic, there was a rumour that spread saying that grandfather who has died, because we are in a comminty it reaches the commiunity with a lot of punch so even when going to the shops people were avoiding passing through what…

HM: The clinic

RES: Clinic area saying that is was said that there is COVID-19so don’t pass through it so people were avoiding, so if you come here when you go back and say that I was at the clinic people would go far away from you saying you are the ones bringing us COVID-19from the clinic

HM: Alright because of COVID-19there was no PPE so that those mothers we were talking about who were pregnant could be able to come to the clinic,those mothers they didn’t have things to protect themselves even the nurses at the clinic they didn’t have proctective things what was happening?

RES: Haa on that the mothers were not even coming even the nurses were afraid that if we say they can come and we don’t have PPEs what we will do to them so it’s better for a person to….

HM: Stay at home

RES: To stay at home then we stay at ours so that we don’t mix haa work was not moving

HM: How does COVID-19 situation compared to experience of other disease outbreaks, you comparing COVID-19 and other disease that happened way back like cholera and typhoid what can you say COVID-19is like or other disease how were they like?

RES: Hmm eveer since I was born until now I have never heard about a disease like COVID-19because if you see a disease that shuts down the whole country that the whole world,people doesn’t go to work, people don’t get treatment, children don’t go to school if your relative passes away you don’t gather I cant even explain it because its scary

HM: So you are saying cholera and typhoid was much better

RES: By far it was much better because if you get cholera you would get treatment then told what to do 2 minutes but COVID-19killed a lot,no COVID-19killed a lot and if you look at cholera or typhoid when they came it didn’t kill like the way COVID-19did

HM: COVID-19

RES: Even when they said the person had cholera we would stay together in the same room treating and doing everything but on COVID-19they say put him in isolation do this do that so COVID-19destroyed a lot and also it brought a lot of things back

HM: In you your community did many people die because of this disease looking at cholera comparing it to cholera and typhoid?

RES: In the community that I stay in XXX Tafara a lot of people died because of COVID-19some we used to worship with we would hear through whatsapp that this person has passed its COVID,people don’t go to the funeral ,people don’t gather mourn on your phones,before you even finish reading that message you hear that grandfather from this section has passed,before you do anything you hear that back there there is a funeral they are saying its COVID-19

HM: Hmm

RES: Oer day you would hear that 4,5,6,7 people that you know has passed away because of COVID-19.Then you can’t find how this disease came like.Haa as for me it took a lot of people in the community that I stay it has left a burden and a big gap and it will take time to find someone to fill that gap

HM: All these deaths were they being recorded by the Ministry of Health were they aware of what was happening?

RES: Yes it’s the same with those first days when they were saying that if a person comes from South Africa there was a number that was there that you would report, lets say those you had jumped then they come home at night what what we had the number that we were using, we would come here most of the time to tell incharge that incharge in our area there is person who came from South Africa but they arrived at night even at the police we would tell them that there is a person who came from Zmabia with a truck but they came at night showing that they were quarantined they did come with the right way

HM: Hmm

RES: So because people who are coming from outside are some of the other people who were coming with this COVID-19disease plus the person travelled at night so why don’t you check, so the police and sister incharge helped a lot on doing follow us

HM: Describe the changes to your service provisions as response to COVID-19/corona viruds, where there any changes that happened that you would want to explain about to your service provison, the way you would get treatment programs or other services

RES: On services most them went down to the extent that we are starting to rise now but most of the things are down because people were getting help of different things but because eCOVID-19came and closed peoples jobs, closed people who were earning a living from what they were doing so right now most of the things are still down they haven’t picked yet

HM: Hmm

RES: So there is need for a lot of time, if only we could get a donor or wellwisher who can come and help us, they need a lot of help because many people lost their jobs plus many marriages were broken

HM: Why what was causing?

RES: GBV

HM: It was too much?

RES: GBV increased a lot to the extent that most people…we can say lets walk around and here this mother and her husband separated, here this mother was found fighting with her husband.The stories are too many that’s what I was talking about that children ended up doing drugs some doing prostitution others if they are drunk they ended up rotating each other, many children got sick of STIs under age children

HM: During this period

RES: Hmm

HM: Underage from what age?

RES: 16 years 15 years I have cases that I know that the child of so and so got sick of so and so sexually transmitted disease but they did not manage to come to the clinic most them treated each other taking traditional medines but to say that they came to the clinic its a lie they didn’t come

HM: They did not come were they scared of COVID-19or they were scared of being known?

RES: They were scared of being known also afraid of COVID-19also they did not have the money to pay at the clinic to stamp their cards

HM: How much is it to stamp a card?

RES: Right now its 5 USD without medication just stamping that I have arrived here

HM: 5USD?

RES: Hmmmm

HM: What challenges do you think mothers were encountering in trying to access PMTCT sercives during lockdown, what cahellnges were being ecountered by mothers in trying to acess PMTCT services of mother to child transmission?

RES: They did not manage to get because most of them they wanted to get PMTCT but there was no where to find it

HM: The services?

RES: There was nowhere they could find the services they were nowhere to be found,because you would arrive here they will say its closed they you go to Tafara at FHS when you arrive the grandfathers will tell you that the clinic is closed ,then you say let me go with the child to baby clinic the grandfathers will say that the clinic is closed so many people stayed at home they did not get any services even 1 other gave birth whilst they are in dark and they were giving birth in homes

HM: Alright do you think the patients in your community get….have enough information during lockdown about where they can get PMTCT services even if I am in lockdown?

RES: Haa they did not have

HM: They did not know that during lockdown I can go to the clinic to get help?

RES: When they heard that the clinic is closed they didn’t bother to look for other people who can help them in the community or near them or anyone who knew about PMTCT they didn’t, they just said since the clinic is closed we are not travelling there is COVID-19so its better for us to stay at home when things are fine then we start from there

HM: The clinic closed closed for a long time or it closed for that time when they said that there are nurses who tested positive?

RES: From when they started to say lockdown some of the services were down

HM: They had closed some departments?

RES: Yes they were closed

HM: Which departrment was left working, Art for those who take ART was working that’s the pharmacy and where else?

RES: Phamacy no you were collecting your pills at….

HM: One place were you get your card stamped?

RES: Because thay did want people to go at the back there at the pharmacy after stamping your cards with the grandmothers, the pharmacy was closed the medication was staying with the grandmothers

HM: Hoo so they were given there and there?

RES: Everything there

HM: That’s all that was left working what about materning was it closed?

RES: Maternity ughhh

HM: What was happening that made you sigh

RES: Maternity was closed and open

HM: They were attending emergency cases only or what?

RES: Yes if people arrive at the gate, at the gate there will be the grandfathers the midwives will be here so we don’t knw if the midwives are the ones who had told the grandfathers (municipal police) that the clinic is closed or the municipal police didn’t want the patients to get in the clinic so they were stopped where….

HM: At the gate

RES: At the gate the midwives will be and not aware tha…

HM: People are being stoped at the gate

RES: They are being stopped at the gate, they were saying go to Edith or a certain house number in Mutate there is a midvives that delivers , go to Tafara in 50 theres a midwives you delivers but you had paid you money to…

HM: To get services

RES: Yes but most of them that money didn’t work because the municipal police was saying the clinic is closed go to….they were referring them

HM: Alright, Were they aware about that when they want to go to the clinic about the travel reguirements for them to to travel to the clinic what you are supposed to have when you are sick these pregnant women that we are talking about?

RES: Preganant women were travelling with their cards

HM: So why were they not allowd to enter when they had all their things?

RES: They had their cards they will be holdiong them in their hands but they were told that you see here we don’t have protective clothes so theres is no one who can receive you to help you because we don’t have the essentials, I think for them to say the clinic is not working its because of the essentials that they can protect themselves there were not yet there

HM: Hmm

RES: So they were afraid that if they ….these were they are coming from and the nurses had came from where they come from, the teperatures and what what were not yet being taken so I think that they were afraid that maybe they had come with it if it mix maybe theres is baby who needs to be touched after giving birth it will infect the babay, so the protective clothes were not yet there

HM: Alright those who were allowed to enter did they know how to follow the rules that were said, what they must do when they arrive here at the clinic?

RES: No haa social distance at the clinic was there even upto now its still there

HM: What other things were they required to do after they had come to the clinic?

RES: Sanitising, mask up that’s the rule that is there even now, if you have not been taken temperature you don’t get in the clinic if they see that your temperature is high they will…..they check your temperature when you are still outside the gate if its high they will tell you to go back if you have came to collect medication they will tell you to give someone your book and collect for you

HM: Alright have you noticed any changes in the number of patients seeking care on the onset of COVID-19, did the number of people coming to seek medication or treatment go down since the beginning of corona virus, did the numbers remained the same or they went down?

RES: The numbers of people were coming to get treatment?

HM: Hmm

RES: The numbers increased people wanted to get treatment but there was nothing they could do sometimes like what I said that the grandmothers didn’t have enough essentials

HM: Hmm

RES: Also because they were exchanging days of coming so those who would have come that day maybe 1 or two they were not able to finish attending to all the people because they will be tired. They will be tired and also they were afraid that we don’t have enough essentials you have you came from where ever you are coming from we don’t know if you have sanitized or what did you do for us to keep on helping many people we are fearing for our health and your health

HM: Alright, so the PPE that are being said that they were donated to the clinic were they not given?

RES: Right now its there

HM: Back then during the first lockdown were they protecting themselves or they didn’t have the esssentials they were not available?

RES: Because they were complaining about the issue of PP that we don’t have enough especially at the maternity, when delivering the baby that’s were they were saying we don’t have enough essentaials

HM: Alright,we are now talking about social issues at home in your opion how do you think the following factors affected women’s access and utilization of PMTCT HIV status disclosure, did it affect women a lot in accessing PMTCT services, lets say she came here and was told her results for her to be able to tell others

RES: Haa disclosure it wasn’t coming out they were not telling them

HM: What was happening that made people not to disclose their status?

RES: Some people they alaready have GBV in their homes so for her to tell her husband the results….

HM: Hmm

RES: It wasn’t coming out because there would need those counselling sessions fot it to come out as it is but here the counsellors were not there so for a person to disclose at home….haa most men were not accepting it.

HM: Hmmm

RES: So they were saying its better not to say it and do my things alone when things are stable and when the counsellors are there then i will tell my partner if we need counselling then we go for counselling because the counsellors were not coming

HM: So women were they the only ones who were coming for tests isn’t that when registering pregnancy they are nolonger doing that they come together?

RES: Ahh some men refuse out of 10 who come to register their preginancies only 2 came with their husband 8 they dont have

HM: Alright

RES: Plus some they will be telling you that I was impregnanted so I don’t have the husband some will say im from the bar I don’t even know the owner so what I want is to be tested so that I can register my preganancy and know my status so out of 8 women you came to register 2 have husband 8 doesn’t have

HM: Alright what about chilcare roles and responsibilitie sat home during COVID-19how were they doing it, how were children being treated, how was their theyre responsibilities and care?

RES: Hmm rights and responsibilities most of them were broken because the truth that is there is that COVID-19brought suffering and problems so the big issue that was there was child labour

HM: What jobs were they doing the issue of child labour is a hot issue?

RES: It’s the same as where I stay in this community that I stay you would see a child at 6 oclock they will….a grade 2 child they will be up carrying a sack those 50Kgs sacks they go at the bars in the morning to pickup the bottles of super, what are those super bottles for they wil say that they are for cooking at home and others are for sale

HM: To burn and cook that was their source of fire?

RES: And going where waste is dumped, the child doesn’t have gloves, the child is not wearing shoes, the child doesn’t have a mask they go around Mbavuku looking for waste dumping areas long fot steels if they fill a sack they come at Kamunhu they put then on a cage sell them and get money to buy vegutables

HM: Children were looking for money to support the family?

RES: Right thats what I was saying that most marriages were broken, the mother would go out to hustle on the other side the children will go out to hustle or the mother would go out leaving 4 children at home, after leaving 4 children the eldest will be 10 years or 9 years behind her there are other 3 they want to eat, these children were walking in peoples house asking for help soo

HM: It was hard

RES: People were taking advandage of that they will say come and remove weed in my yard for you to drink tea, come and weed here so that we can give you this and that so many childen they were being hurt with childlabour too much, and the children you saw that they were not going to school that’s what I am saying that those are the children who were doing drugs,they were spending their day in tuckshops,children were spending theis days in trenches they didnt have anything to do,some were starting to smoke

HM: Alcoholics

RES: Yes they didn’t have anything to do

HM: Alright what about on access and contro of resources how was it in the families women and men what was happening?

RES: To men and women it was GBV after GBV

HM: Fighting for money…?

RES: No we are used to that the father wakes up the law that the father wakes up and go to work then he comes back at night,now the bars were closed there is nowhere to spend their days,so the fact that we were spending the day together when we say we nolonger have relish,cooking oil has finished up the father will say if you are saying that cooking oil had finished up when you know that im not going to work so where do you want me to find it and the mother will say where do you want me to find it, so on failing to talk to each other they wil end up fighting

HM: There was no communication

RES: Haa communication was down

HM: What about on decision making powers at both household level at community level how was it going like during the lockdown?

RES: They were doing one men for himself

HM: Why are you saying so?

RES: It was one men for himself Because the wife could go out thny she says because you are not going to work youre just seated so even if she goes out and do what she does then she gets money when she comes with it she didn’t want to be asked where she got the money from or what did you do

HM: Hmm

RES: She will be saying its me who went out to look for so if you want to eat you can eat if you don’t want to eat dont eat because theyre mine I am the one who brought it you are just seated you are not doing anything so it was one man for himself

HM: That is what was happening in your community?

RES: In the community you would walk and hear….wherever you arrive its hunger

HM: Its hunger only

RES: It was all about hunger and funeral that has filled the community

HM: Alright, government of Zimbabwe has implemented the major challenges and social challenges including social isolation that people should stay in their homes, don’t go to other peoples areas people should stay in their homes, people are nolonger allowed to walk around,closure of schools,closure of borders how did that impacted women who stay in your area

RES: That people were nolonger travelling the borders has been closed I saw that it was good but on the other hand it was bad. Lets say the good part was that we wanted to reduce the COVID-19pandemic but it was bad because other women who were crossborders they are mothers and fathers,they were crossing borders to buy and sell for the family to survice so their bussiness went down and we were seeing them doing prostitution

HM: Hmm

RES: Because if you go around the bar even if they said curfew you would find 1 or 2 walkig around the bar looking for men .so women were having hard time, men would spent their day at the bar being chased by the police and soildiers whilst women were the ones who had problems because they were the ones who would see what the children had eaten and how are they surviving men didn’t have time fot that what they wanted was to be served sadza

HM: Do you think these measures were helpful especially in your catchment area in your community do you think these measures are feasible especially in your catchment area. These restrictions that are being said of social isolation, travel restrictions in your catchment area closure of schools, closre of borders was it helpful

RES: I saw like it helped a lot because we were telling children to stay at home,don’t travel, children wash your hands if you go out to look for vegetables wash your hands,wear your masks correctly,it helped because it reduced the COVID-19 pandemic even though it had spread.

HM: Hmm

RES: The law made it easier the law said they don’t want to see people gathering it’s a crime that you would have committed, you were not entering the shop without a mask, without sanitizing, without taken temperature so it helped a lot

HM: Even up to now it’s still helpful?

RES: Even now it’s still helpful because there is nowhere you can enter a shop without a mask, theres nowhere you can go without a mask. Even at school it’s still helpful because without that we were going to infect each other them we go back to level one

HM: What measures or programs are needed to mitigate negative impact of corona virus in your community, what measures or what programs can be done to reduce the nagative impacts that where done by COVID-19 /corona virus in XXX

RES: If we do workshops

HM: Hmm

RES: Then we include Lets say half men and half women then do workshops even if we do for a month and doing community works and awareness campaigns

HM: What does the workshops helps?

RES: Because if we are at home we will telling each other lies that there is no COVID-19but if we meet and put our heads together and enlighten each other we willcome out with a one thing that is tangible that COVID-19is there and its there

HM: Hmm

RES: Then we do raod shows for someone who didnt come to the workshop but if we walk with a van coming from lets say Chizhanje then Highstreet,Matongo, them Kamunhu, then Old Tafara then Gazebo we come back doing roadshows teaching people with VFU then we walk with Women Affairs it will come out correctly

HM: Hmm

RES: Then we be will pushing VFU pushing their word with Women Affairs pushing with other different organisation, pushing the word that we must stay in homes, lets do social distancing, why is COVID-19bad, why is GBV bad ,why is child labour bad we will be talking together that this this organisatin will be doing that the other organisation doing what they will be doing

HM: What do you think are some of the health impacts of COVID-19 incluing beyond the infections Itself, what you think that these are some of the health problems the we are going to encounter now or after the disease has ended, problems that we are going to see because of COVID-19because they can say it has ended but in the near future what are the the problems that you think we can encounter?

RES: Let’s say COVID-19has ended

HM: Hmm

RES: The problems that we can encounter are…

HM: Are there no problems that we are going to encounter?

RES: The problems will be there because our things for us to say that we have returned...

HM: To good health...

RES: To good health it is going to take time because some of the things that were being done before COVID-19came we are not getting them, for example the issue that we have discussed on medication that now we are supposed to take ART then take Cotri but now we are not getting it

HM: Hmmm

RES: Those are some of the things that happened and it brought us down because now we don’t have the money to go to the phamarcy to buy cotri we are taking Art like that we don’t have money to buy cotri so for us to say things have picked we now have money to buy cotri or we have found a donor who is going to buy cotri it’s a big issue so are saying the donor returned because of COVID

HM: So you are saying after COVID-19people are going to get sick because they are not getting enough medcation this will cause problems in the near future

RES: Lets say right now let it start here the vaccine has come they would have asked those that on medication is there is anything that needs to be added on the medication that we are taking some take on daily basis,when the vaccine came they could have said with those who are not getting pills at the clinics,the medication that is not found at the clinics that is needed everyday when the vaccine came they could have brought Cotri because some are on cotri and the child is on cotri but for you to buy cotri every month the father is not working things are not well

HM: What do you think are the socio economic impacts of COVID-19 short term and long term socio impact, socio we are talking about socialization that we do how is the impact,economic we are talking about finding money, job searching,how people are working what do you think are the socio and economic impacts of COVID-19 short term and long term impacts

RES: Haa on working I think thing are now better we entered into debts last year we were hustling like that but now things had gone back… they have not yet stable the haven’t reached on that level but at least they are now better

HM: Hmmm

RES: We should continue mantaning that the government had said, we should not gather, we should not work crowded, masking up and sanitizing now its better people are hustling, they have not yet reached that first stage we had before but at least they are not better

HM: How was it before corona?

RES: They were fine peole were hustling freely

HM: The problems were during corona?

RES: We were forbidden from travelling, working and doing the jobs that we were doing so there was nothing we could do we were just staying at homes

HM: What are the things that you recommend that if the country could do this it might help on corona or this pandemic?

RES: The country must give help to every one because everyone was in trouble.We can say every one encounted the problems of COVID-19if the country could help us by giving us 3 months of paying us,giving us something that every month they can say go to welfare and collect sack of mealie meal,cooking oil,matemba or beans or chunks,or say that for a year people should not be pay fees they learn for free,those going to the clinics they get treatment for free until our economy has boosted

HM: What else can be done as national response to COVID-19 that you recommend, that you can say they can do this as a national response to COVID-19 in the whole country?

RES: The whole country is complaining about hunger, as a country we need help and we want that vaccine to reach to every person every one must be vaccinated not that this one has not every one must be vaccinated so that we know that we all have prevented

HM: Alright, what mesures or programs can be put in place to help mitigate the negative impact yecorona virus within the work places within the community, what measures or what programs can be put in place to help to reduce the negative impacts of corona virus within workplaces,in the community that we stay

RES: Like I said at the begining they must do awareness campains and make fliers then do a lot of workshops it will come out

HM: The workshops will be done how you are afraid of COVID-19like this?

RES: We will be mainting the number that have been said 30 30 at gatherings if they take 30 today this area tomorrow they take 30 this area like that we all fit in and we can all learn

HM: Okay thank you with the replies you have given me, thank you for your time we have learned a lot that was happening in this community since it’s the first event we didn’t have experience on what are thing things that are encountered by people in this life through the lockdown that was there, I don’t know if you have any questions that you want to ask

RES: Haa no thank you being with you and time we were together having the conversation

HM: Alright thank you
