# Supplementary material for: “Getting pregnant during COVID-19 was a big risk because getting help from the clinic was not easy”: COVID-19 experiences of women and healthcare providers in Harare, Zimbabwe
Source: PLOS Glob Public Health. 2024 Jan 8;4(1):e0002317. doi: 10.1371/journal.pgph.0002317 (PMC10773929; doi:10.1371/journal.pgph.0002317)
Supplement: S1 Data — (ZIP) [file pgph.0002317.s003.zip › Data/Mothers/Participant 1.docx]

**Interviewee’s Gender: Female**

**Interviewee’s Age: Around 33 years**

**Interviewee’s Initials: Mother 1**

**Length of Interview: 28:26**

ZM: Alright maybe the first thing I want you to tell me is how old are you, where you stay, do you work, does your husband works how many people you stay with at your home.

RES: I’m XXX years old my husband doesn’t work

ZM: How many children?

RES: Altogether we are 6 with this one we are 7 (pointing at her child)

ZM: Are they all your children?

RES: Yes they are all my children

ZM: Okay

RES: 3 are mine 2 are my husband’s

ZM: All right, do you work?

RES: Hmm

ZM: Do you work?

RES: No I don’t work

ZM: How do you survive?

RES: We were being helped but now I can say I don’t work but I hustle

ZM: What is hustling how do you hustle?

RES: Sometimes if I get something and resell it on the market that’s how we have been surviving

ZM: You said you were being helped who was helping you?

RES: We were being helped by this…… what is it called, DCA but now we are doing everything on our own

ZM: What were they helping you with?

RES: With food and we were given money

ZM: Were they helping you only or they were helping many people?

RES: They were giving many people

ZM: What were you being given?

RES: We were given money on a card then we go and bought at the shops.

ZM: Hmm

RES: Like OK and Pick n Pay

ZM: Alright how much money were you being given?

RES: It was depending on how big is the family

ZM: Okay you looking at COVID-19 what do you know about it or what you have heard about coronavirus disease?

RES: it is said that it kills, you have to wear your mask always, wash of hands everywhere you go, must always wash your hands and sanitize

ZM: How did you feel when you heard that there is this disease?

RES: I felt touched and scared and frightened, I even cried alone at home

ZM: What was making you cry?

RES: Fear gripped me, after I heard the number of people who were dying in countries abroad such as Italy. I have never heard of people dying in substantial numbers like that before and I imagined what it was going to do in a poor country like Zimbabwe and my poor community and I got so scared to such an extent that I did not want anyone near me or my family members. I was afraid to go to the shops, or to the borehole or even the clinic, I feared going out of my house as I could see COVID-19 all over in the air in people’s hands. I did not want anyone coughing near me I can say I was so scared

ZM: Hmm

RES: Saying God why are people dying like this?

ZM: Where were you hearing it from?

RES: From the whatApp groups and newsaThat’s what touched me the most, I looked at my children, I thought that say I might die and leave my children.

ZM: All right okay is there anything that you have changed in your family in trying to prevent yourself from coronavirus disease, are there measures that you did or any changes that you did in your home in trying to reduce your chances of getting infected with coronavirus?

RES: Hmm

ZM: What did you do?

RES: I would instruct my children to wash their hands before entering the house when come back from playing with other children outside.

ZM: Hmm

RES: They wash their hands every time they go outside, they wash their hands with soap when they come back home.

ZM: Hmm

RES: Because sometimes I couldn’t afford money for sanitizer they would wash with water and washing powder.

ZM: Alright what about you as adults is here anything that you have changed besides children that go and play in trying to prevent yourself from the disease

RES: We would wear mask and staying in place at home and washing hands where ever we are most of the time

ZM: Looking at this disease

RES: And doing social distance if someone arrive and not greeting each other

ZM: Alright looking at the nurses here how are they perceiving the situation that we are in the time of coronavirus

RES: Hmm I can say during that time when it was too hard we were not coming here at the clinic, we would end at the road some who wanted to give birth most of they gave birth at homes it was closed, you would arrive and there is be guard who will say go back home

ZM: Hmm

RES: Wear your mask and go back home that what we were seeing mostly

ZM: That’s what you were seeing mostly

RES: Getting help ahhh we didn’t see anything we would hear that people are dying at the gate some had given birth at the gate some had given birth in the area

ZM: Alright I want you to explain to me from the time you were pregnant and the time you gave birth, that the you were pregnant were you able to come and register your pregnant on time that is expected

RES: Hmm I didn’t

ZM: Hmm what made you not come to register your pregnancy on time?

RES: It’s the issue of COVID-19

ZM: Hmm

RES: I was afraid saying maybe it’s closed because when I went that’s when I registered but time had gone

ZM: You registered your pregnant when it was on what?

RES: It was on 7

ZM: It was on 7 months?

RES: Hmm

ZM: Alright okay because you were saying if you go to the clinic might be closed

RES: Hmm

ZM: Alright after registering were you welcomed well when you arrive did you find the scale books there and everything when you came to register

RES: To register the books….first time when I arrived they were counting us I think they were taking 11 people the first day I couldn’t get then they said I should come back I think Wednesday or Friday that’s when I returned

ZM: Hmm

RES: That’s when I arrived early and enter then I register, they were taking few people so that they don’t crowd

ZM: When you were taken were you checked well by the nurses or you were no satisfied with how you were checked, were you checked or did they do everything that was supposed to be done to you as you know what is done when a person is checked her pregnancy

RES: It was happening

ZM: Hmm what was happening?

RES: Ah it wasn’t happening they would arrive and say you register and pay the money to register, then they will ask you how many months it has then you say, if it can’t be seen that there is a pregnant if it’s around 4 month they would say get tested I don’t know pregnant test

ZM: Hmm

RES: You go and buy and then come back and show them then tested

ZM: What about being laid on bed being checked, touched and what?

RES: Ahh that didn’t happen

ZM: What was happening?

RES: They were looking at the tummy only then they leave you

ZM: Alright okay when you gave birth to your child were you tested on time the baby to see if he/she has HIV the time you delivered him/her ,did you gave birth here at this clinic

RES: Yes

ZM: When you came were you welcomed well, was it open when you arrived

RES: It was open

ZM: Hmm

RES: I delivered by baby well but on testing I was touched when I get home that why the nurses did see that my child’s hand is like this isn’t that they are supposed to see all that they are webbed

ZM: How’s is the hand like?

RES: These fingers are webbed

ZM: Which fingers?

RES: These

ZM: There are webbed were?

RES: You can’t see here

ZM: Hoo there

RES: Yes so it touched me that why didn’t they check him/her because the nurses are supposed to see everything the problem that is on the baby that the baby has this, so those are things that touched me that why are they sacred of the disease or what

ZM: So when you get home what did you do?

RES: I saw it on the second day

ZM: Hmm

RES: Because I had stiches I was sewed so when I wanted to bath him/her all that I was able to do that I was in pain, so on the second day that’s when I say let me try and bath my baby and oil him/her and clothe him/her

ZM: Hmm

RES: That when I did this to him/her hands playing with him/her kissing him then I said ah that’s how my baby is they I called his father telling that do you know how the hand of the baby is his are webbed

ZM: Hmm

RES: Then he said ah I will come and see him/her It was in the afternoon he had gone somewhere that when he came and saw him/her

ZM: After seeing him/her what happened

RES: He said why is it the nurses didn’t see it then I said I don’t know maybe it’s because she/he still an infant so it can hardly be seen if they are holding like this, that’s what I said because he/she is an infant maybe they said the baby is fine when they did this to his fingers

ZM: Yes so did you come back to the nurses for him/her to be checked

RES: Yes I came back and they saw him/her

ZM: What did they say?

RES: They said sorry grandchild that what they said, then they said I am supposed to go to Parirenyatwa then I went when I returned I didn’t go to Pari I went to Harare hospital at Pari we could find a doctor

ZM: Hmm

RES: So we went to Harare hospital then they said I should come after 6 months so when they are due that’s when I am going with him/her so that he/she can be operated

ZM: So for now they said leave him like that

RES: Yes they said I should wait for 6 months when it’s due that’s when you come back then I get help

ZM: But is there anything that is disrupted on crawling or to do what looking at him/her because of....

RES: Haa there’s nothing but on touching I see that it’s affecting him/her to be free to be like this to touch everything since they are webbed the fingers can’t stretch

ZM: Alright but he/she doesn’t show pain or what

RES: On that

ZM: Yes

RES: No it doesn’t show

ZM: Okay what about your child when you gave birth to him/her was he/she given that medication that is given to children that are born with mother you are HIV positive

RES: Hmm

ZM: That nevirapine was he/she given

RES: Hmm

ZM: What about cotri moxazole when he/she reached 6 weeks

RES: Yes he/she is taking

ZM: Alright he/she is taking when your child reached 6 weeks was he able to get tested on time for you to know whether he/she has the virus or not

RES: Yes he/she was tested

ZM: He/she was tested/

RES: He/she was tested yes

ZM: Alright did his/her results came back on time

RES: The results of…

ZM: Of that test that was done when he/she was tested were you given the results there or they said you should come back and collect

RES: Hmm they said you should come back and collect

ZM: Yes

RES: That’s what they said

ZM: Did you collect?

RES: Ahh I didn’t

ZM: Why didn’t you come back and collect?

RES: Ahh I thought maybe they will call us

ZM: Hmm

RES: Because it was said that the clinic had closed so I haven’t gone to collect

ZM: So you don’t know that your child……

RES: What is it is it viral load?

ZM: At 6 week the baby it taken HIV test the baby on his own

RES: That’s the one they take on the leg

ZM: Yes that one

RES: That’s the one I haven’t seen the results

ZM: Alright where you being checked your viral load the whole time you were pregnant, have you ever been done viral load

RES: Hmm don’t remember well but there was a time I was taken viral load

ZM: Alright, looking at from when you delivered your baby here at the clinic is there anything that you couldn’t get that you think you were supposed to be getting here at the clinic, the services, and medication and what is there a time you couldn’t find

RES: When I was pregnant?

ZM: Uhm

RES: Hmm

ZM: There’s nothing?

RES: Hmm most of the time I know that they say if you are sick go and buy this at the pharmacy, go to the pharmacy and look for medication at the pharmacy sometimes here they don’t have

ZM: Alright you child right now is you said he/she has 5 months

RES: Hmm

ZM: Alright okay looking in the community that you stay in can you say, what are people thinking about this disease?

RES: People had anxiety they are afraid

ZM: What is causing anxiety?

RES: It tied a lot of people, surviving there are so many problems in homes, most of them are getting money to use there and there then it finishes so hunger you can feel the pain ,everyone can say many people

ZM: Alright, during the time you were pregnant to the time we entered into lockdown do you think you had enough information on how you would travel when you want to go to the clinic, that was the time that was said a person is not supposed to leave his/her house you must stay indoors did you have enough information that when you wanted to go to the clinic how would you travel or how do you do it?

RES: Hmmm there was no what we only knew was that you must travel with a mask wear a mask and always sanitize

ZM: Hmm

RES: Or when you arrived there you get sanitized

ZM: Alright did you know whether the clinic was open and also are they scaling did you know that

RES: Ahh we didn’t know we would go and returned there if you arrive there and they were not scaling

ZM:

ZM: What about how you would travel, are you allowed to travel when you are pregnant or after giving birth did you know of coming to 10m days 7 days did you know how you would do it during the time of coronavirusvirus

RES: Hmm I would hear…I didn’t travel that much when I was pregnant because we stay close to the clinic for scale

ZM: Hmm

RES: What I knew was that you have to travel with your scale book that can make you travel if you are pregnant

ZM: Alright did you know that time what is expected after you have arrived at the clinic,that if I arrive at the clinic what can I do to reduce my chances of getting infected with the virus from others and also not to transmit to others if I have, did you have the information that if you have arrived at the clinic how do you protect yourself so that you done get infected by other patients that are coming to the clinic did you have that information

RES: We were hearing that you are supposed to do social distance that’s what we were doing and not lean on everything

ZM: Hmm

RES: And washing hands and sanitize that’s what I knew

ZM: Alright looking at this disease it is said it brought many problems at homes because people were just seated not going to work, you looking at your family can you the coronavirusvirus disease has a problem that it has brought at your home?

RES: It has brought poverty at home and not being comfortable everyone at home

ZM: What made you not feel comfortable at home?

RES: We were scared if a person says am not feeling well here you would feel scared thinking that maybe he/she is going to die

ZM: Hmm

RES: That what we did most of the time and not knowing what to do for us to get money to work

ZM: Alright what about looking at the issues that some people has encountered problems on how to take their medication maybe the husband didn’t know, maybe a person was taking her medication whilst the husband wasn’t aware, did you have that problem in your house that don’t how to give the baby medication because his father is around or the father also had a problem of how he can take his medication because the wife doesn’t know he is on ART

RES: At my house I have never had that problem because we are all on ART for me to start taking it was my husband who said go I knew that my status that that’s who I am but I would feel so scared

ZM: Hmm

RES: Before I was taking but when I started taking we have never hide medication from each other we all knew that that’s how it is

ZM: You said you knew that that’s how it was what made you know that that’s how it was before

RES: I would hear some saying that if you have HIV you would feel pain everywhere, there are few days of being fit and many days you will be sick so I was afraid thinking that its true

ZM: Why would you think that it’s true?

RES: Because my husband was taking medication

ZM: He had told you?

RES: Yes

ZM: He came and get tested

RES: He went and get tested and he said my friend I have been given medication that’s how it is

ZM: Hmm

RES: Then I said you did well so when didn’t go he would scold me saying go and collect medication sometimes her would say tomorrow I am waking up with you we are going together

ZM: What made your husband get tested and you took time without getting tested?

RES: I would feel scared that my whole life I will be taking medication fear only that every day I will be talking medication and some were saying that most of the people who take medication their brains will not end up well so that’s what I was scared of

ZM: They said they will end up like what

RES: I don’t know how those people are always be shouting you don’t understand others, so that what I was scared of that I will end up like that haa let me not so I was afraid, I once collected at stopped taking the ended up I don’t know what happened to them I think I threw them away

ZM: Hmm

RES: I was scared if I look at them then I say ahh if they finish I will go again they finish I go back again and how big they are it was scaring me but when I took felt the change was there on me

ZM: Hmmm

RES: Ever since I started taking I have never got sick all along I was always sick lying down and what so the whole body changed it was changing But now it’s helping I don’t have pain anywhere

ZM: Alright looking at the issue of shortage of money cay you say your family has been affected by shortage of money because of coronavirusvirus disease

RES: Hmm yes because when schools opened my children haven’t gone to school they said they want books and uniforms but I haven’t go with them so I said they will go next maybe I would have hustled I don’t know

ZM: What about your husband’s are they at school or all they are not

RES: No one they are not at school

ZM: Hoo the ages they are still young children

RES: They are still young that they are starting the other one is supposed to go to ECD B he/she haven’t started going because of this issue

ZM: What about the other one you said your husband has 2?

RES: Yes they are 2 the other want to start going now the others ones are mine there’s no one who is going now

ZM: Alright do you stay with them your children?

RES: I stay with them all

ZM: Alright when we were in lockdown did you face any challenge of failing to take your medication well or failing to give your child medication well

RES: When what

ZM: When we were in lockdown did you face any challenge of failing to take your medication well

RES: Hmm I did have any challenge

ZM: What about of giving the baby cotri moxazole well

RES: Hmm I didn’t

ZM: You didn’t face any challenge

RES: Hmm

ZM: Alright do you think that the issue that when we were in lockdown the children were staying at home not going to school do you think it affected you mothers who are on ART for you to be able to travel and collect your medication well, because the children were no longer going to school they would spend the who day at home wanting to be taken care of, do you think that job of taking care of children did it affect the mothers on going to collect their medication or going to collect children’s medication at the clinics

RES: I don’t see how it affects because children’s medication everyone has always been collecting their medication there is no challenge that I saw

ZM: You have never seen any problem

RES: Hmm

ZM: Alright, what about the issue of that people didn’t have money to use and to travel to go and collect medication do you think it was affected because people didn’t have money to use

RES: Yes it was affecting

ZM: What was affecting?

RES: Person will not have money for her to travel to go and collect medication some collect from far away so for them to go and collect some were not going

ZM: Alright what about the issue that the fathers are the head of the house and they do all decision at home, they are the ones who gives you money even to use they do what, do you think this was affected because people were in lockdown the fathers were at home spending the day there do you think it affected anything on people for them to go and collect their medication

RES: Hmm because the fathers sometimes they didn’t have the money so it affected

ZM: Alright looking at you are someone who is always scared you said sometimes you would cry with fear of the disease you scared of taking ARVs is there anything you are fearing for on your child’s health that her/she is the baby that was born during the time of coronavirusvirus

RES: Haa now since he/she is getting medication so there’s nothing

ZM: There nothing you are always fearing for

RES: Hmm

ZM: What about you are you fearing for yourself is there anything you are fearing?

RES: What I am afraid of is that maybe they will close again maybe lockdown will start again that we will fail to travel again

ZM: Hmm

RES: And then we can’t find medication again we don’t know so that’s what scares me

ZM: Alright looking at the issue that the government implemented different laws in trying to reduce this disease like people should not travel, if a person get sick stay in isolation do you think it has affected you at your home these things that are expected by the government

RES: Hmm we were just afraid that a person Is said that he/she must stay on his/her own

ZM: Hmm

RES: Hmm it was scaring us that what if it happens in our house what will we do I would fee scared

ZM: But looking at your community do you think these things are doable that if a person got sick they can find a room that he/she can stay for 14 days and looking at your way of living can are people able to stay without crowding being separated is it something that can happen

RES: Hmm it can’t happen

ZM: Why do you say that?

RES: Maybe it’s the place I don’t know it cannot happen

ZM: What makes it not happen?

RES: If the people haven’t encountered the problem they will be thinking that maybe they’re jokes or lies, they will believe after seeing someone who got sick

ZM: If there in no one who got people will be thinking…

RES: They will be maybe saying maybe it’s just talking

ZM: Aright, looking at your way of living with the people in the community and how children play walking and doing what, is there anything that changed because we are living in the era of coronaviruslooking at the way you live with you neighbors, how your children live and children from other houses is there anything that changed because we are in the era of coronavirusvirus disease

RES: The change that happened to my children I told them to stay indoors at home

ZM: What about you and you neighbors and what, we know women has their friends who greet each other over the fence doing what

RES: Yes others we were seeing them walking on the road but as for me I was staying at with my children, that coming I don’t have many friends no one was coming to my house

ZM: No one was coming what about you were you going to others to kill time and plaiting hairs and do what

RES: Ahh no that’s when I started shaving my head

ZM: What made you shave your head?

RES: I was scared I was scared of this disease I said hmm for sure I used to plait my hair but on the pregnancy of my baby that when I started to see let me leave everything let me shave my head, my life I was a person who liked to plait hair but now ahh

ZM: Hmm

RES: I have friends who would plait me for free but now no I told them to plait those with money I’m no longer interested then I leave it

ZM: Looking at your community can you say people are afraid or touched because this disease of coronavirusvirus

RES: Yes people are afraid they are afraid people

ZM: Do they follow what it encouraged?

RES: Haa people don’t follow especially males females follow mostly

ZM: What makes man not follow?

RES: The man who drinks alcohol and smoke cigarettes that can make them leave home and look for it

ZM: Hmm

RES: So they will be crowding were they would have gone to look for cigarettes and alcohol

ZM: Does your husband drinks alcohol?

RES: Mine doesn’t drink but I was see it from his friends

ZM: Were they not coming to your house to spend time with your husband

RES: Haa my husband most of the time ahh they were not coming if he goes out that’s when I would scold him, even to go to snooker or what I would forbid him, but as a man you know he will find somewhere where he will go out

ZM: They will leave home saying I’m doing what

RES: He will leave quietly

ZM: What about when we are looking at the issues that many people were affected and failed to get services because of coronavirusvirus some mothers reached a point where they delivered in homes like we said, what can be done in future that if another disease like this comes people don’t get disrupted they must continue to get treatment on time

RES: What must be done is that maternity must not close were women deliver must not be closed at all

ZM: Hmm what can we do that they don’t close?

RES: Ah talk to the government I see that will work because if a person has reached a point of delivering they need help, you might die maybe you don’t have anyone to help you are alone so they must not close maternity

ZM: Alright thank you those are all the questions that I had I don’t know if there is anything that you would want to hear

RES: Anything

ZM: That you would want to ask me

RES: Hmm

ZM: There’s nothing
