# Supplementary material for: “Getting pregnant during COVID-19 was a big risk because getting help from the clinic was not easy”: COVID-19 experiences of women and healthcare providers in Harare, Zimbabwe
Source: PLOS Glob Public Health. 2024 Jan 8;4(1):e0002317. doi: 10.1371/journal.pgph.0002317 (PMC10773929; doi:10.1371/journal.pgph.0002317)
Supplement: S1 Data — (ZIP) [file pgph.0002317.s003.zip › Data/Mothers/Participant 10.docx]

**Interviewee’s Gender: Female**

**Interviewee’s Age: Around 34 years**

**Interviewee’s Initials: Mother_ _ _ _ _ _ _**

**Length of Interview: 27:19**

ZM: First I would want you to tell me how old you are, are you married, where you stay and what you do?

RES: I am XXX I am XXX years old.

ZM: Speak up louder so that my recorder can capture everything.

RES: I am XXX years old I am married; I stay here in XXX

ZM: Do you stay with your husband do you have other children at home?

RES: Yes, I stay with my husband I have 2 children I have at home.

ZM: This is one is the third one?

RES: This is the third one

ZM: Alright do you go to work?

RES: I do part-time

ZM: What part-time will you be doing?

RES: Of doing people's laundry or cleaning their house or going like now people are about to harvest I go to the fields to harvest

ZM: What about your husband what does he do?

RES: My husband what he does is that when he is called to repair cars that when he go and do that of repairing cars

ZM: All right can you tell me what you have heard about coronavirus or what you know about coronavirus?

RES: What I know is it’s there and it's killing people.

ZM: What kind of a disease is it?

RES: You have a high body temperature, sweating or feel cold, have headache, or have a cough.

ZM: Do you know the ways in which it can be transmitted, a person can protect him/herself?

RES: A person can protect themselves by washing hands, wearing a face mask

ZM: Hmm

RES: Yes, it can be transmitted when you are crowded at a place or in the community doing parties you can infect each other.

ZM: Looking at COVID-19 from when you started to hear about it up to now. How do you feel?

RES: When I heard about it thought that we were all going to die because it was said that it cannot be cured and there was no medication. I was afraid that if I got COVID-19 that was my end.

ZM: What about now how do you feel?

RES: Now I just do what has been said by the government to wash your hands, wear your mask always, don’t gather in crowded places.

ZM: Are you still afraid like you were when COVID-19 started.

RES: I still have fear, but not like it was at the beginning because I now know that if you wear your face mask if you stay clean, and do not go to crowded places you will not get infected.

ZM: Is there anything you have changed personally and, in your family, so that you try to prevent getting infected with the disease coronavirus?

RES: In the family when it started, we would say let’s follow the rules and regulations. We must not go out, we must not go to crowded places, let’s stay with our facemasks on, always washing our hands and 1-meter social distancing we were doing that so that it could help us.

ZM: All right is there anything that you changed in your household, to try and reduce coronavirus disease?

RES: Yes, I told my children I do not want them to go outside and play and, they should not go to parties or any place that is crowded.

ZM: Hmm

RES: They were always wearing their face mask and I told them not to go to the road.

ZM: Looking at healthcare workers how do you think they perceive this situation of COVID-19?

RES: Ah the nurses know that it’s there so they are encouraging people to protect their lives and they must not be in crowded places.

ZM: Hmm

RES: Even here at the clinic we are no longer crowding like we used to do back then.

ZM: What were you doing back then?

RES: Back then we would enter, and you will be told to sit close to each other but now they know that it’s there is COVID, so people are told to seat four people on a bench giving each other one meter distance in between

ZM: All right now we want to talk about you from the time you were pregnant to the time you delivered your child and breastfeeding. Focusing on the se PMTCT services. PMTCT services of trying to prevent transmission…..so that the baby can be prevented from being infected with HIV. So I want to hear that when you were pregnant were you able to come and get checked, scaled,?

RES: During the time I was pregnant I would come to scale and being scaled, we were scaled well

ZM: Hmm

RES: They would say stand there don’t enter in in many numbers were we will standing we observe 1 meter getting one at a time getting scaled

ZM: What about when we entered into national lockdown were you able to come for scale and getting scaled?

RES: Ahh it was difficult

ZM: What was happening during that time I want you to tell me about the first 2 weeks and the other that were added what was happening during that time

RES:

RES: Sometimes you would come and you will be told that there is no scale they didn’t want many people, they would say if you had come for scale go back home then we go back home

ZM: Alright, when you were pregnant were you able to be taken blood on time when you were supposed to be taken, lets start by registering your pregnancy did you manage to register your pregnant on time that is expected the time you were pregnant

RES: Ahh no

ZM: What did you do you registered your pregnancy when it was how old?

RES: It had 7

ZM: It had 7 months?

RES: Hmm

ZM: What made you register your pregnancy late?

RES: What made me delay was the issue of money so that I can come here to register it was the issue of money

ZM: When you came when your pregnancy was on 7 months did you find everything available were the nurses there?

RES: Yes they were there when I arrived I registered well

ZM: Okay from when you were pregnant up to when you delivered your child, was there anything that was supposed to be done to you as a mother that you say I failed to be done this at the clinic because of there was corona disease or we were in lockdown

RES: On the issue of baby card and BCG all this was never done to me

ZM: You have not, your baby was not injected?

RES: He /she was not injected

ZM: What did they say to you?

RES: They always say it’s not yet there, so when come they will be saying it’s not yet there come back

ZM: Hmm

RES: Then come back they will say it’s not yet there, the cards they are not there

ZM: Alright besides was your baby tested blood do you know that the baby is supposed to be tested…has your baby reached 6 weeks

RES: Yes he/she has reached six weeks

ZM: Was he/she tested blood to see is he/she has HIV or not, you the mother were you tested?

RES: Yes I’m on ART

ZM: You are on ART you were tested, you were on ART before you got pregnant or when did you know?

RES: I was already on ART

ZM: Yes when you got pregnant

RES: Yes

ZM: So you were taking your medication already

RES: Yes I was taking medication

ZM: So your baby was supposed to be tested at 6weeks was he/she tested yet has he/she reached 6 weeks

RES: Yes he/she has reached I went with him/her then they said come back for the results after a month

ZM: After a month?

RES: Yes

ZM: So you don’t know the results of the baby yet?

RES: When I delivered here they tested him/her then I came back for the results they said negative that’s when they told me that If I go with her/him to 6 weeks he/she is supposed to be tested that’s when he was tested and they said come back after a month

ZM: Okay so you haven’t collected the results

RES: I have not yet

ZM: But he/she was being tested on time?

RES: Yes

ZM: Alright what about medication when you gave birth here was your baby given medication that is given to children that are born from mother who are pregnant

RES: No he/she want given I went to buy

ZM: What did they say?

RES: They said they don’t have

ZM: Alright you went to buy but did you manage to get it, what about nevirapine that is given to the baby soon after being born was he/she given

RES: That the one I went to buy

ZM: Where did you buy nevirapine?

RES: They said at the pharmacy

ZM: Hoo so did you find it at the pharmacy

RES: Yes I bought

ZM: What about cotri moxazole was the baby put on cotri?

RES: Yes he/she was put when I went with him/her for 6 weeks so they gave me they gave me there

ZM: Alright you as a mother are you getting your medication on time are you coming to you review on time?

RES: Yes I am coming on time

ZM: Okay looking at the time you were in lockdown were you able to travel to come and collect your medication?

RES: Yes we were able to come

ZM: Okay what were you using in the road when they were saying they want letters they want what

RES: When you had your card for medication they were saying you can go

ZM: Alright, okay so all along you have been using this or there is somewhere you have been using

RES: Ah I started here that’s where I do everything

ZM: Okay looking at how you were accessing services during the time of corona can you say there is a different from how you were accessing services before we entered into the period of corona virus?

RES: Yes there is a difference

ZM: Where is the difference?

RES: The difference is on that on if you come like us who take medication if you come you are told to wait at the gate, so we wouldn’t want to be seen with many people but people are now knowing that those who will be standing there are for ART

ZM: Do you stand in different groups or you stand mixed with everyone?

RES: We stand but when they come to collect books they say that those of ART those who are on ART are the ones we are taking the people gave him/her books then they enter with them

ZM: Hmmm

RES: Then they will call you inside

ZM: So that has changed and it’s not making you happy?

RES: Yes

ZM: Back then what was happening?

RES: Back then they you said enter they we enter then we go you will be knowing that I’m going straight to where I am going, then you go and get help there that there was no one who would know that you going there

ZM: Alright, looking at the community that you live in can you say corona has affected people to fail to get treatment well, looking at other disease and people who are in the community can you say this disease if affecting people to getting treatment well

RES: Hmm I heard it was said its affecting those who are sick of TB and sugar it is said that it’s killing them so it affected people who are like that

ZM: What about looking at besides that corona is killing but the fact that there is corona do you think it may disrupt others from getting treatment from other clinic who are sick of be it with headaches or stomachaches do you think this disease making people fail to get different treatment services

RES: Yes its disrupting because it’s the same with this clinic here it might be closed then they say they caught someone who was infected with corona you will be home sick you can’t come to the clinic you will arrive at told that its closed

ZM: Hmm

RES: Some of us who wanted to deliver you were not able to come if its closed they will tell you that we are closed

ZM: Were you able to give birth here?

RES: Yes

ZM: Alright okay have ever been afraid that when my time is due I will arrive at the clinic and it will be closed

RES: That was I what scared of the most because the days I came here they had just opened saying that it was closed because they had caught someone who had corona

ZM: Alright the time you were pregnant when we were in corona until we entered into lockdown do you think you enough information pertaining where you can go to get help as a person who was pregnant if you want to go to scale, if you want to….did you have information on how you can travel during corona when people were not allowed to travel, did you have information on that, when you want to travel what do you use, how do you travel?

RES: I didn’t have enough what I knew was that you wear your mask then I walk going to where I am going so that I don’t argue with people on the road only

ZM: But did you know that you are allowed to go to the clinic if it’s open or what or your health checkups they don’t get disturbed did you have all that information when we entered into national lockdown

RES: Yes if you knew that the clinic is open they were helping

ZM: Aright

RES: You would come knowing that I am going to be helped

ZM: Did you know what you can use on travel if you meet up with soldiers or police because that was the time they were saying a person who can travel is the one with a letter only

RES: Yes a person with a letter

ZM: Hmm

RES: We would just walk we thought that they will be saying locals they will be wanting masks and when they ask where you were going then you tell them that you are going to the clinic

ZM: Alright, did you know how you were supposed to protect yourself when you arrive at the clinic so that you don’t get infected by the disease from here at the clinic

RES: Yes you would stand 1 meter spaced with the person who would have asked if he/she if the last on the que, so you would stand 1 meter whilst you are wearing your mask, when entering you sanitize your hands inside

ZM: Alright looking at the time when we were in lockdown or this time we are struggling with corona virus pandemic can you say the number of people are being helped at the clinic has changed than what used to happen back then when people were not yet in corona virus pandemic

RES: On people being helped

ZM: On number the number of people that goes to get treatment at the clinic can you say it has changed because of corona

RES: Yes I has changed because here it used to be full with people who wanted to get treatment but now you would think that maybe it’s not working today but it will be working

ZM: Okay looking at the issue of corona we hear that it brought many problems at home because the fathers were spending days at home people were allowed to travel they were told to stay in their homes, you looking at your home did you have any challenges because of the issue that you were in lockdown seated at home and your husband would be there

RES: Hmm I think there was a time were left the 2 of us me and my husband the children were taken when it started

ZM: Who had taken the children?

RES: They were taken with my sister’s child she went with then, she stayed with them and we were left just 2 of us but at home it will be difficult

ZM: What was difficult?

RES: The husband will be……like our husband who didn’t have jobs to go to you will be seated expecting that maybe he will do this for him to go out and look for something there was nothing, he would be afraid that if I go out I don’t have the letter for him to go and repair cars it was not allowed

ZM: Hmm

RES: Some were not even wanted to be seen he spent a long time not going

ZM: What about on the issue of collecting medication, taking medication and giving the baby medication did you ever had any challenge because you were all spending the whole day at home

RES: Ahh I didn’t

ZM: Does your husband already knows your status?

RES: Yes

ZM: Does he know that you take medication?

RES: Yes

ZM: What about looking at the issue of money at home did you have any challenges?

RES: Ah we had to the extent that t home you will end up arguing thinking that my partner is doing it purpose not to (laughing)

ZM: That he is doing what explain your story

RES: You be saying he is doing it purposely maybe he doesn’t what to look for money, but he will be seeing that on the side that I go it’s not working, and sometimes he would go out and stand outside then he says for me to go it can’t then you would say he’s doing it on purpose how can he say he can’t go out how are others walking

ZM: Alright, looking at the issue that women encounter many challenges because they are women being oppressed and what, can you say that the issue of child care has affected mothers during the time of corona virus that was no school that they were going all the children were at home looking at the mothers, do you think that it affected many women for them to be able to get PMTCT services can you say there are other mothers who failed to get these services because there were children at home who were not going to school

RES: Ah on that there is no one that I saw

ZM: Hmm

RES: On that there is no one

ZM: What bout looking at the issue of that at home the fathers are the ones who make decisions or they are the ones who choses what to eat that day or to do what do you think it was affected because of corona that issue that everyone is at home you are just looking at each other at home

RES: Ahh

ZM: Is there anything that affected issue because maybe the father is now there maybe if it’s in the afternoon you are the one who makes decision that we are supposed to eat this then do this or I want to buy this or I want to do what, but now because the father is now there maybe e the who is supposed to decide

RES: Yes he can make you eat what you don’t want then he says that’s what is there let’s do that

ZM: Hmm

RES: If its sadza and okra they will say let’s eat that and maybe you don’t want that, you are used to eating delicious so you will be arguing then he says if you don’t want that’s it that’s what I have managed to get today let’s eat that

ZM: Alright what about looking at do you ever feel scared for your child that because of corona I don’t know what will happen to his/her health in future

RES: Yes sometimes I feel scared because the children like now they are seeing like there is no COVID-19-19 you would hear that I saw your child at the party they you would say isn’t this child not hearing what is always being said he is going to get infected whilst he/she is wherever he/she is

ZM: Hmm

RES: So he will not be scared but you are the one who will be scared for him/her

ZM: What about the youngest the infant is there anything that you are scared of on his/her health because of the issue that he/she was born during the time of corona virus

RES: Yes am fearing for him/her on that they should continue giving these pills cotri for him/her so that she keeps on taking because now if they say we no longer have maybe Will not have the money to buy for me to buy for him/her then he/she might be affected

ZM: Hmm

RES: He/she can no longer gets his/her medication

ZM: Alright what about you as the mother is there anything that you are fearing on your health because you are living in the era of corona virus (just join them together that the time they were pregnant and the time they were……) you said you as a mother is there anything that you fearing for yourself because of the issue that you gave birth during the time of corona and you are living in the era of corona as a mother is there anything you are scared of on your health?

RES: Yes I will be afraid that our pills the day I will go there they won’t say today we don’t have or will be given the wrong ones that’s what am afraid of ,if they could continue doing that for us like these days we are just coming to take there no money that is being paid

ZM: Alright

RES: So I can say that they should continue like that so that our health will be okay

ZM: Alright the government of Zimbabwe has implemented different measures in trying to reduce the spread of the virus which includes that if a person is suspecting that you have met with someone who has the disease you must go in what Is called self-isolation, if you are sick go into what is called quarantine that you will be staying alone or don’t travel a lot or don’t gather or crowd. Looking at these things that were implemented by the government do you think it works in the community that you stay, let’s say looking in your house do see something like self-isolation or quarantine can be done let’s say your husband has been infected is he able to do 14 days in isolation staying alone not mixing with others doing his own thing, are you able to because you have met someone who has it, to isolate yourself do you see it happening looking at the way you live or how other people in your community live

RES: Haa it’s not happening to then because there were someone we had that this was infected he/she is walking people are running away from him if he arrive where they will be, he was told to stay indoors but he is not indoors but he is walking

ZM: What makes people around after they have been told to self-isolate?

RES: They are people who take things lightly like it’s a lie that this disease is not there, they will be seeing like it’s not there but it’s there

ZM: What about the houses that people stay do they allow them to do self-isolation and self-quarantine looking at the community that you came from?

RES: Uh our houses cannot do that

ZM: If you are saying our houses cannot what do you mean?

RES: Because they are packed together so they can’t

ZM: Alright what about looking at the issue of roadblocks that a person was being asked a letter when you want to travel or what do you think it affected you as a person who is living with HIV

RES: Ah as for me I have never travelled that I will boarder where there was need for a letter I don’t know to others who were travelling how it affected them or

ZM: Alright, Looking at child care in your community can you say there something that changed looking at that we are living in the era of corona virus, looking at how children were being taken care of in your community is there anything that changed in the community pertaining the way you are living with your neighbors or on the back lines looking at child care

RES: Yes there is something that changed

ZM: What Changed?

RES: Many children are no longer moving around like what they used to do back then and you will see even a child without a facemask you see him going back to take a facemask and wear, they were no longer moving around like they used to do

ZM: Alright looking at corona virus disease and the community that you come from do you think people are they still panicking or they no longer care about it

RES: Because panicked a lot at first now you can see that ah there’s nothing

ZM: Why are they saying there is nothing?

RES: They are walking freely you will meet some without even a facemask or what they don’t wear some they can be holding it in their hands walking and going

ZM: Alright but what was happening at first you said people panicked at first?

RES: At first people were wearing when they heard about facemask they were wearing even if when they were told to stay indoors people were staying indoors

ZM: What can be done in trying to reduce the negative impacts that were brought by corona virus in people’s lives in your community, what can be done by the government or with other Organizations in trying to reduce what have been affected because of corona virus in your area?

RES: Many people are not going to work they are seated at home

ZM: So what can be done?

RES: If they can open the companies that are closed so that some can be able to go to work, thieves has increased that what they are that this seeing can do they are stealing from people

ZM: Alright thank you mother those are all the questions that I had I don’t know if you have anything you want to ask

RES: No I don’t have
