# Supplementary material for: “Getting pregnant during COVID-19 was a big risk because getting help from the clinic was not easy”: COVID-19 experiences of women and healthcare providers in Harare, Zimbabwe
Source: PLOS Glob Public Health. 2024 Jan 8;4(1):e0002317. doi: 10.1371/journal.pgph.0002317 (PMC10773929; doi:10.1371/journal.pgph.0002317)
Supplement: S1 Data — (ZIP) [file pgph.0002317.s003.zip › Data/Mothers/Participant 14.docx]

**Interviewee’s Gender: Female**

**Interviewee’s Age: Around 34 years**

**Interviewee’s Initials: Mother _ _ _ _**

**Length of Interview: 29:16**

CM: Now we can start can you please kindly speak louder since it’s raining so that we will, be able to hear your voice, right?

RES: All right

CM: Can you tell me about yourself, where do you stay and how old are you, who do you stay with, and do you work or are you married?

RES: I stay in XXX on number XXX, I don’t work, and I have 4 children twins one who is 9 and the other 2

CM: Hmm

RES: Plus, the pregnant that I am now the pills were not compatible with me, so I have a husband, but he hasn’t paid lobola he just paid ‘Tsvagirai kuno’ (Did not pay bride price)

CM: Okay Can you tell me what you understand about coronavirus/COVID-19

RES: I can say that it’s a disease that came without anyone expecting it will happen in the country.

CM: What do you understand about COVID-19?

RES: I understand that you must listen to the rules and regulations of COVID-19, you must sanitize and do short distances that’s what I understand.

CM: Sorry I did not understand that.

RES: Short what is it called?

CM: How is the short distance done?

RES: Spacing and not sitting close to one another

CM: Okay social distancing?

RES: Hmm

CM: Okay how do you feel about COVID-19 personally, how do you feel?

RES: I have accepted it; we have accepted that it’s a disease that just come

CM: What else do you feel about COVID-19?

RES: Do you mean on being sick or?

CM: How do you see it?

RES: It’s there and we do not deny that it's among us to stay

CM: What are doing to protect yourself from COVID-19?

RES: To protect?

CM: Personally, what are you doing to protect yourself?

RES: If I feel that I have a flu I drink lemons that’s what I can use

CM: Hmm

RES: Some say zumbani (African Herb) can prevent

CM: Is there anything else that you are able to do

RES: They say steaming

CM: What about your family what are doing?

RES: That’s what I am doing

CM: How do nurse community health workers perceive the COVID-19 situation and how are they taking the situation?

RES: They encourage those who are sick they must go to the clinics or to call if they have any symptoms. There are phone numbers of people who are dealing with COVID-19, and they can come and you get help.

CM: Okay what else do they encourage?

RES: Hmm on that I don’t know

CM: Okay now we want to talk about different times, 3 different timelines in your life I am going to talk about these 3 times that I have written on this paper, this one stands for the time when you were pregnant right?

RES: I am pregnant right now

CM: When you are pregnant right now, this one stands for the time when you were tested, this one stands for the time when you’re going to give birth, but for you, I’m going to talk to you about your time of getting pregnant and time of being tested right

RES: Hmm

CM: I would want you to explain to me what happened or what happened to you from the day you knew that you were pregnant up to today.

RES: I can say that I was using family planning. I was on Jadelle I took time because I had twins.

CM: Hmm

RES: So, after the twins that’s when I got pregnant whilst on that Jadelle so it worried me that I got pregnant whilst I had a Jadelle how did that go?

CM: Hmm

RES: Then I was counseled, by others that it happens then I said maybe I am no longer repeating again, then I said Jadelle has failed to work on me so let me do family planning pills on this pregnant

CM: Which family planning?

RES: Pills

CM: Hmm

RES: Then started taking family planning I saw that it has failed again so I’m seeing…when I started, I said maybe I wasn’t seeing well when I did not see my menses

CM: Hmm

RES: Then I had 2 months that’s when I saw that my stomach was still growing big so I just accepted it because there was nothing I could do, that’s what was there then I told my husband that that’s what is there

CM: Hmm

RES: So that time he wasn’t….even now he is not going to work he is seated, so these are things that are troubling me that how am I going to do it up to now

CM: Okay on the issue of your daily living, how is your life different from when there was no covid and now there is covid, what difference is there on your daily doings, on what you are supposed to do

RES: It’s the same because sometimes you can’t find anything to eat, so life will be hard for me that what am I going to give the children, like now the twins and the one who’s 9 years they are supposed to go to school their father is no longer going to work

CM: Hmmm

RES: So I’m seeing that things are now worse, how am I going to do it so that the children can go to school

CM: Before covid came where these children going to school

RES: They did not have……..One of them was going

CM: He/she was going where?

RES: He/she was going to what is called Destiny so at Destiny they are no longer going because I no longer have the money

CM: So he/she stopped when at Destiny?

RES: When others started he/she is not going

CM: Okay, what about in your family you have talked about the issue of hunger and your living is there anything that has changed, when you talked about hunger during the time of covid before covid came have you ever faced hunger

RES: Haa I was hustling joining things

CM: Hmm

RES: Wasn’t lacking something to eat but now it’s hard for me

CM: What about when you were not yet pregnant and now that you are pregnant is there anything that has changed in your life during this time of corona?

RES: Nothing changed

CM: Nothing changed?

RES: Hmm

CM: On the issue of accessing treatment services what were you encountering that has changed from before coronaviruswas there and now, when you come to the clinic to seek treatment services, is there anything that has changed at the clinic before coronaviruswas there and now there’s is coronavirus

RES: I can say hmm I don’t want to lie if I go to the clinic wanting to when my date of medication is due I will be served well then I will be given my medication then I come back home

CM: Okay there’s nothing that has changed during the time of covid from last year?

RES: Haa nothing

CM: How has covid affected your mental health or your way of living, mental health the issue of thinking that you will reach to stress?

RES: Yes we reached a point of thinking deep that God where are we going reach whilst we are in this situation

CM: You think deep on what issues, what can you say coronavirus had affected this when you think deep?

RES: Maybe I could have been in a better position, maybe my husband would have been working

CM: Is there anything else?

RES: No there’s nothing

CM: Okay can you explain to me about services of preventing the mother from getting infected with the virus or not to transmit the virus to the baby in the stomach

RES: You prevent if you are pregnant like this and you are on medication you take your medication on time

CM: Okay what else are doing to prevent or the services that you are getting from the clinic to prevent, what you are being helped with by the clinic?

RES: I can say the medication that’s what I am being helped with, to prevent because I have not yet registered I don’t want to lie I want to say the truth

CM: Okay you have not yet registered now

RES: Can’t find money for me to go and register

CM: To register?

RES: Hmm

CM: So now what are doing when you come to the clinic let’s say they had seen that you are pregnant, how you are doing it when you have come to collect your medication?

RES: I walk on foot coming here

CM: No when you have arrived here what else are they discussing with you?

RES: Nothing I last collected in…

CM: Did you tell them that you are pregnant?

RES: Because I collect from it was closed then I went to Red bull so at Red bull we were just victors we were just served with our medication

CM: Where were you collecting from?

RES: I collect from Old Tafara that’s where I collect from so I haven’t go back to review, I go back to review in April

CM: You’re going back to review in April

RES: Hmm

CM: What about here?

RES: I last collect from here long back

CM: Why?

RES: Was transferred my baby that was talking about was still an infant then they said correct from your nearest clinic don’t walk with the baby

CM: So now with this pregnant have you come back to use this clinic or?

RES: I will be given permission there so that I can come back here if they give transfer they will change us

CM: Can you tell me when have you started taking your medication or the program of preventing the baby from getting infected with the virus?

RES: I started ehh my first born when I was pregnant with my first born long back in May 2012 that’s when I started up to now

CM: Okay can you tell me about the services of preventing the baby from getting infected with HIV virus that you are getting, have you been getting them from here from the onset of covid?

RES: Hmm you mean…

CM: You are not getting treatment programs right?

RES: Hmm

CM: Okay had forgotten sorry on that, eh what do you thing are the things that you are encountering in your community how are people being affected in seeking treatment services

RES: In the community that I live in?

CM: Yes because of COVID-19 how people were affected or how were they disturbed a person is sick be it headache or what that he/she is sick of at home, what it’s the problem in accessing services or treatment services?

RES: Hmm I don’t want to lie those who go they will comeback saying we were served well I don’t want to lie

CM: They go and say we have been served well?

RES: Uhm

CM: Okay is there anything that it’s affecting

RES: No

CM: People during the time of covid what where they doing if they have gone to the clinic what was happening?

RES: Uhm I don’t want to lie when covid started at the clinic I was walking that mush so there is nothing I was seeing

CM: Okay

RES: But I know that people were stopped outside the gate being served and taken temperatures that’s all

CM: What about you when you last collected your medication what happened?

RES: We were seated outside the gate and we were served outside the gate

CM: Okay what about other how is it affected then, who would want to get treatment for other disease, a person has a child who is sick the child has earache or headache what is it affecting?

RES: Hmm I would lie on that

CM: Okay do you think you have all the information that is needed during the time of COVID-19 in this time of COVID-19 do you have all the information that is needed

RES: Hmm

CM: Do you know where to go when you are pregnant or for you to be checked or where to do delivery or to give birth?

RES: You come here at the clinic

CM: Do you know how you can travel including mode of transport and what is needed when travelling?

RES: You look for transport but if you don’t have transport you can walk slowly until you reach where you are going

CM: Do you know what to do when you have arrived at the clinic?

RES: They will say a person must take her bags if you have been accompanied a person must take her bags then walk carrying your bags alone then you get in and you will be checked

CM: Alright these days of COVID-19 do you know what you are supposed to do when you arrive at the clinic?

RES: What I know is you will arrive and taken outside the gate then you will be sanitized and temperature as well

CM: Okay did you see anything that has changed in the clinic from the onset of COVID-19 we are talking about the issue of waiting or the issue of quality of service is there anything that has changed on the quality of services?

RES: The clinic closed we don’t want to lie, they closed saying there are people who were infected with covid so the clinic did what they have closed

CM: What else happened?

RES: Hmmm

CM: Is there anything that happened?

RES: Ugh that’s what I know that the clinic closed

CM: What about on the issue of waiting when you arrive at the clinic the time you would wait and the quality of services how were they?

RES: You would take time if you have come in the morning you would leave at 2 or at 3 waiting

CM: Okay I want to talk about the way you live at home are there any challenges that you have encountered for you to be able to come to the clinic during the time of lockdown

RES: No

CM: There are no challenges that you have encountered?

RES: Hmm

CM: What about on the issue of status discloser be to the people that you stay with, the issue of money for transport is there any challenge that you have faced?

RES: Hmm you would borrow from a person 2 days later they will come back to you and there is nowhere you could have gotten it

CM: Are there any challenges that you have encountered on collecting your medication during the time of lockdown

RES: No

CM: There are no problems that you have encountered?

RES: Hmm

CM: In your view do you think the issue of how the women live sorry….on the issue of gender dynamics of the difference between women and men and things that affect women for them to be able seek the services of preventing children from getting infected with HIV in the stomach, how do you see it in your family and in your community the issue of differences of gender, the difference between men and women and also the issue that stops women from seeking treatment services for children, how do you see it in your family or in your community?

RES: A person is supposed to go if they had said that’s what’s on you, you must go and get checked

CM: What else?

RES: Hmm

CM: What are about the issue of women taking care of the children and protecting them did that affected them from being able to go and get treatment services and preventing HIV infection if they are pregnant

RES: Child care

CM: Hmm

RES: You will take care of them well, preventing you come to the clinic they you prevent or if he/she is taking medication you come with him/her if his/her time is due you come with him/her then prevent and given his/her medication then go back home

CM: Okay you know that when we are at home women has the burden or the work or taking care of the children at home

RES: That’s true

CM: That job of taking care of them that the women do during the time of lockdown taking care of children and protecting them, did it affected women so that they fail to get services so that they fail to get services to prevent the baby from getting infected with the virus in the stomach

RES: No it didn’t affect

CM: Okay what about women not having money or to have other things they have on the issue of wealth, did it affect them on being able to seek the services of preventing the baby from getting infected with virus whist they are in the stomach

RES: Because have been selling them the police came then they take the things they go with them you will be confused that how am I going to take care of the children

CM: Okay what about on the issue of seeking treatment programs women sometimes might not have the money sometimes they don’t have anything did that affected then in being able to get what we call…for preventing the children from getting infected with the virus

RES: Some can do that just that you don’t ask some people, you don’t ask that how is it going

CM: Okay women we know that they don’t have power to give sometimes they don’t have power to give decisions in their homes and in the community did that affected women so that they can be able to prevent the baby from getting infected with the virus whilst they are still in the stomach

RES: No it didn’t

CM: Okay are there things pertaining the way you live that affected your health that has been brought by coronavirus?

RES: Haa there’s nothing

CM: Okay the government of Zimbabwe has changed things when COVID-19 came they said that we making people stay home like if you meet with someone who has covid you stay at home for day for 2 weeks, they stopped people from travelling from one city to another or another country ,they closed schools ,they closed borders. How do you think this affected you or other women that are in your area?

RES: It affected a lot it affected many things

CM: What has it affected?

RES: Because people were used to that when you thought of travelling you can go or if you have been called by your relative if it’s far people can go so it affected a lot of things we don’t want to lie it affected many things schools children will no longer know where to start

CM: Is there anything else that affected

RES: Ahh there is nothing else

CM: What about the issue of roadblock if a person is supposed to go to the clinic we know at the roadblock they were asking where you were going, what do you want…..(how many did you do, ask her)

RES: There 1 one left there

CM: Are there others who had gone

RES: There’s someone who’s there I don’t know

CM: Like you are at the roadblock you are being asked give us the card, where are you going to collector pills give us the card, how did it affect the roadblocks what did it affect to people who wanted to travel to go and collect their medication

RES: Hmm I didn’t hear about that I don’t want to lie I am supposed to tell the truth

CM: Okay on the issue of living together in the community and child care in the community where you stay, is there anything that has changed pertaining child care before COVID-19 came and how people were staying in the community and after COVID-19.Is there anything that has changed I your community?

RES: The way we were living is still the same

CM: Hmm

RES: Plus when covid came each person must protect his/her children that you must play here because there is a disease called covid

CM: Okay what about COVID-19…..sorry it’s okay …COVID-19 is a thing that I can’t say affects right, how can we say it is in this community, What awareness I can’t say awareness, can we say concern of COVID-19 in your community, how much of concern can we say it has…. (what can we say concern Harty- I always say it like that …people are still being concerned to what extent) they are concerned to what extent or they are still looking at COVID-19 things to what extent ( Harty – are they still taking it serious ) are they still taking it serious or they are taking it easy

RES: Ah we take it serious because we know that the disease is there we know that it’s there

CM: What about others in your community how are they seeing it?

RES: Haa that’s it

CM: How do people in your community feel about seeking treatment services from the hospital or clinics?

RES: On that I don’t know

CM: Is there anything that can be done to help us to reduce negative impacts that were brought by COVID-19 in your community. Is there anything that can helped that can be done by the government or your community that can help (there are 2 who signed) is there anything that can be done

RES: Uh Uh

CM: There’s nothing that can be done

RES: Hmm

RES: Thank you for the nice discussion, we have finished our interview, thank you for coming

RES: Thank you too
