# Supplementary material for: “Getting pregnant during COVID-19 was a big risk because getting help from the clinic was not easy”: COVID-19 experiences of women and healthcare providers in Harare, Zimbabwe
Source: PLOS Glob Public Health. 2024 Jan 8;4(1):e0002317. doi: 10.1371/journal.pgph.0002317 (PMC10773929; doi:10.1371/journal.pgph.0002317)
Supplement: S1 Data — (ZIP) [file pgph.0002317.s003.zip › Data/Mothers/Participant 2.docx]

**Interviewee’s Gender: Female**

**Interviewee’s Age: Around 24 years**

**Interviewee’s Initials: Mother**

**Length of Interview: 53:29**

HM: The first thing that I am going to do is to ask where you stay who you stay with, your age, do you go to work, how you live at home, are married, so let’s start by talking about your age how old are you, where do you stay?

RES: Alright I’m 24 years

HM: Hmm

RES: I stay in XXX in the new stands that are….

HM: I want you to speak up because the voice doesn’t come out in full because of the masks that we are wearing.

RES: Alright I’m 24 years old.

HM: Yes

RES: I am staying in new XXX ward 19.

HM: Okay are you married?

RES: Yes, I am married.

HM: How many children do you have?

RES: This is my first child (Pointing at her child)

HM: At home how do you live, who do stay with?

RES: I stay with my husband

HM: Who else?

RES: I stay with my husband and child only

HM: You’re just 3?

RES: Hmm

HM: What about work, do you work? were you working before COVID-19?

RES: Before COVID-19 I was going to work when COVID-19 came that’s when we were stopped at work up until now.

HM: Where were you working before COVID-19?

RES: I was working in XXX shopping center, at the butcher that is opposite the OK shop.

HM: Okay you were working in a butchery?

RES: Yes in a butchery

HM: So right now there is no work you were stopped?

RES: Hmm

HM: Are people not buying meat and eating it?

RES: The person who I was working for is the one who closed his butchery.

HM: Oh all right let’s move on knowledge and your views about COVID-19. Can you tell me what you have heard or what you know about COVID-19?

RES: What I heard is that COVID-19 is there in Zimbabwe

HM: Hmm

RES:It was killing people in Zimbabwe and all over

HM: Hmm is there anything else that you have heard about COVID-19, there are so many things some were broadcast on radios, and TVs, here at the clinic they put on fliers and posters what have you heard?

RES: I heard that when you are traveling if you arrive at a place like a clinic you first get tested for temperatures, and sanitize hands, we must always sanitize our hands and always wear masks.

HM: Alright what do you understand about the COVID-19 pandemic?

RES: Uh

HM: Your views on the COVID-19 disease.

RES: COVID-19 disease

HM: Hmm your view what kind of a disease is COVID-19?

RES: Ahh I don’t understand it

HM: Your own understanding that this disease does kill, it has destroyed many countries the way you understand.

RES: COVID-19 is killing many countries and destroying people are dying for sure with this COVID-19 disease.

HM: All right, how do you feel personally about COVID-19?

RES: I was afraid of COVID-19 because I heard that it was killing people with underlying health conditions like HIV. People were showing us videos of how people were getting infected in hospitals, and I imagined being in the same waiting area with someone with COVID-19 or being attended to by a nurse with COVID-19 and I was so scared, and I told myself I will only go if there is a problem.

HM: All right now are you still afraid we are seeing that people are just walking without masks or what is happening?

RES: I’m still afraid because I’m scared of dying

HM: You’re scared of dying?

RES: I’m afraid of getting infected by COVID-19

HM: Where you stay were there people affected in your area?

RES: Haa I never heard of anyone I would lie

HM: You’ve never heard of anyone?

RES: Yeah

HM: Alright what do you think can be done, or what changes or what measures can be done in response to COVID-19, a person on his or on her own can he/she do, what measures or what changes that can be done by a person on the level that me personally I think that for me to respond to COVID-19 am supposed to do this or do that, what measures can be done by a person on his/her own or what changes can be done by a person?

RES: Uhh

HM: A person can do….you personally what can you do what measures can you implement or what changes can you implement so that you can respond to COVID-19?

RES: Ahh so far I still want to stay at home me personally I don’t want to move around yet even to look for a job haa I’m still scared

HM: So you personally you still want to stay at home?

RES: Me personally

HM: Is there anything that you can do or that can be done by other people looking at COVID-19 disease what changes can they make, is there anything that they can do?

RES: Uhh I don’t know about others

HM: No you will be thinking that I think that if people do this and that or if a person can do this or that it might help to reduce the spread of COVID-19 or looking at the problem of COVID-19 if a person does this and that it might be helpful

RES: If people could be able to follow what is being said about protection wearing masks if you are able to get tested for COVID-19 then tested so that it doesn’t spread to other people

HM: Is it, what about on families what measures can be done by families looking at the issue of COVID-19 or corona virus

RES: On families

HM: Yes family what can they do what measures or changes are you making or a family can make in response to COVID-19, what can they do what can a family do in response to COVID-19, what measures can they implement what changes can be implemented by families, what do you think a family can do?

RES: Family

HM: Yes family what can they do, there is nothing they can do, they must leave it like that

RES: No

HM: So what can they do?

RES: They are supposed protect

HM: How do they protect the measures we say if we put this and that here it can help us to prevent or if we were doing this we are not supposed to do that it will put us on risk of getting infected with COVID-19 virus.so what measures can be done or what changes can they do that what we are asking, that what can a family do, what can they do, what measures can they do if they have anything what changes can they make …(heyyy) there’s nothing they can do, you don’t know they can do?

RES: I don’t know I also want to be told

HM: What do they do?

RES: I don’t know I also want to be told

HM: No we have come to hear from you you’re the ones who stays here in XXX and Tafara that what was happening during COVID-19 we were not here we want to learn so that we know what was taking place right

RES: Uhm

HM: Alight, in your view health workers or those who work with people or clinic workers how are they perceiving the situation that is there of COVID-19 how were they seeing the situation that was there of COVID-19, the nurses those who does counselling, those who give medication how were they seeing it the situation that we are in of COVID-19?

RES: The situation that we are in

HM: Speak out loud

RES: They were taking it serious, they take it serious that issue of COVID-19 sometimes you would come here like the days were coming to scale you would arrive and returned at the gate saying that they are not scaling people because of the issue that there is COVID-19, we will call you when we are free, after getting protectives, we don’t want people to crowd

HM: Hmmm

RES: They were teaching us many things

HM: What were they teaching those many things are the ones that we want to hear so that we can learn that the nurses and government workers and health workers how were they seeing it, the situation that we were in during COVID-19 during COVID-19, how were they seeing the situation?

RES: The time of lockdown it was a time that was hard for everyone it was hard

HM: Even for the health workers why are you saying so?

RES: ---------

HM: Alright when did you born this child?

RES: On the 25^th^ of October

HM: Last year?

RES: Yeah

HM: So when did you fall pregnant?

RES: In February

HM: February 2018 2019

RES: 2019

HM: 2019?

RES: Hmm

HM: Alright and was born before COVID-19 started?

RES: No 2020 February 2020

HM: That when you…?

RES: That’s when I got pregnant and I gave birth in October

HM: So you got pregnant when COVID-19 was about to start

RES: When it was about to start

HM: So I want you to take me through that process that when you got pregnant what was happening what did you do, the whole process that you did from when you started to know that you were pregnant looking at the issue of prevention of mother to child transmission that of PMTCT, then when you get tested I want to know the process that you came here and you did this and that they you go here on delivering and lactation, delivering giving birth to the baby and breastfeeding the baby that what happened, so can you tell me how you walked from when you stated to know that you are pregnant February 2020 that what happened up to when you gave birth to your baby and breastfeeding your baby all the process that you did her at the clinic ,at home how was it going I want to understand

RES: When I got pregnant in February I was working here in XXX I was staying at home with parents my mother that’s who I was staying with that when I…I didn’t manage to tell them that I was pregnant that time I stayed for long keeping it as a secret

HM: Hmmm

RES: But I was born HIV positive

HM: Alright

RES: So I was already on ART that’s when I come to register here, when I came to register then I registered

HM: Hmm

RES: Then I went to my husband’s house I went when my pregnancy was at 6 months that’s when I went, so I was coming to work

HM: Soo you were not tested or what?

RES: No I was already knowing

HM: Your status

RES: Yes when I came that’s when I came with my husband, my husband is negative

HM: Your husband is negative

RES: Yes

HM: How did he take it when you were dating did you tell him your status did you disclose your status?

RES: When I loved him I didn’t manage to disclose so we just went for tests I was always saying lets go and get tested when we were still dating that’s when he started to know, I was already knowing but I was scared of disclosing

HM: To tell him?

RES: Yes

HM: So went together and tested and he came out like that?

RES: Yes but the parents rejected me saying that ahh is she is positive leave her then he said what about my child this is my pregnant that’s when he refused, that’s when he ran away from his parents and we started staying on our own

HM: Alright

RES: The parents didn’t want the positive but the owner wanted

HM: Yes his parents dint want?

RES: Yes

HM: They didn’t want but do they want their grandchild

RES: Yes they want their grandchild

HM: But they don’t like the mother

RES: Yes

HM: Alright let’s move on then what else did you do?

RES: That’s when we started staying alone when we started staying alone I wasn’t working I had just left the job

HM: How was life that time we were in already COVID-19 that time?

RES: Things were hard, things were hard because my husband doesn’t go to work and I was also not working so life became hard I don’t want to lie

HM: So he was already not working before COVID-19 your husband

RES: Haa he doesn’t work

HM: He has never gone to work?

RES: Hmm

HM: Alright, you are the one who was working

RES: Hmm

HM: Alright so what happened in life who was helping you when you were staying alone what was happening?

RES: When we were staying alone we were helped by my sister who I comes after she works she stays in Cape town she’s the one who helped me from rent and everything, all the money she’s the one who was helping me every month she was sending

HM: Money in COVID-19

RES: Food, money for rent because we were renting

HM: What about mum?

RES: Ahh mum it was here and there plus my real parents passed away so I was afraid so for me to keep on troubling them

HM: So you were staying with your aunties

RES: Aunties my parent passed when I was still young I don’t know them

HM: Alright then what did you do?

RES: That when we started staying helped by my sister helping us helping us until my pregnancy reached 9 months then we came here

HM: So preparation and what it’s your sister who helped you?

RES: Yes she’s the one who helped me

HM: What about husbands’ relatives?

RES: Husbands relative never loved me from the begining, from when they knew that I am positive they never liked me

HM: They never liked or to treat you well they look down upon you do they stigmatize you, do they talk whilst you are listening what do they do

RES: Yes like when I go there because I stayed at their house they would talk and you will be hearing it ,then came and chase the father and the mother both criticizing then I said it wasn’t my choice to be positive but I think they don’t understand that

HM: So then you gave birth to the baby what happened was the baby born negative or positive?

RES: I gave birth at Mbuya Nehanda

HM: Yes

RES: That’s where I was done Caesar, I born my baby negative

HM: Alright what happening at Mbuya Nehanda they said how are you supposed to take care of the baby was he/she given the medication that he/she is supposed to be given?

RES: Yes when I gave birth at Mbuya Nehanda that’s when I was given Nevirapin that I was supposed to give him/her

HM: Hmm

RES: I would give him/her every evening when we are about to sleep

HM: Is the baby still taking the medication?

RES: Yeah they changed for him/her, she/he stopped nevirapin when she reached 6 weeks

HM: Alright at six weeks that when they change the medication?

RES: Yes

HM: Hoo what will she/he be given?

RES: Cotrimoxazole

HM: She will taking cotri only and mother’s breastmilk?

RES: Yes

HM: You talked about that life is hard the family wasn’t supporting you, there was no money what about now when you were in the second lockdown what was happening?

RES: Ah to me nothing has changed yet

HM: You can’t find job, the husband is not working?

RES: He is just hustling but not real job they are just pastimes but the things have not yet get back together

HM: They are not yet better?

RES: Hmm

HM: It’s still hard?

RES: Yeah it’s still a bit hard

HM: What about on the issue of accessing treatment services is there anything that has changed, have you ben always getting treatment here?

RES: Yes that’s where we always get treatment from

HM: Those programs of getting services of service delivery ah is there anything that has changed on how you were being helped when you come to treatment programs here at the clinic before and during COVID-19 that we are in can you see that there’s a different or not

RES: Hmm now things has changed people are getting treatment some are being helped well but during the days of COVID-19 real COVID-19 they would just count people that today we are treating this number we don’t want people to crowd the rest go back home, sometimes you could have bordered kombis to come then you will be told to come tomorrow then tomorrow if you come late …ahhh..Now it has changed people are…

HM: So your experience on the access of those treatment programs what do you say about it, what you have encountered during COVID-19 that you say in COVID-19 my experiences haa it was like this and that, did your experiences changed from before, what was happening before the coming of COVID-19 and now we are now were are in COVID-19

RES: Ahh things did change during COVID-19 but now it’s getting better

HM: Before COVID-19 what was happening?

RES: Before COVID-19 everything was normal

HM: What about during COVID-19 what has changed in accessing treatment services?

RES: Ah during COVID-19 it was difficult

HM: Were you coming to the clinic and fail to get treatment or failing to get services that you have been getting before COVID-19

RES: Haa there is no big difference but at the clinic you would come but what I told you that they would count the number of people

HM: Where they giving the reason that today why were they serving 10 only

RES: The issue of crowding they were saying they don’t want us to crowd

HM: Hmm

RES: Let’s say way back, back then before COVID-19 we would just come to the clinic whether you have come in the morning anytime you come you were being served then you go, but when there was COVID-19 they were saying if they take a number of people who had come in the morning the rest who are going to come you will come the following day then you do back home

HM: Alright

RES: But now it’s different so far it’s going back to normal its normal I saw it today I started coming today

HM: Can you say that COVID-19 affected your mental health, the way you think you brains and your health generally

RES: On health that when I….

HM: Did you get sick during the time of COVID-19?

RES: Yes I got sick, I got sick maybe some it was mixed with stress of what had happened, being chased and what

HM: You had stress and depression?

RES: Plus the fact that the husband is not working

HM: The brains were no longer stable?

RES: Yeah the brains where no longer in place

HM: When you got sick did you get treatment did you get treatment services?

RES: Yes I got

HM: Did you come and get help when you got sick?

RES: Yes

HM: What about emotional distress did you talk to the nurse or counsellors here at the clinic that that’s what I’m facing?

RES: I didn’t manage to talk to them

HM: You didn’t manage to talk to them?

RES: Yes

HM: You were hurting without anyone you could give your thoughts or to discuss with that things are hard for me I’m facing this and that?

RES: The person I see that is free is my sister I would discuss with my sister, she’s the one I would tell what was happening that time

HM: Alright your sister?

RES: Yes

HM: Is it still painful up to now?

RES: Yeah some other things….

HM: What were people doing to you that’s what I want to understand, the passion that you have must come out that’s how you will be able to heal

RES: I was hurt the most with the way they treated me when I went to my mother-in-law’s house

HM: What were they doing, what did they do to you, where they beating you, where they abusing you, where not giving you food?

RES: They didn’t beat me but their words (crying)

HM: They were saying hurtful words

RES: They were saying touching words mother

HM: Alright, they couldn’t accept your status that that’s who you are?

RES: Hmm

HM: Even up to now are they still doing that are you still talking are you still I good books with these people or you are just staying alone you are doing your own life with your husband and you child

RES: When gave birth that when I went to show then the child but I went when she was 2 months that’s when I went to show them, I went to show then just because she’s the grandmother she is the mother-in-law there’s is nothing I could do but ahh

HM: Didn’t she like the baby?

RES: They like the baby but even if they like my child and they don’t like me it doesn’t work for me

HM: Yeah it doesn’t makes you happy

RES: Hmm

HM: But at your house do they come?

RES: They have never came

HM: They have never came?

RES: Yeah

HM: Alright so you brains had a lot of stress and depression because of the way you were being treated plus getting sick

RES: Yes plus the fact that the husband is not working and I am pregnant I don’t even have anything

HM: Hmm alright it will be fixed with time

RES: Hmm

HM: Can you tell me about PMTCT the programs of getting treatment those ones that you get you and the child the time you delivered, when you delivered did you came with all the medication from Mbuya Nehanda or you were told to go back to your clinic where you were thats were you will be given the services of getting your medication and the baby

RES: At Mbuya Nehanda I can say that I was given for the baby, as a person who had been done OP I came with all the medication from there, the baby came with nevirapin from there

HM: Hmm

RES: Then they said that after six weeks you will be going to local clinic that where she will be tested again then tested and change her medication

HM: Hmm

RES: That’s where we returned for check up

HM: Hmm so the baby at 6 weeks was she tested

RES: Yes she was tested

HM: How were the results when they came our?

RES: They were negative

HM: They come out negative?

RES: Hmm

HM: So they changed her medication there

RES: At 6 weeks that’s when I stated giving her….that’s when we were given cotri

HM: Alright have you always been using this clinic for treatment?

RES: Yes

HM: Alright, can you tell me about the PMTCT services that get you and the baby from this clinic from the onset of COVID-19, from the onset of COVID-19 were you coming to collect the medication for the baby what you said cotri or it was said they don’t have what was happening

RES: Ahh I can say that from when I gave birth for baby we go to baby clinic not here

HM: Haa for the baby they don’t do it here?

RES: For baby No when I came I didn’t know they do it here so I started by going to Tafara

HM: Hoo

RES: So when I began to know I then came back

HM: So here are you still getting the child’s medication?

RES: Yes the medication you can get them

HM: When last did you collect baby’s medication here?

RES: 8 March

HM: You came and you get cotri, you got it?

RES: Yes

HM: What about you your mediation did you get there and find them so that you don’t transmit the virus to the baby

RES: Yes I got them

HM: Are you following what you are being told by the doctors or with the nurses so that you don’t infect the baby?

RES: Yes I am following

HM: What are doing are breastfeeding only without giving her anything?

RES: Yes its breastfeeding only there’s nothing that she eats I just give her with her cotri only

HM: Alright some will say at 6 months they start to stop breastfeeding you have decided to stop breastfeeding when she will be on what?

RES: Ahh when he/she is 1 year 6

HM: Alright when she is 1 year 6 months of breast-feeding

RES: Yes

HM: But will you giving her other things of it will be breastmilk only?

RES: When I delivered that were saying food some were saying food/porridge you will start giving at 6 months

HM: That’s when you will start?

RES: They said porridge you will start giving at 6 months

HM: Giving her both with her medication?

RES: His/her medication they said he/she will not stop until he/she has stopped breastfeeding and I have her tested again that were she will stop

HM: Alright can you tell me what you have encountered because of the problem of COVID-19 from its onset, your experiences that you encountered from the onset of COVID-19 everything that you encou

RES: From COVID-19

HM: Yes from the onset of COVID-19 be it at home be it treatment services for you and the baby, did COVID-19 made it hard for you what was happening?

RES: During the COVID-19 things were hard because of the issue of being stopped at work and that we were not allowed to move so there was nothing we were doing so we were staying at home

HM: What about treatment services?

RES: Treatment we were coming and get treated but that’s what I told you that if you come late…. Maybe let’s say we were coming late since we were used with that

HM: They are always open anytime

RES: But that time maybe you would arrive at 10 and you would be told people has entered already, you are coming back tomorrow, even if you come at 9 as long as people has entered. But we were being helped I don’t want to lie

HM: The clinic has never closed the clinic was there no a time that it closed, were people coming and get their services?

RES: There was a time when said there are people who were infected with corona that when it closed, I don’t know how many weeks, can I says they were weeks or days, I just heard on that I heard because those days I had come from my review all my things were sorted that’s when I heard that the clinic has closed

HM: But was is for a short period or?

RES: Yeah I was for a short period it didn’t take time closed

HM: Alright do you think you had all the information that you needed during COVID-19 that you can go to….. When you are pregnant you can be able to go for scale and being checked how things are standing, doing checking ups did you have that information?

RES: Can you please repeat I didn’t understand

HM: Do you think you had enough information during the time of COVID-19 we were in from last year up until now that when you were pregnant did you know that I can go to the clinic and get help ,scaled and done all the checkups that are done on pregnant women did you have that knowledge

RES: Uhh

HM: Did you know that even if we are in COVID-19 I can go to the clinic and get help ,get checked because I’m pregnant the whole process that is done to a person who is pregnant up until a person gave birth

RES: Yes we would come and get help

HM: You were being scaled?

RES: Yes

HM: They were scaling?

RES: Yes

HM: And do all the checkups?

RES: Hmm

HM: What about the services of PMTCT were they doing?

RES: Of PMTCT

HM: The one which is said to prevent the children from getting infected

RES: Ahh that I don’t know because like what I told you I started taking medication when I was still young so from when I get pregnant there is no anything that I was given I was told to take my medication

HM: Alright

RES: Until I delivered that’s the one I was taking

HM: What about travelling going to the clinic, the requirement of travelling how was it, have you been travelling was it easy

RES: We were travelling, you would tell them that I am going to the clinic if you have register you are given those books that what I was walking with, if you arrive at the police you show them your book then they will say go if you don’t have haa it wasn’t easy to travel but if you were going to the clinic you were not being stopped

HM: Alright were they not returning people home?

RES: Uh uh if you were going to clinic you were not being returned

HM: Alright so a person was encouraged to walk with their books that was their pass to go to the clinic at the roadblocks?

RES: For me that’s what I was using

HM: The police wasn’t troubling you?

RES: Yes

HM: What about knowing that if I arrive at the clinic what do I do, were people aware during the time of lockdown that right now there is a pandemic that is there so if I go to the clinic what am I supposed to do

RES: Yes people were aware

HM: Alright

RES: If you arrive at the clinic you start by waiting and get tested for temperature, taken temperature, at the gate there was water to wash your hands and sanitizers, they you start by sainting there then you enter inside, they you sit one meter apart

HM: Alright

RES: Plus those day they were not letting in all the people one time in the yard , they were taking few few saying you seat here you seat here if you’re served then others enters that’s what they were doing

HM: Alright, Is there anything that you have noticed or anything that you saw ,or the changes that you saw on how they were treating people at the clinic, that the stay they were staying how long was it, how was the quality of the services that they were given

RES: During people were not allowed to stay for long

HM: How many minutes were taken by people, let’s say you have come from home you were serviced after how long, how much time were you taking here at the clinic?

RES: Hmm I don’t know but it wasn’t taking time because they were saying they don’t want to keep people so the person who has arrived will be served and go haa it wasn’t taking time, it’s different from now on this COVID-19 ahh it seems like it now slow

HM: They are taking time to serve people during the first COVID-19?

RES: They didn’t want to keep many people

HM: What about quality of the service was it okay or it was just quick quick so that people can go out fast and go

RES: Ugh quality of the service

HM: Because maybe you would arrive maybe you would come then you discuss everything that you left home for, given all the time now there is COVID-19 you arrive then I stamp your card ,I give you your pill then go there is no chance that we are going to discuss or to say that I have this problem or counselling maybe it was no longer done because they were saying there is COVID-19 so that’s the quality of services did you see that there’s anything that has changed on quality of serviced that you have been getting and the one you were getting during COVID-19 are they the same

RES: Hmm it was different

HM: What was... that’s what am asking that what was happening?

RES: You were no longer given enough time to explain

HM: They were just doing quick things?

RES: Yeah

HM: Is that all that they were giving your little time

RES: Yes that’s all

HM: That’s all?

RES: Yeah

HM: Alright let’s talk about social issues at home, at home did you have challenges that you want to come to the clinic then you failed or you will be forbidden during the time you were in lockdown, did you encounter challenges that you want to come to the clinic but you fail

RES: Haa I have never

HM: You husband has never forbidden you from traveling that you are walking too much it is said that there is corona when coming to the clinic

RES: Haa when I was coming to the clinic he would understand that its clinic but travelling just travelling for a journey I have never travelled during COVID-19 he was refusing, but to come to clinic I was allowed I would come

HM: What about on disclosure have you faced challenges telling your partner your husband, in the family you talked about that you faced challenges that the your husband’s family failed to accept, what about your husband did you face any challenge or you didn’t he is a person who just accept from when you started when you were still dating he just accepted that I love my girlfriend like that

RES: On my husband I have never encountered any challenge I told him that I was born like that

HM: Alright what about financial situation at home how was it, you said it was hard you were no longer working

RES: It was hard that’s when we depending on those who work , that month if they send from rent and everything we were waiting to be given, me as a person who was pregnant with the lockdown there was nothing I was able to do

HM: What about challenges on taking medication did you encounter any you or child during the time of lockdown failing to take you medication correctly or failing to give the baby medication during the lockdown have you ever encountered that problem?

RES: Ahh I have never encountered that

HM: You were taking your medication correctly?

RES: Yes

HM: The baby you were giving her medication correctly?

RES: Yes the baby I was giving her medication correctly and I was taking my medication correctly

HM: You did not encounter any challenge?

RES: I have never encountered any challenge

HM: Alright what about on the issue of gender dynamics gender roles were women affected the most in accessing treatment programs accessing treatment especially pregnant women looking at the issue of preventing the babies from getting infected at your home or in your community how was it, looking at women

RES: Looking at women

HM: Hmm

RES: Hmm it was hard here there are days when they closed maternity that’s when people were giving birth in homes so we were afraid, a person would come to clinic and told that maternity is closed some were giving birth at the gate, like there was this other day when I came here ihhh I was afraid, I had come for scale

HM: Ehh

RES: Another women gave birth at the gate because maternity was closed

HM: They said maternity was closed what had happened?

RES: Haa I don’t know but those days they had closed they were not delivering here

HM: Alright so what were people doing all the women who were pregnant, where were they going?

RES: There were they were giving names were they were saying if you are due you can go to so and so you can go to Edith you can go to Budiriro and Chitungwiza

HM: Alright they were saying go to other places

RES: Yes that are working they were saying here we are closed

HM: Alright what about in the issue of childcare roles and duties, the childcare roles and responsibilities was it to heavy for the women or people were sharing with their husband’s, carrying together and helping each other

RES: Hmm it was the same

HM: It was the same?

RES: Hmm

HM: What was happening who was doing this job the most of taking care of the family, of talking care of the kids or other things that are needed yes child care roles and responsibilities

RES: Taking care of the children was the mother, she’s the one who was seeing about children, maybe the father had gone to look for part time since those had jobs that were not…that were not allowed to move around they were doing part time so that they can survive with family, the mother will be the one who will be at home and taking of the children who were not going to school

HM: What about on the issue of access to and control of resources how much were women affected, the resources that are found at home be it money, be it food, be it things that can be found in a family, how much were women affected in access to utilization PMTCT services in your family and in your community, looking at access and control of resources that if a person brings sugar they say it’s supposed to finish 2 months 2 kg or access and control of the things that they have found that they have

RES: Some of them it was for budget because they didn’t know that where is the COVID-19 taking us to, when are going to be allowed to start travelling so it was hard

HM: Was that hard too hard for the women or men?

RES: Yes because they were the ones who were always at home, they will be saying you are the one is at home plus they will be saying you are the one with the budget

HM: So it was heavy for them?

RES: Hmm

HM: Alright what about on decision making at home or in the community what was happening, how was it affecting women?

RES: Uhm

HM: On decision making how much were women being affected by that?

RES: Hmm I don’t know

HM: You don’t know what about ay your home who was making decisions, were you able to give your thoughts or to decide doing what you will be doing without being questioned by your husband that why have do done this or why has the happened

RES: We were sharing we would sit down and talk if he has accepted that’s it if he refuses then it has been rejected

HM: You husband would he accept what you have told him that haa baby dad I think that what you are talking about it can’t?

RES: My husband would refuse that

HM: So who had the powers on deciding?

RES: He was the one who was deciding haa he would refuse he wouldn’t accept it

HM: So the man had the power?

RES: Hmm

HM: The women did have the power to decide even in the community that you stay is that how it was

RES: Yes

HM: That men were ruling with an axe that what I have said is what has to be done even if to try to reason they will say I have said that this is what happening here

RES: Hmm

HM: Okay are there other social concerns that you might have you or the child on the issue of your health or child’s health

RES: Uh

HM: There is nothing?

RES: Yes

HM: Do you have the concerns that you might have regarding you or your child’s health as result of COVID-19, there is nothing?

RES: Hmm there is nothing

HM: Okay what about when you got sick didn’t you tell...? You only told your sister, who was looking at you, you talked about falling sick

RES: My husband even mom I told them

HM: Did they have care?

RES: These are the mothers who took care of me

HM: And you sister?

RES: Yes

HM: What about here at the clinic

RES: At the clinic was coming to get treatment until I healed

HM: So the time you got sick was the baby right she didn’t got sick?

RES: He/she has never been sick but I was afraid because when I got sick that when I was taken VL viral load

HM: Hmm

RES: Then it came the results were high so I was also stressed thinking about my child because they were saying you are now on risk that you baby can get infected ahh I had stress

HM: Alright you had a lot of stress?

RES: Yes

HM: Okay so did you get enough counselling that time?

RES: Yes I was counselled by the grandmothers so it ended

HM: Okay it is said that the government of Zimbabwe implemented laws that includes social isolation, not being allowed to meet at crowded places, it implemented travel restrictions people were not allowed to travel, it closed schools it closed borders, How much do you think it impacted or touched women in your community, the closure of schools, closure of borders , forbidden to travel, forbidden to do gatherings how did it impacted women or you personally how did it impacts you and how much or other women in your area how much did it impact them

RES: Hmm it was hard because some women they would be doing businesses that they will be buying goods in bulky and sell even in town vendor even at Mbare but some were not allowed because they were said they don’t have letters and your job doesn’t make you get a letter that I’m going to do vending

HM: Yes

RES: Haa it was hard and children that children you will be with them at home they want to eat haa the things were heavy

HM: It was hard for a woman

RES: Yeah

HM: Alright what about you have talked about people in your community what about you personally the closure of borders, failing to travel that you should not stay crowded how did it impacted you

RES: It impacted me on the issue of church they closed churches but if its working I was just saying my child was still young so it didn’t impacted me a lot

HM: Alright what about on the roadblocks if you want to go be it to town or coming here, from your home coming here were you passing through police

RES: No

HM: You were not meeting with the police?

RES: Hmm

HM: What was happening on roadblocks was people being asked about their status or what, what have you heard on people who were going who wanted to travel during the time of lockdown

RES: During lockdown if you wanted to travel that’s when you were asking for a letter then they will say where are you going then you tell them where you are going, do you have a letter if you don’t have they will say go back home people are not allowed to go and crowd where you are going

HM: In the buses were they entering saying to a person hold your letter or what

RES: The buses were bordered by people who have letters only, it was not bordered by a person who doesn’t have a letter, even if you border a private but when you meet with the police they would get you down and tell you to go back home if you don’t have a letter

HM: Alright relations with people in the community, community relations or childcare arrangements how was it going, the way people were talking did it change mother of so and so the next doors and neighbors in the community, did things change because of COVID-19

RES: It has changed a bit but not too much but people were no longer doing visits like they used to do that today you are going mother of so and so and spend the whole day there, it was just seeing each other 2 minutes

HM: What about taking care of children?

RES: Taking care of children ahhh that would want those with children, I don’t know, I personally do not walk too much

HM: Before COVID-19 came what were people doing in their area?

RES: Before COVID-19 came children were going to schools the mothers who work were going to work the fathers were going to work, the community wasn’t staying with people every time like what was happening during COVID-19, during COVID-19 haa everyone you would know that they would see the person there they didn’t go to work

HM: Alright are people still touched with COVID-19 in the area now or them no longer care about it?

RES: Hmm it’s now better since people were allowed to travel it’s not better so people are no longer touched that much but when people were not travelling it was touching people

HM: Why are you it was touching people?

RES: (Laughing) it’s because the way we leave grandmother the jobs that we do are different so people to be told that stay at home, social distance and what people were not used to that, people were used to go to their jobs as usual, you will be knowing that the children had gone to school, If you are going to set up your market at the road then you go, but on COVID-19 ahh

HM: There is nothing, but are people still concerned about COVID-19 in your community or they no longer care

RES: Haa it differs with the person but as for me personally I still afraid

HM: How you see it you would see that people are no longer care. People are doing this and that are they still concerned?

RES: Haa some are no longer because to follow the rules of preventing ahh the people, you would see some walking from here to that without even a mask, people are gathering but ahh

HM: They no longer care about it?

RES: Yes

HM: How do you think people in your area feel about accessing treatment services from the clinic or from poly clinic like here, people in your community how do they feel about the issue of getting treatment services from the clinic or from polyclinics do they like it, do they go, what do they do?

RES: Haa some people go to the clinic they like it

HM: What about other what do they do you said some go to the clinic what about others?

RES: Some are used that they stay at home

HM: At homes even if they got sick

RES: They will at their homes

HM: Alright, what do think are the measures or what programs can be done to reduce the problems that were caused by corona in your community , what can be done what programs can be done to mitigate the negative impact of corvid 19 or corona virus in your community

RES: In the community

HM: Yes what you think that if this is done it can help to reduce the problems that are encountered by people because of COVID-19 disease or it can help to reduce the COVID-19 disease

RES: Ahh in my view I can say that they would make everyone tests for COVID-19 , people shouldn’t be allowed to do gatherings since its still there it has not ended this disease, we don’t know when it will ends, people should try to follow the rules that has been said that we should…so that disease doesn’t spread

HM: Alright so you are saying people should be told how can they be told through radios, should people move around telling them, should they hear on TV people what are they supposed to do?

RES: Broadcast on radios those who move around then move around

HM: So that people can hear that COVID-19 what should we do to reduce?

RES: Yes it’s there we want COVID-19 to end

HM: It there anything else that can be done there measures that can be done or program then come as programs, there’s nothing

RES: Hmm there is nothing

HM: Alright thank you for your time you go and sit where others are you will be given your money after.,,
