# Supplementary material for: “Getting pregnant during COVID-19 was a big risk because getting help from the clinic was not easy”: COVID-19 experiences of women and healthcare providers in Harare, Zimbabwe
Source: PLOS Glob Public Health. 2024 Jan 8;4(1):e0002317. doi: 10.1371/journal.pgph.0002317 (PMC10773929; doi:10.1371/journal.pgph.0002317)
Supplement: S1 Data — (ZIP) [file pgph.0002317.s003.zip › Data/Mothers/Participant 3.docx]

**Interviewee’s Gender: Female**

**Interviewee’s Age: Around 30 years**

**Interviewee’s Initials: Mother 3**

**Length of Interview: 31:43**

CM: We are now starting our discussion, can you tell me a bit about yourself, where you stay, how old you are, are you married, your home where you stay, and do you work?

RES: I stay in XXX at number XXX, XXX, I am married, don’t work

CM: How old are you?

RES: I’m 30 years old

CM: How many people do you stay with at your home, how do you live?

RES: We stay with 4

CM: You and who?

RES: Me and my husband and my children

CM: Can you tell me what you have heard about coronavirus or COVID-19

RES: Ah (laughing) What I heard about COVID-19, I heard that it’s a disease that infects mostly people who are on medications like those who take ARV, those who take sugar tablets, those who have BPs, and old age people that is what I heard about COVID-19.

CM: But what do understand about COVID-19, what do you understand?

RES: Ah I would lie I haven’t understood

CM: What do you know or what do you understand about it?

RES: I might be able to know that COVID-19 is a disease that is like other diseases so a person should always protect yourself

CM: How do you protect yourself?

RES: Ahh

CM: How do you protect yourself from COVID-19 what are you doing to achieve that?

RES: To protect from COVID-19, social distancing, and always wear your mask whether you are at your house, sanitizing always. If I go out, I sanitize.

CM: Okay can you tell me how feel about COVID-19 personally?

RES: Ahh COVID-19 I feel that it’s a disease that is dangerous

CM: Why is it dangerous?

RES: It is a disease that comes with a strong powerful wave

CM: Powerful?

RES: Power to kill in a very short period …….

CM: What else can you say about COVID-19 you said it came with the power to kill what else do you feel about COVID-19?

RES: Ha-ha (laughs)

CM: You are free to say anything

RES: I felt like it’s a disease that has the power to kill in a short period of time

CM: Is there anything that you are doing to protect yourself from COVID-19?

RES: What I am doing to protect myself?

CM: Hmm

RES: Like when COVID-19 started I didn’t go to crowded places, I’m always wearing my mask even when I am at home.

CM: Hmm

RES: I stay with my sanitizer that’s how I protect myself.

CM: What about your family, the family you stay with how are you protecting them?

RES: I stay with my little children they cannot wear masks, but they know that if they go outside to play when they come back, they must wash their hands or sanitize them.

CM: Is there anything else that you are doing to protect your family?

RES: There’s nothing else except not going to gatherings and staying at our house.

CM: Okay in your view how do you think health workers, nurses, and community health workers are seeing this disease, or the situation of COVID-19?

RES: I think they see it as a challenge because they do not see as many patients as they did before, and they are letting them go.

CM: what do you mean by letting people go?

RES: They cannot serve all the people at the facility. at times do not come to work but people will be sick at times they just come, and they protect themselves by wearing masks.

CM: Okay right now I want to show you about 3 timelines that might be different in your life. And would like you to talk about each stage. It shows when you were pregnant, HIV testing and treatment time, the last one is when you delivered your baby (post-natal)

RES: Hmm

CM: The third one is showing giving birth and breastfeeding, so I want to share your experiences on each stage or what you have encountered on those 3 stages right

RES: Hmm

CM: So I want to ask about, your baby is how many months old?

RES: He/she has 3

CM: He/she has 3 months?

RES: Hmm

CM: Okay I would want to know that in your living on daily basis here we are starting from when you were pregnant that’s here, your living on daily basis, how was your life different before the coming of COVID-19 and when it came when you were pregnant, your activities or things that you were doing daily on your family or social life, be it money and what, what was different?

RES: Hmm it was different

CM: Hmm

RES: When we were living before COVID-19came it was better because we were able to do what we do all the time without limits that we were getting, so when COVID-19started we faced so many challenges that what we were doing

CM: That’s what we came here to discuss so I want you to say that I faced challenges of this and that

RES: We faced challenges on that we were selling, on selling the police was talking things that we were selling 2.there is nowhere to find money so for something to eat sometimes it was difficult, so we faced those challenges during the time of corona to the extent that things were hard

CM: Okay what about on things that you do daily before corona and when corona came is anything that was different is there anything that has changed?

RES: What we were doing on daily basis is that we were people who do their hand jobs so when corona can things were lowering, because sometimes when going to hoard you would arrive maybe the prices might have gone up, what you saw today is not what you are going to see tomorrow, things were changing everyday those were the challenges that we faced during the time of corona

CM: Okay what about in your family and your life your social life of living with others in your community and what, is there anything that changed before corona and when corona happened?

RES: No nothing changed

CM: Nothing changed?

RES: Hmm

CM: What about during the time when you were pregnant on the issue of the programs that you are given here at the clinic, how were you seeing it, is there anything that has changed before corona on your coming to the clinic

RES: Ahh nothing changed, what changed is that the clinic sometimes it was opening late if arrive in the morning sometimes you will be served in the afternoon

CM: Hmm

RES: So those are the challenges that we saw during the time of corona, what we get at the clinic is not what was happening, sometimes you would arrive and there is 1 nurse or no one, this clinic reached a time where it closed because there was no nurses

CM: So can you explain what you encountered from when started to know that you are pregnant until today

RES: What I encountered from when I got pregnant

CM: Hmm

RES: I encountered hardships because things were not flowing

CM: What was the problem what was not flowing? You can speak

RES: The way we were getting our money

CM: When did you know that you were pregnant?

RES: Last year in Mar…in April

CM: We were already in COVID-19

RES: Hmm so from there we had many challenges finances were difficult, if you come to the clinic sometimes who would arrive and it will be closed without nurses the clinic will be closed then you go back home

CM: Hmm

RES: So they are some of the things that were difficult but it’s now better

CM: Hmm

RES: On that we are now seeing the clinic is open if you want to get the child treated or if you want to be treated it’s now better you’re able to get treatment

CM: What about on the issue of being checked and testing

RES: On Testing like me I was tested long back so since I was already on ART I was only coming to collect medication and go back home

CM: Okay what about the issue of breast feeding and giving birth what happened?

RES: When we gave birth nothing happened we just arrived the clinic was open and then we delivered

CM: Were we still in lockdown or?

RES: Yes we were in lockdown

CM: Which lockdown or it has been lifted?

RES: Ahh in December

CM: When did you gave birth to the baby?

RES: On the 18^th^ on December

CM: December the lockdown had been lifted

RES: That’s when I gave birth

CM: Okay that time when you were pregnant …. Okay eh during the time you were pregnant how did COVID-19 affected your mental health, how did COVID-19affected you or your way of living how was it affected?

RES: As for me I was always scared since you would hear that per day this number of people died, this has happened and you are pregnant, you are someone who is on medication you will be thinking a lot

CM: What were you thinking a lot?

RES: On thinking you will be thinking maybe if it’s said that it infects people who are like that, what about me what am I going to do but because most of the time should always protect yourself your mask, you don’t go where people are gathering so I saw that it helped me

CM: Okay during that time when you were pregnant can you explain to me the PMTCT services that you were given here at the clinic or the treatment services that that you were given here you and your child

RES: There are no services that we were given here since they said if you are on Art…

CM: The services to prevent the child from getting infected by the virus when he/she is still in your stomach

RES: Ahhh there is nothing they gave us they said the child cannot get infected because you are already on ART

CM: Okay

RES: So there is nothing that we were given until we gave birth, the baby is the one who was given

CM: Eh after giving birth what happened?

RES: When I gave birth the baby is the one who was given nevirapin to drink the day I delivered up to 6 weeks

CM: Hmm

RES: When he/she reached 6 weeks going with him/her to baby clinic they changed and started to be given cotri

CM: So right now that’s the program he/she is still getting?

RES: Yes

CM: So you said you when did you start to be given the baby’s medication in December when you gave birth?

RES: Baby’s medication yes

CM: All along you were coming here to the clinic to collect your medication?

RES: Yes my medication only

CM: You were collecting from here?

RES: Yes here

CM: Okay can you tell me programs that you can prevent the baby from getting infected with the virus, have you been getting them from this clinic from the onset of coronavirus?

RES: Yes we were getting

CM: Is there a time when you can and did find ehh

RES: Medication

CM: Yes

RES: Ahh no

CM: I would like to ask how you see….what is it called your preparation that you would have done during the time of visiting the clinic and being seen by the nurses, how are those things?

RES: It’s right on that these days we are not able to see them, if we arrive wanting to take your medication your book is taken when you are at the gate then written there, when they come back they give you your book then you go and collect your medication at the pharmacy then you go home

CM: What about other things that are done, that were done to you when you were coming back then, what was happening back them?

RES: What was happening back then is that sometimes is being late

CM: I mean when you were coming to the clinic procedure or how they were doing things, how were they doing things when you have arrived, let’s say today it’s your day of coming to collect your pills

RES: Alright we would stamp my books at the reception then go to OI then I will be given my green book the follow the que of collecting pills

CM: Okay

RES: But because of that people refused that saying that it’s my privacy if I am taking pills, so if everyone sees me holding a green book they will be knowing that I am going to this , so that’s when they stopped, if you stamp your book then you go straight to OI then finish there

CM: Okay so how was your accessing of treatment programs like from the onset of covid?

RES: Ah our treatment programs had never stopped you would…if the clinic is said that its closed we would go to Old XXX it will be open so treatment programs has never stopped

CM: Has you ever arrived here and it was closed when you wanted to take your medication?

RES: Yes

CM: When was that?

RES: Last year in….in May

CM: In May?

RES: Hmm

CM: So at XXX were they giving you medication?

RES: Yes

CM: For how long?

RES: For 3 months

CM: Okay In your community that you stay did COVID-19 affected other people in accessing other treatment programs, we have different treatment programs, some people come to the clinic with different things, do you think coronavirus has affected some people in your community so that they can be able to come and seek their desired treatment programs ?

RES: You would hear some saying that I went to the clinic with a sick person and I arrived and the clinic it was closed, I arrived at the clinic the nurses were there but they were not treating

CM: So do you see that it affected or?

RES: It affected a lot on other people they were affected

CM: Do you think that the information that you…do you think you had enough information that is needed during the time of lockdown the knowledge that a person is supposed to have the knowledge about COVID-19, do you think you had it during the time of lockdown?

RES: Haa I didn’t have much

CM: You didn’t have much information?

RES: Hmm

CM: Okay did you have the information on where to go for your checkups when were you pregnant or delivery during the time of lockdown

RES: Hmm you would …when this clinic was closed when I was pregnant it became hard for me that so when If want to deliver where will I go because most people who were about to give birth they would look for someone to deliver them at homes, there so many people who were delivered by people at homes because the clinic was closed

CM: Hmm

RES: Sometimes if you had money you go to private but when I delivered it had opened, I came and gave birth here

CM: There are how many people who gave birth in homes?

RES: Ah they are many

CM: You gave birth in homes?

RES: Yes

CM: Okay you can say they are many so were you aware of where they were going for delivering?

RES: A person would say I have a nurse that I know so I think they were communicating then a person goes to deliver and go back to their homes

CM: Okay did you know or did you have the knowledge or information about how can you be able to come to the clinic also maybe travelling during the time of lockdown

RES: We had the information because we stay near the clinic so we would walk

CM: You would walk to come to the clinic?

RES: Hmm

CM: Okay did you have the information of what to do when you arrive at the clinic?

RES: Information…if I have arrived at the clinic wanting to..?

CM: Wanting to get help when you have come to the clinic did you know what to do at the clinic from point A point B to where you want to go

RES: Alright You know that when you are going to the clinic wanting get help during this time of covid, you should have a mask, you don’t enter the if you are not taken temperature, you don’t enter the gate if you are not sanitized, if you get inside you will sit according to social distance until you go where you want to go

CM: Okay are there things that has changed that you saw at the clinic from the onset of coronavirus including time of waiting, also type of services that you were being given or the quality of services is there anything else that has changed from the onset of covid?

RES: The difference now is that, if you come here, like some of us who are on ART, if you come you are told to wait at the gate, so we do not want to be seen by many people, but the way they are doing it nowadays makes it so difficult for us, everyone on the queue get to know about our statuses… We stand in the same queue but when they come to collect cards, they say that those who are on ART please hand in your cards and then they will call us one by one. I once left without getting served as my sister-in-law was also in the queue…

CM: Before what were you doing?

RES: We would go to the OI department and no one would know why I had come to the clinic

CM: Okay what about the quality of the services that you are getting how are they?

RES: The services that we are getting they are okay

CM: It’s the same?

RES: Yes they are the same

CM: Okay now we want to talk about issues at home ehh have you ever had problems at your home for you to come to the clinic during the time of lockdown

RES: No I didn’t have any problems

CM: You were coming every time you were supposed to come during the time of lockdown?

RES: Yes I was coming

CM: Have ever had problems on talking your medication of giving your child medication during the time of lockdown?

RES: I have never had problems

CM: Okay do you think….do you think the following things that we call gender dynamics or the difference between me and women and other, how did it affect or I can say how did it affect women in accessing programs to prevent children so that they don’t infected with the virus whilst they are still in the stomach be it in your family or in the community, the burden that the parent or them being supposed to take care of the children and family, did it affect then during the time of lockdown we see that during the time of lockdown many children were at home , if the parent has 4 children all of them were at home, did it affect the parent for them to be able to collect medication to prevent the baby from getting infected by the virus whilst they are still in the stomach

RES: Sometimes we can some might have been affected because some didn’t know if they find that the clinic is closed they didn’t know where to go, since this clinic was closed 2 times

CM: Hmm

RES: So someone to know that if you go to the next clinic with your book you will be given someone wasn’t aware then they will go and stay at home until the clinic has opened

CM: Did it affected their access and control of resources or their access and control of getting money or anything that is needed at home during the time of lockdown, access of money and other things that are needed at home did it affect them making them fail to go and seek treatment programs to prevent children from getting infected by HIV whilst they are still in the stomach

RES: Ahh it didn’t

CM: Okay during lockdown decision making at home and in the community did it affected women for them to be able to go and seek the PMTCT services

RES: No it didn’t affect

CM: Is there anything that you know that has anything to do with child’s health that was affected during the time of COVID-19?

RES: Hmm there is nothing

CM: Things that you see that this is not standing well ehh pertaining your child’s health because of COVID-19

RES: There nothing

CM: Okay the government of Zimbabwe when COVID-19 happened it changed other things, it changed the issues of social isolation that a person you are supposed to stay alone if you have travelled, it changed travel restrictions they no longer allow people to move from this city to that city, it closed schools it closed borders, how do you think this affected other women in your community

RES: It affected some because others where cross borders that was their job that’s if they cross they buy then come here and sell, some would go and sell in rural area where and where, maybe that’s where they were getting something to help themselves so it affected a lot

CM: Okay what about the issue of road blocks how did it affects let’s say you have arrived at the roadblock you want to go and collect your medication

RES: The issue of road block affected because during the time of lockdown the clinic they were saying that they take the last book at 9 o’clock or past 9 sometimes you have been stopped by the road block you will arrive here around 12, the people at the clinic will not understand that you were in a kombi or you were in a car coming

CM: What about the issue of that we have arrived at the roadblock, where are you going, where are you going

RES: I will show them my book then I show that that I am going to collect medication

CM: But did you see it that you are showing your books were there are other people how did you saw it?

RES: They are things that were not fine because many people now know my status

CM: Hmm

RES: It’s some of the things that affected many people or made people has their status known because they would be wanting to go

CM: Okay on the way living in the community and child care did your community talk …did your community changed from the time of COVID-19to …from before the beginning of COVID-19to the onset of COVID-19the way you were living in the community and child care

RES: Hmm it didn’t change

CM: It didn’t change?

RES: Yes

CM: COVID-19 can it be something that…..something that troubles you to what extent in the area, or it’s the thing that need to be looked at to what extent in the community to be looked at

RES: It a thing that we are supposed to stay looking forward to it because people came from different backgrounds, you don’t know where the person is coming from, how is where he/she is coming from, so it’s a thing that we should stay looking forward to it and also you must protect yourself

CM: How do people in this community feel about seeking treatment programs from the hospitals and clinics?

RES: Hmm Aah

CM: How do they see it seeking treatment programs from the hospitals and clinics?

RES: People want to seek treatment but because of that now you can see that a person might be sick but they can’t come to the clinic because of the services that are given at the clinic

CM: What services are being given that makes them not come to the clinic?

RES: Services like especially like I have come maybe I am sick sometimes I’m not sick of sick, they can say waiting maybe you are the people who has covid, then do whatever they will be doing inside maybe I’m sick I’m outside the gate and the time is moving, being attended these days its low here at the clinic

CM: Okay what can be done or that needs to be done so that we can reduce the problems or the negative impacts that has been brought by COVID-19 in your area?

RES: Ahhh

CM: You said hunger you said people were no longer working you talking about that some were disclosing at roadblocks that my status is like this because they wanted to go and take medication, so what can be done to reduce negative impacts that were brought by COVID-19 in your community

RES: What can be needed to reduce these problems maybe if the fathers can return to work, working normal hours that they were working, if the mothers can do the business that they were doing maybe it will reduce the impacts of COVID-19

CM: Is there anything that needs to be done /

RES: What needs to be done is that roadblocks they must be limited so that people can be able to travel

CM: What else?

RES: Because here at the clinic they must attend people who came sick then a person goes home, because sometimes the crowding that we will be doing so we will not be knowing that the person who is sick is sick of what, because they keep us crowding even if its outside the gate they keep us crowding outside the gate if its COVID-19I have been infected because we were crowded on one place

CM: Okay is there anything else that you want them to be done or that can be done

RES: They must open the clinic normal hours that they were always open all the time, the nurses must come to work like the way they were doing all this time

CM: Okay do you still have anything else?

RES: There is nothing else

CM: We thank you for the good discussion
