# Supplementary material for: “Getting pregnant during COVID-19 was a big risk because getting help from the clinic was not easy”: COVID-19 experiences of women and healthcare providers in Harare, Zimbabwe
Source: PLOS Glob Public Health. 2024 Jan 8;4(1):e0002317. doi: 10.1371/journal.pgph.0002317 (PMC10773929; doi:10.1371/journal.pgph.0002317)
Supplement: S1 Data — (ZIP) [file pgph.0002317.s003.zip › Data/Mothers/Participant 6.docx]

**Interviewee’s Gender: Female**

**Interviewee’s Age: Around 24 years**

**Interviewee’s Initials: Mother 6**

**Length of Interview: 29:35**

ZM: First can you please tell me how old you are, are you married, where you stay, how big your family is?

RES: I’m XXX years old, I am married I live in XXX in XXX area, my eldest child is 12 years, the younger one is 9 years, and the youngest one is 6 months.

ZM: All right can you please raise your mask so that it can cover your nose properly? Do you work?

RES: I don’t work

ZM: What about your husband?

RES: My husband doesn’t work right now he is at home. He is sick he has TB.

ZM: All right he is seated how long has he been seated at home?

RES: It's now 8 years

ZM: Where was he working?

RES: He is a roasted corn and vegetables vendor, selling vegetables and tomatoes

ZM: All right so how are you surviving now?

RES: Now I am being helped by my brother he stays in Chishawasha he’s the one who helps me with maize meal and vegetables

ZM: What work does your brother do?

RES: He is a soldier

ZM: All right okay besides your brother are there other people who help you?

RES: Ahh no there’s no one.

ZM: Is there anything that you have heard about coronavirus disease?

RES: I can say my special relative got infected with coronavirus and he/she passed so those are things that hurt me. I can say 3 of my relatives were infected by this corona disease they both passed away.

ZM: Who are they to you those people who were infected with corona?

RES: My aunt’s son and my brother

ZM: Your biological brother?

RES: No from my father’s side of the family

ZM: hmm

RES: And my aunt my father’s sister

ZM: Alright where were these people staying?

RES: They were staying in Mutare

ZM: All 3 of them?

RES: Yes

ZM: What else have you heard about coronavirus or what do you know about coronavirus?

RES: What I know about coronavirus disease is that if you get infected by this disease you don’t survive, you will have difficulty breathing some say coughing, and some say high temperature.

ZM: All right, how do you feel about the fact that you are living in the era of Coronavirus or the time of coronavirus which is troubling the whole world. Personally, how do you feel?

RES: I felt touched so much when I got pregnant. I used to come here with up until COVID came.

ZM: Hmm

RES: When I was accompanied coming to deliver. I delivered at the gate the nurses were saying we can’t…help you here. Who told you that we are delivering here go and look for midwives that’s what touches me so much about the COVID-19 thing.

ZM: What made you deliver at gate?

RES: What made me deliver at the gate is the clinic was closed and there were no nurses who were working that is’ why I delivered at the gate. I did not have the money to go to private/traditional midwives since my husband was sick.

ZM: How much was it to be assisted by the midwife?

RES: It was 15 USD

ZM: All right okay had you registered for delivering here?

RES: Yes, I registered

ZM: All right when you registered did you ever have the challenge of coming to the clinic and finding the clinic was closed?

RES: Yes, getting pregnant was a big risk because getting the help from the clinic was not easy. When I came to register the pregnancy, I was turned away and only managed to register on the third attempt. After registering I was told to come back when I am due or if there is a serious need. They stopped all the other monthly ANC visits and even if you showed up for general consultation the nurses would just tell you to go back home so we missed some of the services

ZM: All right when you registered how old was your pregnancy?

RES: I registered when it was 3 months.

ZM: Were you attending maternity scale days when you were pregnant?

RES: Yes, we would come for scale and we would be told we are not scaling then we would go to XXX clinic that’s where we were scaled.

ZM: At XXX were they accepting people who had registered here

RES: Yes they were accepting if you have you’re your card they will scale you

ZM: Alright why is it they were scaling there but here they were saying that they are closed

RES: I don’t how they were doing it here, there they were vaccinating even children if you give birth to a baby BCG they were injecting but here they will be saying that the clinic is closed

ZM: Alright looking at the issue when they started talking about the issue corona virus, is there that you have changed at your home personally or on your children as your family in trying to prevent yourselves from corona virus disease

RES: Hmm

ZM: What Changed?

RES: When we were told that corona disease has come, we were using what are called sanitizers, and boiling warm water and drink warm water and lemons

ZM: Hmm

RES: And what is it called we would go to the fields to take what is called zumbani and we would boil and drink with the children

ZM: Alright what about on your way of living is there anything that changed with your neighbors or with your children forbidding them to go out of the gate, is there anything that you changed in trying to reduce your chances of getting infected with corona virus

RES: Yes to children like the father would say that there’s no child who gets out of this gate because if you go outside you will bring us the disease so we would stay with our gate locked so that the children will not go out

ZM: What about you as mothers were you not talking over the fence with your colleagues?

RES: No at our house you are not allowed to talk to colleagues because the men will say that they will influence

ZM: He said they will influence you to do what?

RES: I don’t know their thoughts, I don’t what their brains will be thinking so we always stay indoors seated at home because right now at our house I’m the one who stays there, so stay inside the gate I am not allowed to go out of the gate

ZM: With your husband?

RES: With my husband

ZM: He says why?

RES: He doesn’t want

ZM: Why doesn’t he want?

RES: I don’t know he says that at his house I don’t know the law that is at his house that the daughter-in-law’s must not talk to their next doors

ZM: Alright

RES: We just say good morning and good evening that all then you lock yourself in the gate

ZM: Did it started when you got married or it started during this time of corona?

RES: It started when got married even when my mother in law was still there before she passed on

ZM: They say what will happen if you talk to them?

RES: They said they will influence you

ZM: To do what?

RES: To do mischievous

ZM: Why are they scared that you will get influenced what had happened?

RES: I don’t that’s how men are

ZM: Alright looking at the issue of this corona disease can you say how are the nurses accepting or how are they perceiving this disease

RES: I can say the nurses here they accept they accepts us well

ZM: Raise it like this so that it doesn’t keep on…

RES: They welcome us well

ZM: But how are they perceiving this disease are they showing that there something they are doing in trying to protect people who come for treatment here or are they afraid or they are not afraid how are they perceiving this disease

RES: No the nurses are able to accept it because they stand outside the gate like the grandfather that is here will be saying no one enters the gate without being sanitized Without washing hands then they get sanitized then taken that thing the thermometer that they do then you enter after being sanitized

ZM: Alright what about looking at the time when you were pregnant till the time you gave birth, you said you would come for scale and you arrive here it will be closed and you go somewhere, you gave birth at the gate, I want you to give me a narration of how it goes from when you left your house coming up to giving birth at the gate

RES: What I did I left home after I left you we arrived at the gate I was accompanied by my sister in law, when we arrived at the my aunt pressed the sirene button then it ringed

ZM: Hmm

RES: Then they came and said…..

ZM: What time was it was it in the evening or?

RES: It was at 10

ZM: 10 what time?

RES: In the morning

ZM: Hmmm

RES: Then they said you came here is everything okay then my sister in law said we came with a person who’s in labor, they said who told that we are delivering here

ZM: Hmm

RES: Then they locked the gate and said get out and go we don’t want to hear nonsense then my sister in law said if you are telling us to get out and go where do we go with this person’s situation, they said we I don’t know that’s up to you

ZM: Hmm

RES: Then my sister in law ran to take our grandmother who stays in Mukanaga so that she can takes us to the midwife from her church they go Mugodhi church (apostolic church) it is said that they do for free

ZM: Hmm

RES: So the time my sister in law left so that she can go there before she arrives at grandmothers the amniotic sac busted and the baby came out that time

ZM: Who were you with?

RES: I was with my grandchild so the amniotic sac busted and the baby came out, what my grandchild was able to do was to take a cloth and spread it I was kneeling like this,

ZM: Hmm

RES: The baby came out my grandchild wrapped the baby

ZM: The baby did head/hit the ground?

RES: No he/she didn’t

ZM: What did he/she do?

RES: Caught him/her with my hands

ZM: You are the one who caught him/her alone

RES: Yes

ZM: Then what did you do what happened after you have delivered at the gate

RES: After delivering at the gate that’s when my grandmother said no we can’t ran with the person to a midwife whilst the nurses are there here

ZM: Hmm

RES: If it’s that the nurses we are going to report them to the police so that they can help the person, so that time there was a guard he is my husband’s best friend thus when he said sister in law has have delivered at the gate then they said yes she was told to go to a midwife

ZM: Hmm

RES: So that guard ran and talked to the nurses here this other grandmother I forgot her name that’s when she came and said ah sorry daughter in-law I didnt know it’s you, you should have called me

ZM: Hmm

RES: To tell me my daughter-in-law was in labor would have come and delivered her then she took me after she took me I was put on a wheel chair then they entered with me, then I arrived they checked the baby they took the baby make up the baby and cut the umbilical cord

ZM: Hmm

RES: They took me i had cracked

ZM: Where

RES: Hmm I had cracked for stiches, they sewed the stitch, and before they sew the water bag was refusing to come out because it had shot so it was refusing to come out that’s when they pressed my tummy with hot water and water bag came out

ZM: Hmm

RES: Then the sewed the stiches they I stayed, after I stayed here they wanted 25 USD they said your person cannot be discharged without paying 25 USD

ZM: Hmm

RES: The father said no that when he gave me his card, the father came walking slowly the he brought his cards of TB that when I got discharged but haa they were reusing saying I won’t get discharged without paying 25 USD

ZM: So you were discharged the same day that you delivered

RES: Yes

ZM: Was the baby given the medication for him/her to take that one that is given to children that are born from pregnant mothers who are on ART

RES: Yes they gave me

ZM: They gave you?

RES: Hmmm

ZM: Alright was the baby checked to taken blood to that he/she didn’t not get infected with HIV?

RES: He/she has not yet tested

ZM: He/she have not yet tested how old is your baby?

RES: He/she has 6 months

ZM: He/she has 6 months but they haven’t taken?

RES: Yes

ZM: What did they say here?

RES: They said you have to come back so now the father had said we should go to satellite at XXX and get her/him tested there tomorrow to see how he/she is

ZM: What about all along from when the baby reached 6 weeks why hasn’t he/she tested?

RES: We went and they said he/she is still young

ZM: Didn’t they tell you to come back when the baby has reached 6 weeks?

RES: They didn’t

ZM: What did they say?

RES: They just said come back

ZM: When?

RES: Tomorrow

ZM: Did you go back the following day?

RES: No tomorrow that’s when I am going with him/her

ZM: That’s when you are going with him/her

RES: Yes

ZM: You said your baby is how old?

RES: He/she has 6 weeks he/she has 6 months

ZM: 6 months or 6 weeks

RES: 6 months

ZM: Alright so your baby was supposed to have been tested at 6 weeks that’s what I want to understand that why wasn’t she/he tested at 6 weeks when she was supposed to be tested

RES: Alright we didn’t understand each other on that

ZM: Hmm

RES: When they said I should come back when I gave when they said I should come back with her/him after 6 weeks

ZM: Hmm

RES: I came back with him/her with my aunt my husband’s sister when we came back with him/her they tested him/her the baby is okay

ZM: Okay then they said stop

RES: Come back with him/her again to see how he/she is

ZM: Alright, what about the medication that is given to children was your baby still given

RES: Yes

ZM: Alright, looking at that you delivered outside did it affected anything on you your brains, your living the fact that you delivered alone

RES: No on giving birth alone I wasn’t touched that much because I knew that that’s how it is there is corona, so the nurses there’s nothing they can do if they are told that the clinic is closed it will be closed, so I want touched that much

ZM: Hmmm

RES: What touched me a lot was that my sister in law my husband’s brother I can say there are 2 I am the youngest the words that she spread to people that’s what touches me

ZM: Hmm

RES: They say that when I got pregnant with this baby she goes around telling people that…I can say at home where I at home they stigmatize me they say even to the children they say don’t the food that is cooked by Mama Taku because she is HIV positive

ZM: Alright who told your sister in law?

RES: When I was tested I can say when I got sick I came with my sister in law she the one who was travelling with me, because my relatives my mother passed away, my father passed away who I call my relative is my brother who stays in Chishawasha

ZM: Hmm

RES: So my brother said I can’t come since you have said that Patience is sick he was at work in Beit bridge so for me to come I can’t I was asking the sister laws that are there to travel with her

ZM: Hmm

RES: Then I will come and see you I am begging so my sister in-law said it doesn’t matter let me travel with her since

ZM: What about your husband where was he?

RES: My husband was there

ZM: Hmm

RES: My husband was following us she said brother in law follow behind us as I walk with sister in law

ZM: Hmm

RES: So when she knew my results that’s when she started spreading

ZM: Alright okay so have you ever talked to your sister in-law showing her that you are not liking it

RES: Yes I told her

ZM: What did she say?

RES: She said mother of Taku I’m sorry I didn’t say it’s an issue then I said no if it’s that you came with me you should have left me to come alone I was able to take my results alone

ZM: Hmm

RES: And for a person to be tested positive it doesn’t mean I have decayed I have never decayed, and it is said if you laugh at a person today tomorrow it’s you, you will need help sister in-law from me you will want to be helped with me so don’t say that

ZM: How did she take it?

RES: She said mother of Taku I have heard you and she even cried begging for forgiveness, the I said no I can forgive you but that’s up to you with your God it doesn’t matter you have been walking talking about me

ZM: Okay from when you gave birth can you that there was a time when the baby failed to be scaled or done other things that are supposed to be done to him/her because of the issue that there was corona virus pandemic

RES: No everything is done

ZM: Does he/she have all his/her baby cards that he/she is supposed to be given

RES: Yes he/she have

ZM: BCG was your baby injected?

RES: Yes he /she was injected

ZM: Okay during the time of lockdown can you has you had all the information about how pregnant women can travel if they are going to scale and what happened

RES: I don’t have information about that

ZM: You didn’t have information on that

RES: Yes

ZM: What about the time you were pregnant did you know what was needed so that you can be able to travel to go to the clinic

RES: Uh

ZM: You didn’t know

RES: I didn’t know

ZM: Alright ever since corona started can you say there’s change you have seen between the number of people who go to seek help at the clinic or hospitals

RES: Ahh no

ZM: What about how services are being given at the clinic can you say there’s any change since the onset of corona

RES: Yes right now when corona started like what they used to do back then and what they are doing now it’s different you can see that there’s a change

ZM: The change is on what?

RES: The change is on that if you come let’s say it’s me who is sick then i come to the clinic to get treatment they are serving us fast and on time

ZM: Alright its different from what was happening back them

RES: Its different from what was happening back then that if we arrive here back then if we arrive here they would say get out ad sit outside but now as an individual you can see that there is a change, that if you come with a sick person they will call you in a short period of time they will serve you in a short period of time then you go out and go back home with your patient

ZM: Alright looking at this disease and the community that you stay in can you say covid affected people’s lives in the community pertaining getting treatment services

RES: No

ZM: What about people who are on ART what were they doing during the time when the closed was closed

RES: Time of…people who are sick if they would come to the clinic we could be told to pay money for card stamping they could say for us our ART medication they could say pay dollar

ZM: Hmm

RES: So that you can be given medication if you don’t have a dollar they would tell to go back home

ZM: Hmmm

RES: So those are some of the things that hurt us a lot that if you don’t have a dollar should you die?

ZM: Alright okay, what about other people in the community were they able to come and get treatment or they were failing to get services because of corona?

RES: They were even failing to get treatment because of corona

ZM: Alright did people encounter problems that has nothing to do with treatment at homes because of corona disease, do you think people encountered challenges and what are the challenges that were encountered by people in their homes because the father was at home, the mother was at home people were seated

RES: We can talk about at homes, jobs men corona caused their jobs to end. Jobs ended children couldn’t get food because the father was the one who was going to look for food

ZM: Hmm

RES: So we would when going or men are going to sell the things would be taken by the police and we will be left on zero

ZM: Police was taking on what basis?

RES: The stuff was taken by the police the moment you start to set up the police would come the came and take, police didn’t what people to crowd and sell those are things that drawled us back that our husbands we survive by selling our husbands going to stand there and sell but you would hear that the police came and took

ZM: Alright what about the issue of fighting in homes, gender based violence, was there any disturbances happen on the way people were living at homes because there was corona disease

RES: If it’s the issue of gender based violence don’t say, people were fighting like where I stay our next door a person was beaten because of food

ZM: What made people fight?

RES: If they can’t find food at home the wife will shout that go and work, where I will work when jobs had closed

ZM: What about on the issue of talking about HIV do you think there are other homes that had GBV that maybe a person was taking medication without revealing his or her status but now because everyone is at home you are looking at each other

RES: Now I can say there is someone we buried it is yesterday at XXX

ZM: At what?

RES: At XXX graves the husband was taking hiding from the wife, the husband was talking and hiding from the wife so for the wife to know that his husband was taking they came together the wife was seriously ill

ZM: Hmm

RES: Then came together and got tested the husband before he was tested that when the grandfather said to him why were you hiding, who is this to you then he said that’s my wife so he said why were you hiding to your wife whilst you were coming to collect medication here what’s embarrassing

ZM: Hmm

RES: Then he said grandfather you have exposed me he drank poison and he died

ZM: Who drank poison?

RES: The husband he drank poison and he died why have you exposed me

ZM: What about the wife what did she do, how is she?

RES: The wife now is better she is better

ZM: Alright what about looking…

RES: She’s now able to do works

ZM: Alright what about looking at the issue of giving children medication do you think women faced challenges on how they can give children medication, those women who gives children medication on their own without reviling the to their husbands

RES: Ah that of not reviling the truth to your husband is a bad thing because you ….let’s say Ruva has been caught then I hide to the father it doesn’t makes sense

ZM: But there are people who are like that I am saying during lockdown do you think they faced challenges?

RES: Ah I don’t think so

ZM: Alright looking at your child’s health is there anything that you are fearing for as child who was born during the time of lockdown and also as a child who was born outside is there anything that you are fearing for?

RES: Not there’s is nothing I am fearing for because my baby me alone I can see that my baby is healthy

ZM: Alright what about you on your health as a mother?

RES: On my health since I’m on ART I can see that my life is now okay

ZM: Hmm Alright

RES: I am now able to take care of my children

ZM: Alright our government implemented many measures in trying to reduce that people should not travel so that they don’t get infected with corona virus, measures like if are infected with the disease quarantine yourself stay on your own, if you’re suspecting that you have the disease do what is called self-isolation that you will be on your own, people should not travel stay in you homes looking at the community that you come from, do you think these thing can affect or can it be done at homes ,that if a person falls must stay alone not mingling with other people

RES: That cannot happen because some get touched that you are putting me in my own room what have I done

ZM: Yes you explain to him that it’s because you have been infected by this disease

RES: Hmmm

ZM: What about the issue that people are told that they must seat spaced and not crowding in the area that you stay do you think it’s feasible

RES: In the community that I stay in some are… some they don’t listen but others I saw they understand they space but others who doesn’t understand they seat close to each other

ZM: These laws that were implemented by the government that people doesn’t travel and what do you think there anything that it effects on you, you as women or other women that are in your community

RES: Yes it doesn’t affect us because that is what supposed to happen because there is corona every one every woman is seeing that there is a disease

ZM: So do you like it that the government says people should not travel, stay in your homes don’t go out don’t go anywhere

RES: Yes we like it because we are helping saving our lives

ZM: Alright what about looking at the issue that the police implemented roadblocks saying we want people who has letters to travel do you think it affected you to collect your medication or to collect mediation for the baby as a mother who is living with HIV

RES: On that I can say they affected us because if they say go back home to travel with letters maybe I might not have the letter but I want to go to the clinic to collect medication for the baby and for myself

ZM: So what can be done in sure like on that?

RES: (The bay was crying) In future the police must understand that if I don’t have a letter but I want to go and collect medication

ZM: Hhmm

RES: They must understand my card they must understand it that that’s what is there

ZM: Do people feel free let’s say you are in a bus to take out their card to show that that’s the one I want to use that’s what I am travelling for

RES: Haa on that no I take him/her then we go aside then I tell him or her

ZM: Can you say people feel free to do that or would not be wanting even the police to know

RES: Some would not even want to understand that will be a bit problematic

ZM: Alright what do you think can be done by our government so that the mothers with children can continue to be able to access services so that the mothers doesn’t give birth outside, the mothers must find or arrive at the clinic closed, what can be done let’s say in future there is another disease like this can should be done so that the children can continue to get treatment and the mothers getting treatment and accessing services

RES: We would be saying as government we would want that if we come at the clinic they must not return us home that go back home because there is a disease no they must accept us well

ZM: What can we do so that we don’t reach to a point of closing clinic as the government what is supposed to be done?

RES: We must listen to what have been said by the government we must listen to it as women

ZM: Alright is there anything else that you think our government can fix so that the clinic cannot close

RES: Hmm there’s nothing

ZM: Alright thank you mother those are all the questions that I had
