# Supplementary material for: “Getting pregnant during COVID-19 was a big risk because getting help from the clinic was not easy”: COVID-19 experiences of women and healthcare providers in Harare, Zimbabwe
Source: PLOS Glob Public Health. 2024 Jan 8;4(1):e0002317. doi: 10.1371/journal.pgph.0002317 (PMC10773929; doi:10.1371/journal.pgph.0002317)
Supplement: S1 Data — (ZIP) [file pgph.0002317.s003.zip › Data/Mothers/participants 19.docx]

**Interviewee’s Gender: Female**

**Interviewee’s Age: Around 27 years**

**Interviewee’s Initials: Mother _ _ _**

**Length of Interview: 29:35**

CM: We are now starting our discussion as I have explained, I want you to tell me about yourself, how old are you, where do you stay, are you married, whom do you live with, and where you work

RES: All right my name is XXX, I’m 27 years I stay in XXX I stay with my husband

CM: How many people do you stay with?

RES: We are 5

CM: You and who?

RES: Me and my husband and 3 children

CM: Okay what about work?

RES: No, I don’t work

CM: Can you tell me what you have heard about COVID-19 or coronavirus?

RES: Yes, I have heard it is a disease that travels through air, it can be transmitted so people should stay wearing masks and sanitizing, you are not supposed to touch your mouth, you are not supposed to touch your nose, or eyes and always have social distance.

CM: From what you heard, what do you understand about coronavirus what is it?

RES: What do I understand?

CM: Hmm what do you understand about coronavirus?

RES: What I understand the most is that it’s a disease that is transmittable

CM: It’s a disease that is transmittable and what else do you understand, it's a disease that is transmittable how?

RES: It’s a disease that travels through the air

CM: Hmm what else?

RES: If we are coughing with must cover our mouths

CM: Is there anything that you have understood about this disease?

RES: Yes, if you have a high temperature and you have symptoms that you are not familiar with or feeling dizzy you call on 2019.

CM: Hmm

RES: Or to go to your nearest clinic and get help there.

CM: Okay can you explain to me how you feel personally about the disease how do you feel?

RES: This disease is painful it has affected people on many levels we don’t go to work, but we do informal jobs, so it has affected us, we are no longer able to work like the way we used to work

CM: Hmm

RES: Before the coming of coronavirus

CM: You said it has affected many things you talked about informal jobs what else has it affected?

RES: Children’s education because of the closure of schools and even getting treatment at the clinics.

CM: What has changed in getting treatment in the clinics?

RES: Sometimes you…the time that we used to arrive at the clinic and be attended to its different from what is happening it now has time.

CM: What is happening?

RES: We now stand in a queue and keep on waiting outside then you have your temperature taken after you are allowed into the clinic premises, and then you will be treated which is time-consuming because some will be rushing to work.

CM: Okay is there anything else that you see that has changed…?

RES: Ahh there’s nothing

CM: Okay is there anything that you are doing so that you can protect yourself and your family?

RES: Teaching one another to sanitize, and mask up.

CM: What about you personally what are you doing?

RES: Us at our home if you arrive, we sanitize hands.

CM: Okay in your view how do the nurses and community health workers, perceive the COVID-19 situation, how are they seeing it?

RES: Corona is hard for everyone it’s hard for everyone because right now most of our relatives have, they are dying because of Coronavirus.

CM: What about the nurses how do you think they perceive this situation?

RES: Aah it is hurting them corona is hurting everyone

CM: Hmm is there anything else?

RES: Hmm there is nothing else

CM: Right now, I want to use the time approach. I have my paper which will show 3 different time stages in your life, it shows when you were pregnant, when you tested and got the HIV testing services when you delivered your baby. I want you to think of the things that happened when you were pregnant.

RES:

RES: Hmm

CM: On the following time I’m showing the stage when you get tested time of being tested, on the following time it shows the stage of giving birth and breastfeeding that you are doing to your child, those are the 3 stages that we want to discuss about now right

RES: Hmmm

CM: Ehh I want to understand that on your daily living is there anything that is different from the time when there was no corona and now, during the time when you were pregnant, when did you give birth?

RES: I gave birth on the 4^th^ of June

CM: 4 June 2019

RES: 2020

CM: 2020 sorry

RES:

RES: Hmm

CM:

CM: I would want to know that in your life is there anything that has changed when you were pregnant from corona getting in corona is there anything that has changed?

RES:

RES: Yes there are somethings that changed

CM:

CM: Hmm

RES:

RES: Like the days I came to register in 2019 that’s when it was said there is corona disease during those days the nurses were on strike, I just registered I didn’t come for scale I started coming to scale 2020, we would come for scale let’s say once, it was different sometimes you would leave without even being scaled

CM: Hmm

RES: When I gave birth, I gave birth on the 4^th^ of June, yes I was helped well I don’t want to lie

CM: Okay is there anything that was different during that time when you gave birth, what about the issue of getting tested is there anything that was different?

RES: No I was tested everything

CM: Okay but was there anything different during the time when there was no COVID-19 and the time when there was COVID-19?

RES: There was nothing because at OI they were open it was working

CM: What about your things that you what do at home daily is there a different that was there during the time when there was covid?

RES: Yes the different was there because I do selling on a market, so I was not able to sell because people were not wanted to be seen in the streets it was said lockdown everyone must stay indoors so it was different

CM: What about the life of the family the way you were living before lockdown and after lockdown, the life of the family and the life of those who are surrounding you

RES: Haa the life was different

CM: Hmm what has changed?

RES: Because what we were eating at first before corona came it was different because during lockdown ighh there was nowhere you could get them, if working we were no longer working

CM: Okay talking about the issue of getting treatment programs how was it in your view or what you have encountered is there anything that has changed when there was no corona and when there was corona when you were coming here to the clinic to get treatment maybe you’ve come to your review dates is there anything that has changed

RES:

RES: What changed is time that they finishing late but at the beginning they were finishing early

CM:

CM: What about when you haven’t …your pregnant were you pregnant during the time when COVID-19 started, when you were pregnant is there anything that has changed?

RES:

RES: Uhh no

CM:

CM: What about during your time of breastfeeding the baby and giving birth is there anything that changed on accessing the treatment program?

RES: On accessing treatment programs there nothing that changed for me

RES:

CM: Okay how has COVID-19 affected your sexual health….your mental health?

CM:

RES: It didn’t affect anything

RES:

CM: If I am saying mental health I will be saying mental health the way you think and how you feel deep down

CM:

RES: Yes it affected me on that I was thinking that is this going to end like for us we depend on market

RES:

CM: Okay have encountered having stress or having depression or anxiety?

CM:

RES: Yes stress you will be thinking a lot

RES:

CM: And thinking deep that you will reach a point of not knowing what to do

RES: Hmm especially when its dawn

CM: What was happening?

RES: When it’s dawn you would start to think that ighh its now dawn what am I going to do, you can’t go to Mbare you can’t go to the prazzeros, what can I do with the children

CM: Okay what about during the time you had given birth and what, during the other lockdown did it happen to you?

RES: Ahh no, what affected me when I gave birth is that I didn’t manage to be given child’s birth record

CM: Hmm

RES: They said we will come and take but up to now I have not yet managed to be given they are always saying we are not yet giving that the only thing that affected me

CM: Okay they said we not yet able to give you?

CM:

RES: Yes last time they said we are not yet giving birth records when we are giving we will tell you but registering I had registered

RES:

CM:

CM: Okay can you tell me about the PMTCT services, you and your child were you able to get these services here at the clinic?

RES:

RES: Yes

CM:

CM: During the time you were pregnant did you manage to get these services?

RES:

RES: Yes

CM:

CM: What about the time when you gave birth did you manage to get these services?

RES:

RES: Hmm

CM: I would want to understand that when did you start doing these services?

RES: When I was pregnant I started in October

CM: Hmm

RES: Up to now I still coming to the clinic with the baby

CM: Have you always been using this clinic?

RES: No

CM: What did you do?

RES: I would come let’s say…you are trying to say using

CM: That where you have always been using be it collecting your medication or treatment that’s where you have been using?

RES: Yes

CM: That’s where you have been using?

RES: Yes

CM: Okay this program of preventing the child from getting infected in mothers stomach, in the stomach you started it when?

RES: I started…when I started to be given pills because I was caught in October when I came to register from then up to now

CM: Can you explain to me the time from when you got pregnant up to now what happened?

RES: Uhh

CM: From the day you started to know that you are pregnant, your journey how did it go?

RES:

RES: What happened is that my husband was in South Africa that where he was working, the job ended after the job ended that’s when he came here in December

CM: hmm

RES: Coming back home in December we stayed and we stayed haa things were hard I don’t want to lie, even the money for me to register…..to buy preparation it wasn’t there, I had registered

CM: Hmm

RES: Until I gave birth things were tough

CM: Okay

RES: Things were tough

CM:

CM: What was tough explain that’s why that we came to a closed place so that you can explain

RES:

RES: What was hard is that the food for me to eat and to give children those were the things that were hard and I was no longer going to the market, the husband was no longer going to work

CM: Okay so when you came to register what happened to you when you arrived at the clinic showing them your cards, I have come to register

RES: When I came to register I….. Ah I was treated well I don’t want to lie, I arrived then I paid my money then I was tested

CM: Ehh

RES: Then I was given my medication then I went home

CM: We were aware of your status already or that’s when you started knowing about your status?

RES:

RES: That’s when I started knowing my status

CM:

CM: When you were pregnant, you came to register when it has how many months?

RES: I registered when it was on 3 months

CM: It was on 3 months?

RES: Yes

CM: Then after testing you have been given your medication what about when you came to give birth what happened?

RES: When I gave birth I was given nevirapin to give the baby

CM: Hmm

RES: Then I went for 6 weeks that’s when they started giving cotri to give the child

CM: Eh can you explain to me that this program of preventing the baby from getting infected with the virus in mother’s stomach, you and your baby did you get them when covid started, did you get from this clinic since the onset of covid, were you always getting from this clinic?

RES: Yes

CM: Was there no a time when you go to another clinic?

RES: No

CM:

CM: How were you seeing it on the time that you have been prescribed to come to the clinic and the time that will be prescribed to come for check ups?

RES:

RES: Sometimes I would feel touched because the time of covid people were told to line up, then they say those who want to go for medication go to this side

CM:

CM: Hmm

RES:

RES: Those who are going for baby scale go to this side so they are things that were hard, like for us it’s our area, we would see that we are being discriminated and the people who will be passing they would know that ohh those are those

CM:

CM: Okay so you were stopped at the gate that’s were they were doing that?

RES:

RES: Yes

CM:

CM: Then when you have entered what do you do?

RES:

RES: Then they…they will come and take the cards after taking the cards they go with them, then they will start to say 3-3 enter inside

CM: Then you take medication?

RES: Then you will be given your medication

CM: Okay what did you encounter in accessing these services preventing the baby from getting infected by the virus, since covid had showed that it was an emergency disease that haa it was hard, what else did encounter in trying to get the services to prevent the baby from getting infected with HIV

RES: Hmm there’s nothing

CM: Nothing, you don’t have any challenges that you encountered or the good things that you encountered?

RES:

RES: No what I can say was good is that when we came to the clinic you were given your medication then you go back home

CM:

CM: What were they doing?

RES:

RES: I mean that’s what was good, that there’s nowhere you could return without being given medication

CM:

CM: Okay during that you would return after given your medication?

RES:

RES: Yes

CM:

CM: In your community where you stay how did coronavirus affected other people so that they can be able to seek treatment programs, without focusing on people’s statuses but how did it affect other people who wanted to come to the clinic to get treatment services?

RES: It affected lot of people because it had labour just to come to the clinic out of the blue, some were afraid of corona that when we go to the clinic you will get infected with corona

CM: Hmm

RES: Because you dont know the person you are meeting with, so some would….it was one of the things that a person can use money to go to the private doctors because of fear on getting infected by this disease

CM: Okay what were others doing, how were others affected?

RES:

RES: Some were no longer coming to the clinic

CM:

CM: Do you think you had all the information that was needed during the time of lockdown?

RES:

RES: Yes it was better we had it, we had little

CM:

CM: Did you know where you were supposed to go during the time you were pregnant to get checked pertaining your pregnancy?

RES:

RES: Yes

CM:

CM: What about of where to give birth or where to get services to prevent the child of getting infected with HIV/AIDS did you know?

RES:

RES: Yes

CM:

CM: What about where….you’re seriously sick you want to deliver did you know where to go?

RES:

RES: Yes we knew that you just go to the clinic they will help you

CM:

CM: Okay did you know how you were able to get to the clinic and also how to get transport to come to the clinic during the time of lockdown/

RES:

RES: Hmm that it was that you would search for yourself

CM:

CM: Hmm

RES: : Hmmm then you see what’s good for you if you are walking on foot then you walk on foot because cars were a problem and cars were expensive, the cars were not carrying everyone

CM:

CM: What about where to go to…did you know what you were supposed to do when you have arrived at the clinic during the time of lockdown

RES:

RES: Yes

CM:

CM: What were you supposed to do?

RES:

RES: If you arrive you were taken temperature first, wearing your mask they sanitized then you enter into the clinic then you will be helped

CM:

CM: Okay is there anything that you saw that has changed at the clinic from the onset of coronavirus including your time of waiting, including types of programs or quality of the services that you are getting?

RES:

RES: What changed is that time has changed sometimes the clinic was being opened at 9 o’clock or to 10 that’s when they would start working

CM: Hmm

RES: Which is that you would have leave home and left children you would go back in nightfall because it will be full everyone wants to be helped

CM: What about quality or types of the services that you were getting how were you seeing it?

RES:

RES: The types were different because people were being said that those who have come to collect medication go to this side, those you’ve come to get treatment only go to this side so it was different

CM:

CM: Hmm

RES:

RES: It was different from back then that we were mixed you personally you will knowing where you are going

CM:

CM: Okay now we want to talk about your life at home or things that happens when you are at home pertaining you living

RES:

RES: Hmm

CM:

CM: Are there any challenges that you have encountered at home pertaining your coming to the clinic during the time of lockdown

RES:

RES: Yes I encountered challenges

CM:

CM: What were the challenges?

RES: The challenges were that when I gave birth I started to have contractions at night to look for a car to come to the clinic it was a problem because the money they were saying uhhh it was a lot

CM:

CM: Hmm

RES:

RES: That’s the only problem that I have encountered

CM:

CM: What about on the issue of HIV status disclosure during the time of lockdown have you encountered challenges?

RES:

RES: Uh no I didn’t

CM:

CM: Okay was your husband tested?

RES:

RES: Yes he was tested

CM:

CM: Okay you were tested together?

RES:

RES: Yes

CM:

CM: What about financial situation how was it, what are the challenges that you encountered besides the money that was charged to come, to get a taxi to bring you to the clinic, what else?

RES:

RES: Money for us are to be able to survive

CM:

CM: Money for you to be able to survive?

RES:

RES: Hmmm

CM:

CM: During this time of lockdown are there challenges that you encountered pertaining collecting your medication or giving your child medication during the time of lockdown?

RES:

RES: Hmm no

CM:

CM: There are no challenges that you encountered?

RES:

RES: Yeah

CM:

CM: In your view do you there are other things that happened pertaining the living of women and men or other things that were affecting women for them to be able to come and collect medication to preventing children from getting infected with HIV in your family of in your community, is there anything that you think that during the time of lockdown was there anything that it was affecting

RES:

RES: Hmm lockdown affected, many people were not used to staying with their husbands near so people were doing GBV

CM:

CM: Hmm

RES:

RES: Which is that if a person is beaten by her husband she will go to her parent’s house leaving baby’s cards

CM:

CM: After leaving baby’s cards then what will happen?

RES:

RES: Then will go with baby to get baby’s medication late

CM:

CM: Sorry I didn’t hear

RES: Will go late to collect the baby’s medication

CM: Going late with the baby to medication?

RES: Yes

CM:

CM: Okay that’s some of the things that affected women not to take what…..

RES:

RES: Yes

CM:

CM: What about child care and the jobs that they have at home it affected then from being able access those services of preventing the baby from getting infected by the virus in in the stomach

RES:

RES: Haa it wasn’t affecting

CM:

CM: What about their access to money and things to use at home was it affecting?

RES:

RES: Yes it was affected because many people it was lockdown

CM:

CM: Hmm

RES:

RES: People were being told to stay in homes so there was nowhere people can get money

CM: Okay what about decision making in their homes and in the community is there anything that it affected on women for them to be able to use these services, decision making at home, what will be said in that house or in the community is there anything that it affected on women

RES:

RES: Ahh no there’s nothing

CM:

CM: There is nothing that it affected?

RES:

RES: Hmm

CM:

CM: Is there anything that you see on living of your child or on your child’s health that was caused by COVID-19?

RES:

RES: Ah there is nothing

CM:

CM: There’s nothing?

RES:

RES: Hmm

CM:

CM: What about in your family/

RES:

RES: Hmm there’s nothing

CM:

CM: Okay here in Zimbabwe we closed we had lockdown, we did what we were calling social isolation that if a person is back from travelling he/she must stay alone or is we suspect there is COVID-19 a person must stay alone, they stopped travelling going to other cities or other provinces, the stopped school from opening, they closed school, we closed borders, how do you think this affected or how much impact did it had on women or other women on you and other women In your area

RES:

RES: It affected a lot of people because many people these days they are doing hand job they work for themselves they were not able even to cross the border because the borders had closed

CM:

CM: What else did it affect?

RES: Even at schools some children you were not able to get money to pay for the children’s extra lessons because some money were coming as hand to mouth

CM: Okay do you think during the time of lockdown the roadblocks that were there, what was being caused by the roadblocks on the issue of saying your status or saying your reason of travelling in the bus or on other people, what was happening?

RES: Yes they wanted to know where you were going and where you are coming from

CM:

CM: Let’s to people like you who wants to go to collect your pills you are at the road what was it doing to people, that you will be then asked where you are going, let’s see your letter

RES:

RES: Haa it was affecting people

CM:

CM: It was affecting in what ways?

RES:

RES: Because you will be seeing like everyone is now knowing that’s what you’re travelling for

CM:

CM: Okay is there anything that it was affecting

RES:

RES: No there is nothing

CM:

CM: What about being discriminated and stigmatized

RES:

RES: Some will start to talk which will affecting you will be thinking whether you should I go or not

CM:

CM: On the issue of living in the community and child care did the community talked about what changed from ….is there anything that changed on the issue of living in the community and child care from before covid started and when covid started

RES:

RES: Yes it changed on that the child right now the child is not able to go for scale to see whether he/she is growing well, you just go to vaccinate the baby only

CM: What else?

RES: And going to the clinic when the baby is that’s what is done only

CM: What about child care is there anything that changed?

RES: Haa there’s nothing

CM: Nothing changed

RES: Hmm

CM: How big is COVID-19 in this community, how much care can we put for COVID-19 in this community?

RES: Haa COVID-19 we are supposed to know that it’s a disease that is there

CM:

CM: What about in this community how do you see it, how much impact does it have or what concern does it have, can we say it’s a big that is affecting your community or?

RES: I can say it’s now better we are not supposed to fear because people now have knowledge about covid

CM: Okay how do people in your area feel on the issue of going to the clinic to seek treatment services and going to polyclinics, is it raining?

RES: Hmm

CM: How do they feel?

RES: What I was saying that some are failing to go to the clinic because they will be shy that I will meet mother of so and so then she will me at que what will she say

CM: Okay is there anything that you are doing that is needed in this community to reduce the problems that were brought by COVID-19 in your community, is there anything that can be done?

RES: What can be done is that people we are supposed to go and collect our medication well and protect ourselves from this covid disease

CM: Okay is there anything else that can be done, that we can do in our community so that this disease of COVID-19 will not affected much in our community

RES:

RES: May water come here in Tafara Mabvuku especially water it’s a problem, if we could be given water the bauzzers, must move around and council must fix those taps that are not working

CM: Is there anything that you think can be don again to reduce the negative impacts of COVID-19?

RES: Ahh we should stay wearing masks

CM: Okay do you have questions or anything that you would want to understand

RES: No I don’t have
