# Supplementary material for: “Getting pregnant during COVID-19 was a big risk because getting help from the clinic was not easy”: COVID-19 experiences of women and healthcare providers in Harare, Zimbabwe
Source: PLOS Glob Public Health. 2024 Jan 8;4(1):e0002317. doi: 10.1371/journal.pgph.0002317 (PMC10773929; doi:10.1371/journal.pgph.0002317)
Supplement: S1 Data — (ZIP) [file pgph.0002317.s003.zip › Data/Nurses/Healthcare Worker 1.docx]

**Interviewee’s Gender: Female**

**Interviewee’s Age: Around 47 years**

**Interviewee’s Initials: HCW**

**Length of Interview: 35:14**

CM: Thank you for accepting to be part of this study to start can you tell me a bit about yourself for example your age marital status, qualifications, and designation

RES: I’m 48 years old 47 turning 48 in October, I am married I have got 3 children I am a midwife

CM: Can you please tell me how you feel personally about COVID-19- 19 or Coronavirus

RES: How I feel about it?

CM: Yes, personally how do you feel about Coronavirus?

RES: I think we were not very affected especially this side in Zimbabwe. I think people here in Zimbabwe are not well informed about corona especially here in XXX. They don’t even know the impact of the virus and some of them think that it does not exist, and it affects the rich only.

CM: Hmm

RES: Such that you can find people without masks in public places and what, but I think it’s real it’s only that we are just fortunate that we didn’t have many deaths as compared to other western countries.

CM: Okay has COVID-19 19 affected your mental health or general wellbeing?

RES: Those days when we had a high number of deaths it really affected me psychologically and especially there was a time when we were tested here and we had staff who tested positive it really affected me I was even afraid of coming to work

CM: Hmm

RES: It really affected me to the extent that I hated coming to work, I was saying it was better to stay at home

CM: When did this happen when were you tested?

RES: The second wave when the second wave happen

CM: Okay is it okay to share the number how many tested positive?

RES: About 22 I think here at XXX out of all the staff

CM: Okay how many staff members do you have here?

RES: Roughly

CM: Hmm

RES: I think we are around 80 so 22 especially the ones I worked with they tested positive, so I was afraid that obviously I am also positive, so it really affected me.

CM: Okay can you please tell me about your portfolio of work or your current assignments what do you?

RES: Me, the work I do at this facility?

CM: Hmm

RES: I am a midwife so basically, we do the delivery stuff, ANC booking, delivery and postnatal care though I’m also a PMTCT focal person for this clinic

CM: Okay you are the focal person for PMTCT?

RES: For this clinic

CM: Okay were there any current reassignments due to COVID-19 maybe the changes that happened where you were reassigned maybe to do something else that you were not doing back then?

RES: Not necessarily with the exception that we were assigned to do a COVID-19 test when people from the Ministry of Health came, we were supposed to be testing, I think about 8 we were assigned to do the COVID-19 testing otherwise, we were doing our normal duties of ANC bookings, delivery, and post-natal care

CM: Okay now we want to talk about the health context in Zimbabwe can you please describe the health context that you work in

RES: Can you rephrase your question

CM: Okay how do you see let’s say the health context, the way you work in the health system are there any concerns about the health care context or health care system that you work in?

RES: I think we don’t really receive the attention that we should receive as healthcare workers I think people look down upon the job that we do they don’t really know what we really do.

CM: Hmm

RES: Because there was a time when people came, they were amazed at what we do. The ambulance technicians were appreciative when we do deliveries, but I think people who are out there don’t know that by 2 am we are awake, and we are dealing with a mother who is delivering who is crying who is in pain and we are wide awake and fully doing our job.

CM: Hmm

RES: And people are sleeping they really don’t appreciate what we do, I think we are not given the recognition that we should receive.

CM: You have mentioned recognition, what other concerns about the healthcare context concerns that you have besides the recognition?

RES: In view of the issue of COVID-19protective wear gear, the personal PPE we are not receiving what we should receive, so I feel that they don’t take the healthcare workers seriously, we don’t receive adequate PPE, salaries, and everything that we will be expecting.

CM: All right, what measures or changes are you making in response to COVID-19 19 maybe on a personal level

RES: On a personal level the measures that I am taking in response to COVID-19 19 is just educating my family on the importance of handwashing, social distancing, and reemphasizing even at church we really do emphasize the importance of hand washing, social distancing wearing of facemasks.

CM: Okay you said about the family what about personally what measures did you take?

RES: Personally, I am doing the same

CM: Okay any other?

RES: Hmm No

CM: Okay so at the organizational level at XXXX Poly Clinic what changes are you making?

RES: In response to COVID-19 19?

CM: Yes

RES: Because initially, people were not taking this thing seriously like wearing facemasks but now you see everyone is wearing facemasks properly people would just wear them just for the sake of wearing a mask even if it’s here at the clinic.

CM: Hmm

RES: But now people are properly wearing face masks, hand washing, and sanitizing people are taking it seriously.

CM: Okay how do you cope with these changes in your life or work?

RES: It’s difficult it’s very difficult especially the wearing of facemasks. it’s difficult because sometimes I feel like it's burning you know, that is life we have accepted what is there.

CM: Hmm

RES: But it’s difficult the handwashing it’s okay we are used to doing that, as nurses we wash our hands always sanitizing it’s okay but the wearing of a facemask and the social distancing sometimes it’s difficult especially when we are in contact with the patient most of the time we listen to the fetus's heartbeat we deliver babies in the labor room so there’s no 1-meter distance we are always with the patient, so sometimes it’s difficult for us but there’s nothing we can do.

CM: Okay in your perspective how are healthcare workers perceiving the situation, the ones that you work with how are they seeing the situation of COVID-19 19?

RES: Now that the deaths have decreased, people now have a better perception than they have, but that time when there were so many deaths when we had the patients who were suspects you would find that people were hesitating to attend to that patient because maybe people didn’t undergo training.

CM: Hmm

RES: Or that people were fearing for their own life but now, people are talking about it just like any other disease you know. They are now taking it like any other disease and taking precautions

CM: Were there any trainings that were done in line with the COVID-19-19?

RES: There were training we had training

CM: In-house training or?

RES: There was no, in-house we had training by the City Health Department, and we would go to be trained in batches I think almost all of us were trained

CM: Okay how has COVID-19-19 impacted the delivery of PMTCT services?

RES: It has really affected the delivery of PMTCT services. As a PMTCT focal person, you find that you meet with a mother who has delivered at home sometimes because of transport and because they couldn’t manage to go to another facility there was a time were closed when we had 22 positive cases of staff,

CM: Hmm

RES: We had to close everyone was in isolation and they had to go to the nearest clinic which was Mbare some of them didn’t have the money to go to Mbare so they would deliver at home. Then what about those positive mothers? You find her maybe accidentally then they tell you this is my baby, or I gave birth 4 weeks ago birth didn’t get nevirapine at birth.

CM: Hmm

RES: She didn’t do birth testing because it is mandatory to do testing at birth, so I assume that most of them who delivered in that period didn’t have the birth testing and they didn’t get their nevirapine, so it really affected the PMTCT delivery of services.

CM: How about the supply chain of the whole PMTCT drugs were you able to cascade or to give the service of the whole PMTCT package? Earlier you said it was disrupted, were there any cases where sometimes you would not give all the drugs?

RES: Before COVID-19 19?

CM: No during COVID-19 19

RES: During COVID-19 before the closure of this clinic, even those who went to hospitals like Edith Mbare Clinic would get those infant diagnoses would get Nevirapine prophylaxis.

CM: Okay

RES: For other cases who got medical attention they were able to get it, but for those who didn’t some of them maybe they don’t know the importance of taking

CM: So those who went to the other facilities that you directed them they got the testing commodities and the medicines

RES: Yes

CM: Okay what about retention in care of both new and old patients

RES: Can you...

CM: Retention in care for them to remain in care for both the old and the new cases did you manage to cascade that?

RES: Yeah, because there is ITECH it really does follow up on those patients so they are doing the follow-up and they are being called and they are coming back, though some could have missed testing at birth they can be done maybe at 6 weeks.

CM: Hmm

RES: Nevirapine maybe they couldn’t get it and if they are fortunate that they did not get sick they can be tracked down as they track down all the positive cases and those who were missed at birth.

CM: You said ITECH

RES: Hmm

CM: So ITECH was doing some other things on your behalf?

RES: Sometimes we work in harmony with ITECH

CM: Okay how about the issues of samples transportation were there any problems?

RES: We don’t have any problems with the sample…because we do the testing here and the samples are done here. They are done here those baby DBS and the viral loads for pregnant mothers are also done here.

CM: Okay you told me that you closed for some time because there were cases of COVID-19 you closed for how long?

RES: I think 2 weeks

CM: Okay so before you closed how was the ability of health care workers to report to work Are there any challenges? And for those who work in the PMTCT department?

RES: Before or…….?

CM: In the COVID-19 era and before you closed

RES: Yeah

CM: What were the challenges?

RES: The challenge was that our employer didn’t provide us with transport. So you had to come on your own and there was no public transport operating.

CM: Hmm

RES: So, you would stand on the road where there’s no public transport and you are afraid of private cars because you might be hijacked, there were no Kombis. Sometimes you would risk for those who stay around this area they had to walk to come to work so even up to now people are walking you know.

CM: Can you tell me the furthest distance that you say the clinic staff is walking from to come to work in terms of the kilometers maybe the furthest one that you know?

RES: Hmmm I don’t know how far it is from Damofalls I don’t know how many kilometers there are

CM: Damofalls?

RES: Yes, I don’t know how many, some were walking during that time they would walk from Damofalls to Eastview and then from Eastview up to here

CM: Okay I’m not sure about the kilometers as well

RES: Also, I’m not sure how many kilometers

CM: But is it very far?

RES: It’s far away

CM: Okay you mentioned something when we were talking about the fear of getting infected with COVID-19 or coronavirus are there any related bottlenecks on that fear in cases of doing PMTCT services?

RES: The fear mostly was because of inadequate protective clothing because people were not well equipped so people would be afraid to attend to those cases.

CM: Okay

RES: It was the issue of inadequate protection because you would get maybe one N95 and maybe 2 surgical masks for the whole day and maybe no PPE wear maybe they were not there by then, we later had them, but people were just scared.

CM: Okay you have talked about lack of adequate PPE material, so when did you get adequate PPE material

RES: I think it’s not even adequate up to now(laughing)

CM: No, you told me that some other time you were working without proper protective wear?

RES: Yeah

CM: So, what were you using those days?

RES: We had surgical masks only

CM: No other protective gowns

RES: We would get but they were inadequate

CM: Okay how does the COVID-19 19 situation compare to your experiences of other disease outbreaks like cholera and typhoid that happened in Zimbabwe?

RES: We had a cholera outbreak

CM: Hmm

RES: Cholera I think people were not very scared as compared to COVID-19 because cholera was a known thing with a known drug that if a patient receives, he/she will be fine.

CM: Hmm

RES: As compared to COVID-19 was a novel virus, it’s a new virus that is not known so you would get this information tomorrow you get new information no one was even sure, even when we went for workshops, we were told that they were not sure about this thing. it’s a new thing and you won’t be surprised tomorrow if you get another information about it, so people were afraid more about COVID-19 as compared to cholera, people were not afraid.

CM: Okay people were not afraid of Cholera, as compared to COVID-19 are there any other issues besides the ones you mentioned

RES: Maybe it’s just fear of the unknown (laughing) fear of the unknown I think

CM: Okay describe the changes to your service provision as a response to the COVID-19 pandemic. Were there any changes you mentioned when you were talking back then, are there any other changes that took place?

RES: Uhh I can’t think of any other

CM: Okay besides what you were saying that some patients were not coming, and you were afraid and you will be in close proximity when examining them

RES: Contact with the patient

CM: So are there no other changes that you did for you to………. respond to the COVID-19

RES: Ahh I can’t think of any other

CM: Okay what challenges do you think mothers encountered in trying to access PMTCT services during the national lockdown

RES: I think…………. I was feeling, for them when I was at home. I was thinking about what they would do if they went into labor because some of them were not booked especially looking at the economy because most of their husbands were not going to work.

CM: Hmm

RES: As they were not going to work, he looked for money to register her pregnancy and suddenly there was no transport for her to come and get helped at a health facility, she has paid for the medical you know for services, but he can’t come with the wife is she manages to come the hospital is closed.

CM: Hmm

RES: The staff went to isolation, or they are under quarantine they have to go to another nearest clinic you know so all the hustles I think they encountered many challenges, I just felt for them.

CM: Okay are there any challenges besides those two of trying to access the services that you have talked about?

RES: Imagine a person has come we are open, she has come you know the laboring woman she wants to cry she wants to scream you are telling her to close her nose and mouth (laughing) close her nose and mouth because we are afraid of the virus,

CM: Okay even in the labor when you are….?

RES: In labor, she was supposed to close her nose and her mouth in labor because we are afraid of the droplet, droplet infection so she is supposed to close her mouth even when crying…...you know so I felt for them that these women are having a terrible time

CM: Oh, okay do you think your patient had all the information they required during the national lockdown, information concerning the services that they are supposed to get maybe of PMTCT did they have all the information?

RES: We tried we tried especially for those who had booked with us we tried maybe for those who had not booked looking at the issue of PMTCT that a person who is at home is pregnant she has not yet booked she doesn’t even know that she is positive so she doesn’t have the information that she wants

CM: Hmm

RES: But to those who booked we gave them on initial booking we gave them all the information they should get pertaining to their pregnancy, the viral load, the delivery, the baby testing everything we gave the information

CM: Okay did they know where to go for PMTCT services during the national lockdown?

RES: Yeah as I have said earlier they knew, before we closed they would come here after we had closed the Municipal police were telling them to go nearest they would go to Edith Mbare Clinic, some would go to Hatcliffe they knew where to go, and for those who had booked I think they had no problem, but those who didn’t book I think they were the ones who encountered problems

CM: All right, so during the lockdown you had given them information?

RES: Uhm

CM: Okay what about how to get to the clinic including the travel requirements did they have any information concerning that?

RES: Yes, we had given at ANC booking we gave them all the information

CM: Okay what type of information do you give them let’s say their pregnancy is due for delivery and they are at home

RES: We told them before they were due, they must have a transportation plan. I am going to travel like this those who have their money we gave them ambulance numbers that you can call or look for the next door who has cars you talk to them for transportation……... you tell them before so that they will know what to do at the onset of labor.

CM: How about handling themselves at the facility did they have the information, or it was just when they arrived that’s when they were told to do this or do that

RES: No our ANC bookings if we are booking people who are pregnant we take longer at the first visit than subsequent visits, we take longer because we actually sit down with them and tell them what they are expected to do, what they are supposed to bring everything we tell we don’t leave out anything they will be well informed

CM: Okay I am talking about those… the first lockdown we did on the 1^st^ of April 2020 and a patient that came in March, she has arrived you talked to her you did the booking then on the 1^st^ of April she was then told that there’s lockdown yet she is supposed to come for another visit, maybe she came to register in December but when she’s coming back she coming for delivery she wants to deliver, like did you have anything to handle themselves, those who booked during the time you told them but those who registered pre COVID time

RES: They come if she books, they come for subsequent visits when they come for subsequent visits, we tell them that there is this outbreak that is where we expect you to do this and this

CM: Hmm

RES: We tell them we don’t only tell them about initial bookings at subsequent we also tell them what is happening and what we are expecting from them we also tell them.

CM: How about those who come in labor?

RES: Those are unbooked ones they are the ones who might have problems, but I think maybe we were doing a good job

CM: Okay have you noticed any changes in the number of patients seeking care since the onset of the COVID-19 pandemic?

RES: Pardon can you come again

CM: Have you noticed any changes in the number of patients, I am talking about the number of people who came seeking help since the onset of COVID-19 at your facility.

RES: Those who were pregnant?

CM: Yes

RES: I think there was no change

CM: There was no change are the numbers still the same and what is the graph like are there any instances they were drops?

RES: I think there were drops

CM: When?

RES: When we closed I think people looked for other options

CM: Okay did the community hear that there were 22 staff members who tested positive for COVID-19?

RES: Yeah, there was audio which was circulating, and it was you know those prophets of doom saying that at XXX clinic there will be nurses who will test positive don’t go there if you go you will …something like that, so I think that audio affected people and we noticed a drop but things are getting back to normal

CM: Okay now we are drifting to social issues at home in your opinion how do you think the following factors affected women’s access and utilization of PMTCT services, HIV status disclosure, how did it affect the women’s access and utilization of PMTCT services?

RES: HIV status discloser?

CM: Hmm I am saying we have the pandemic everyone knows there is COVID-19 so there are women who are there, and they are on medication, so this issue how did it affect women’s access to utilization of PMTCT services for example on the issues of HIV status disclosure how did it affect them

RES: I don’t think it affected…hmm maybe rephrase your question I am not getting what you are trying to say

CM: Okay I am asking what you think on the issue of the following factors how they affected the women’s access and utilization of PMTCT services the issue of HIV status disclosure how did it affect them?

RES: So that they can come to…

CM: So that they can access and utilize PMTCT services

RES: I think they were coming but before lockdown, maybe lockdown because of the pandemic on its own things are normal the clinic was open though people were dealing with emergencies only, so maybe they couldn’t come for HIV testing I don’t know if that’s what you are asking

CM: Hmm then what about after they have opened or uplifted the COVID-19, they are no longer in lockdown

RES: After lockdown yeah people are coming for HIV testing as far as I know

CM: Okay what about childcare roles and responsibilities where they affected

RES: With the epidemic

CM: Hmm

RES: I think they were not affected because people were spending their time at home doing childcare roles

CM: Hmm

RES: I think they were doing well people were doing childcare because if a person was supposed to go to work they were not getting enough time to see their children, I think people had time with their families than before

CM: Okay how about access and control of resources at home?

RES: Haa here there’s a problem on that if they were both self-employed and there’s no money they might have a problem that if they finish what they had, there was nowhere to find money people are not going to work, because most people were not going to work at that time

CM: Hmm

RES: So I think people were really affected

CM: Okay how about decision-making powers at both household and community level

RES: I don’t think it changed anything, if you have given decisions people agree at home that if it’s the father it is said that the father is the head of the house, if he is there, I think he will continue doing his job I don’t think it really changed anything

CM: So it was just normal?

RES: I think it was normal

CM: Okay the Zimbabwean government has implemented major social changes including social distance isolation, travel restriction, and closure of schools and borders what impact do you think they might have on women in your catchment area?

RES: Yeah it affected those who do cross-border businesses I think they were really affected because some are the breadwinners so it really affected some families that the mother was now at home without any income but still others were going mischievously, you could hear that a person has jumped she had gone I don’t know how they were going but I think it really affected many families

CM: Okay how about the closure of schools?

RES: Yeah, the closure of schools really affected because children staying at home caused a lot of challenges you, would find that there were teenage pregnancies for example at our church there were many teenage pregnancies during this closure because children were not going to school so that’s what happened there, maybe it’s another research that you are supposed to do

CM: Okay do you think these measures are feasible, especially in your catchment area here in XXX we were given social isolation, travel restrictions, and closure of schools and borders do you think they are feasible in your catchment?

RES: It’s not what I mentioned earlier what happens here in XXX you will be surprised that is there COVID-19. If you come, you will be surprised to see here and XXX area what is happening people will be standing and not respecting the 1-meter apart rule and the COVID-19 rules.

CM: Hmm

RES: Even facemask-wearing, doesn’t exist here so it’s not feasible I don’t know maybe I was thinking that maybe if people start dying like what was happening in other countries people will………

CM: Will understand

RES: Yeah otherwise…

CM: Were there any positive cases in this area of COVID-19 cases

RES: Yeah we had

CM: How many were they?

RES: I am not quite sure of the number you can get the number at the lab because we were testing here we are still testing.

CM: And the community knew?

RES: They knew but they just thought it was not important even the owner of a butchery at the shops died, but the thing that those who were affluent were the only ones who are dying

CM: Okay what measures or programs are needed to mitigate the negative impacts of COVID-19 within your community that we have talked about that the breadwinners, there were child pregnancies, people were not even aware there was a need for them to wear a mask the negative impact that was caused by COVID-19, what measures do you think are needed what programs do you think are needed? I think you have mentioned that if you do another research on child pregnancy, what else do you think can be done to mitigate these negative impacts?

RES: I don’t know I am even at the loss of words I think the media is the best to always talk on the radio, on TV talking every now and then but still even if it’s being said they don’t understand

CM: Hmm

RES: Maybe if they gathered a few I don’t know can it be done so that they can be aware that this thing is real and it’s there because I don’t know I’m even at the loss of words on what measures can really be put for them to understand.

CM: Okay what do you think are some of the health impacts of COVID-19 including beyond the infection itself?

RES: Beyond the infection

CM: Hmm

RES: Psychologically it’s really traumatizing if you really know the people who were affected those who had husbands who died sudden deaths psychologically it’s traumatizing

CM: Okay what do you think are the socio-economic impacts of COVID-19 short-term and long-term socio-economic impact?

RES: Short term

CM: Hmm

RES: Short term is that things went down jobs because people stopped going to work during the lockdown and, so for the economy to pick up and get back to where it was, for a person to boast if they had used the business capital and finished, they must start over again.

CM: Hmm

RES: Then long term I don’t know if its long term for these unwanted pregnancies that we were talking about, the delinquencies that happened of pregnancies they are some of the effects

CM: What do you recommend that should be done as a national response to the COVID-19 19/coronavirus pandemic what recommendations do you have that are supposed to be done as a response to COVID-19?

RES: They should continue with the awareness programs that’s the only way to awareness at churches, awareness at hospitals. I think these are the programs we should put in place.

CM: Ehh what measures or programs need to be put in place to mitigate the negative impacts of the COVID-19 pandemic within the workplaces and within communities, other measures that can be done besides the ones that you have talked about in workplaces and in the communities

RES: On what I have said I can’t think of any other

CM: Okay thank you so much for the interview do you have any questions or other things that you would like to ask before we finish

RES: I’m not sure when you said we are going to meet again after 6 months will we be going through this or

CM: Yeah, we will be doing interviews as well that maybe post COVID-19 are there any changes what do you see now what is happening now?

RES: Okay

CM: Thank you so much

RES: You are welcome
