# Supplementary material for: “Getting pregnant during COVID-19 was a big risk because getting help from the clinic was not easy”: COVID-19 experiences of women and healthcare providers in Harare, Zimbabwe
Source: PLOS Glob Public Health. 2024 Jan 8;4(1):e0002317. doi: 10.1371/journal.pgph.0002317 (PMC10773929; doi:10.1371/journal.pgph.0002317)
Supplement: S1 Data — (ZIP) [file pgph.0002317.s003.zip › Data/Nurses/Healthcare Worker 2.docx]

**Interviewee’s Gender: Female**

**Interviewee’s Age: 31+ years**

**Interviewee’s Initials: HCW 2**

**Length of Interview: 44:25**

HM: You can start by telling me your name, whether are you married, your age, your qualifications everything can you tell me a bit about yourself?

RES: I am XXX years old married, and I am an RGN Midwife with about 10 years of working experience

HM: Speak up I want it to…

RES: About 10 years of working experience

HM: Have you always been working here or started from somewhere then you later came here?

RES: I started somewhere I started working in Kwekwe then Parirenyatwa then All Souls Mission then Baines Avenue then I came here

HM: Can you please tell me how you feel personally about the COVID-19 coronavirus?

RES: Hmm that disease is something else it’s a deadly disease, that needs people to be extra cautious about it and be serious when we say everyone must sanitize and stay at home you do likewise.

HM: Hmm

RES: Otherwise, if you don’t follow you will end up in trouble

HM: Personally, did it affect you or your family or anyone close to you, how do you feel about it personally?

RES: I was affected by it I had such a terrible experience

HM: Hmm okay

RES: When the COVID started I was very afraid of getting the infection. I worried about getting the infection and passing it on to my mum who is asthmatic. I would dread coming to work and my fear worsened when my colleagues tested positive. As part of the mitigation measures, we resorted to using open spaces for our consultation since our rooms are very small and have poor ventilation.

HM: Hmm

RES: But it got me later

HM: You got infected with it?

RES: Yes, I got infected with it so……….

HM: You are a survivor?

RES: I am a survivor yes

HM: Okay

RES: So, you know you need to be isolated, stay indoors you know it’s difficult and it’s something else

HM: It’s something else

RES: Yes

HM: Okay how has COVID-19 19/coronavirus affected your mental health and general wellbeing if at all?

RES: Okay due to the support of my family members it didn’t affect me psychologically because they were there to support me and to counsel me being a nurse also helped me, I didn’t panic when I was told that I had COVID-19.

HM: Hmm

RES: Actually, I told myself that it’s better to know that you are positive so that you will be extra cautious and prevent yourself and prevent to infecting others.

HM: Hmmm

RES: So, it was of course difficult at first, but it didn’t affect me much

HM: Okay so you had the general support from your family and your relatives?

RES: Yes

HM: What about stress, anxiety or depression did you have any of these 3?

RES: Haa no I didn’t

HM: Can you please tell me about your portfolio of work

RES: Okay as in you mean

HM: Your work generally

RES: Generally how we…routine or

HM: Yeah you can take me trough

RES: Okay as a midwife when you come for duty we report in the morning if you have got a morning shift right you report in the morning and knock off around 6/7

HM: Hmmm

RES: Eh so during the lockdown, we used to meet some patients but they were not many as now as the rules of the lockdown were relaxed

HM: You had a certain number of people that you were seeing on a daily basis?

RES: Yes

HM: How many?

RES: We would maybe book at least a minimum of 10 people

HM: Okay

RES: To try and reduce congestion

HM: Okay

RES: Then when you are in the labor ward they come as they are in labor, so we had no specific number to say we serve per day, but the problem is the patients don’t wear masks (laughing)

HM: (Laughing)

RES: The care givers and the patients just walk in, especially in the labor ward they just come without masks, and one will tell you I forgot it at home how.

HM: There will be in pain.

RES: It’s in the middle COVID-19 and you are not carrying your mask and you expect me to take care of you without a mask honestly, it’s a temptation

HM: It’s difficult

RES: it’s a temptation

HM: You can say that again

RES: Hmm

HM: Okay let’s move on to the health care context in Zimbabwe. Can you describe the health care context that you work in?

RES: Okay here it’s a primary health care facility where we start from, so you need to be vigilant when you are seeing those people so that you don’t miss anything else be it the COVID-19 screening, the HIV, TB, and everything you need to think of when you are seeing them

HM: Hmm

RES: You don’t just focus on one thing, like being a midwife you concentrate on pregnancy you know you must do everything and make sure that you do the integrated care approach

HM: Hmm

RES: Yes

HM: Okay are there any concerns about the healthcare context that you are working in

RES: Yes, sometimes we used to have the problem of water we go for days or sometimes without water at the facility, sometimes there is a lack of PPE sometimes it will be there but very little for everyone so that’s where the problem is

HM: Hmm

RES: You would say you want to come to work and provide some services but when you come to work there are no masks there is no water no sanitizer it's difficult

HM: Okay don’t have boreholes here?

RES: Borehole we have but sometimes it’s affected by electricity

HM: Okay it’s not working because of electricity do you have power cuts as well?

RES: Yes we have power cuts

HM: Okay so you have water rations council water

RES: Council water ah I have never seen it I don’t know

HM: So you rely on the borehole?

RES: We rely on the borehole water yes

HM: Okay

RES: And sometimes we do have some big containers where we would put the water for use but it’s something else when you are talking of the maternity department

HM: Yeah, because how do you work at maternity without water necessary?

RES: Haa it’s difficult

HM: What measures or changes are you making in response to COVID-19 or coronavirus at a personal level?

RES: Yes

HM: What are you doing?

RES: Like for example social distancing, masking, and limiting the visits you. You just don’t go out; you don’t just visit people at their homes because you don’t know what they think of you.

HM: Hmm

RES: A nurse is a front liner you know they will say she brought COVID-19 for us

HM: Yeah sure

RES: So, we are social distancing just to prevent infecting people or being infected by them

HM: Hmm

RES: Yes, and even the way we are handling things at home we changed it teaches us how important it is to know to social distance

HM: Okay what about at organizational level?

RES: Even at the Organisation

HM: What do you do?

RES: We treat everyone as a suspect that this person might have

HM: Coronavirus?

RES: Yes, so you just must keep your distance from people and wear your mask sanitize as much as you can

HM: Okay

RES: I think and eating well

HM: Okay do you have enough sanitizers for now?

RES: The sanitizers we have you can actually have your own if you want to keep like mine I do have a small bottle which I keep in my………….

HM: Hmm

RES: In my pocket

HM: Okay how do you cope with these measures or changes in your life or at work?

RES: Ahh it’s difficult some are taking it as an excuse you know just say because there is COVID-19 now. People are taking advantage of that sometimes you end up not showing people sympathy because you are saying there is COVID-19 so I’m not going there you know.

HM: Hmm

RES: Just to give an excuse but otherwise it’s high time we learn that life is precious

HM: Yes

RES: And we need to love each other even if we are at a distance just a phone call away we call each other and hear if someone is okay you know

HM: Okay

RES: And the changes in terms of maybe work I think we are being affected may be in terms of transport

HM: Okay

RES: Because transport is now a problem these days due to these lockdown restrictions

HM: Only ZUPCOs (Big Buses) are moving around no Kombis (Public transport minibuses)

RES: ZUPCOs yes so the time that you would want to report to work sometimes you end up being very late

HM: Okay

RES: And sometimes going home you end up going earlier than expected

HM: Than the usual time

RES: Yes, than the usual time because you would want to get transport in time to get home

HM: Okay from your perspective how are healthcare workers perceiving the situation this COVID-19 era or this pandemic era?

RES: Okay at first people were not serious about the pandemic but now a I think they are taking it very serious.

HM: Why is that so?

RES: I’m not sure maybe (laughing)

HM: Maybe the first people just thought that it was just a flue a bug that will just go away.

RES: Yes, that will just go away but in the second phase people are now serious even wearing masks and everything people have changed their behavior

HM: They have now changed.

RES: Yes

HM: Now they are serious.

RES: Yes, they are serious

HM: They have seen the number of dying people going up.

RES: Of the people who are dying yes

HM: Okay how has the COVID-19 19 coronavirus impacted the delivery of PMTCT services?

RES: Yeah, it did at some point because I remember there was this other time when we used to have when we didn’t have the HIV determine test kits you know

HM: Okay

RES: So, some women were going without being tested

HM: Okay new pregnancies?

RES: Yes, there are some people who report at the onset of labor unbooked no result you know

HM: And you don’t know the status?

RES: We don’t know their statuses and we did not have the test kits during this pandemic era you will be told that the kits are going to come we are sourcing. It will take some weeks to have them.

HM: The test kits

RES: The test kits to be available so it really affected the women

HM: So it was a major problem in the during the lockdown

RES: Yes

HM: What about getting the medicines for those who are on ART the mothers who are on ART and

RES: Yes, at some point, the ART services were running smoothly I think but the problem was that NVP would be out of stock you can tell the mother to go and buy and how much it cost at the pharmacy it’s terrible

HM: I heard that the cotrimoxazole was not available.

RES: And even the NVP at some point

HM: The nevirapine

RES: It was not there now it’s available though

HM: So, what would happen to those mothers?

RES: You would tell them to go and buy outside or to check with other clinics

HM: Okay

RES: Yes, but it’s a hustle for the woman now you are carrying a baby you need to look around for the medicine it’s hectic

HM: If you want to get tested you want yourself to get tested.

RES: Yes, you know where to go

HM: Okay what about retention in care for both new and old patients?

RES: The…

HM: Retention in care for both the new mothers and old…. new and old patients

RES: The new and old patients

HM: How were you handling it?

RES: Actually, here we handled them quite well but maybe the problem is the time frame that we have with the woman after delivery it's very short

HM: Okay

RES: Because we don’t keep them for a day or 2 or 3 unless there is a problem or usually, we refer them to central hospitals

HM: Okay

RES: So, we do try and teach them what to do yes but at some point, the time frame is very short some are slow learners they will need time to get things right, so I think that's how they are affected especially the first timers

HM: So, all the pregnant mothers were able to seek the services they needed during the lockdown the antenatal care were they all coming, or they were not coming?

RES: They were facing some challenges maybe a mother is booked but now they could not access the services because of the time that we would come to work and the time that we would knock off

HM: Hmm

RES: We would say that it’s better you go and come back later, or you will come when you are in labor but sometimes maybe you would check her if you those PIH ones if you check the blood pressure and see that she might need to see a doctor.

HM: Hmm

RES: That’s when will maybe….take care of them much more than others who are not having problems

HM: Okay

RES: Yes, but I think………

HM: Everyone got a chance to be examined.

RES: Yeah, they were examined but some were facing challenges that maybe the day she comes for scale they might be told that we are short-staffed maybe come tomorrow or another day

HM: Come back tomorrow.

RES: Maybe she has come from far away she doesn’t have bus fare for her to come back tomorrow that’s when you examine them or wait until they come in coming into the labor ward

HM: Okay she will then just come without registering.

RES: We were registering but a few if we take those few to tell her to come back tomorrow and register some will not come back, they will just come when they are in labor or they will go to informal midwives

HM: Were there midwives who were delivering in the community?

RES: They are there, and we heard that…

HM: Do people seek their services there in this time and age?

RES: There are some who are confident in them and prefer to go there

HM: A person who is not trained and who doesn’t have any know-how, on how to deliver. if they have any complications, how do they solve them?

RES: They are there, and they go

HM: Okay are there some people who seek services from traditional and informal midwives who later come back to the clinic?

RES: Hmm they will say sister I have come back,

HM: What about sample transportation, how were you doing it?

RES: Hmm on that we do have a bike the bike would come here and there but some samples and most of the things are done here like DBS we are doing it here but the FBC for the mother maybe and RPR sometimes we would send to the RDH but sometimes but to get the results……...

HM: Hmm

RES: The women don’t do follow-ups of their results now there are so many results there at the antenatal ward

HM: They don’t come to collect?

RES: Yes, but RPR they make sure that we have it here it’s done there and there so that the patient will go home knowing their results

HM: What’s RPR you talking to someone who does not understand the jargon?

RES: The Rapid test for syphilis

HM: It’s done here, and they result they get them on the very day.

RES: They come out on the day

HM: All right what about the ability for health workers to come to work did you have any challenges that you faced coming to work?

RES: That’s the biggest challenge transport was a problem even up to now we don’t have.

HM: There is no sufficient transport yet?

RES: There is no transport, and they are not offering transport

HM: Okay

RES: It came then it disappeared

HM: What were you doing before, how did it disappear?

RES: We used to have transport…………………

HM: How did it disappear?

RES: Ahh things of the City of Harare we don’t know how and where it has ended

HM: How did it go?

RES: We protested about that issue at some point we stayed at home saying that we didn’t have transport, but we saw that the year would end seated at home because they were not considering our concerns.

HM: Seated at home?

RES: Yes, because we did not have any transport and they were not doing anything

HM: When you were traveling were you traveling well did you have any challenges on police at the roadblocks?

RES: Some of us who stayed close to the clinic did not have problems with the police roadblocks

HM: Okay you were just walking coming to the facility?

RES: We were walking but otherwise some days when you are hard-hearted you would not come to work.

HM: Okay what about on fear of getting infected with COVID-19 19 how were you doing it?

RES: On?

HM: Fear of getting infected with COVID-19 did you fear for yourself when you were coming to work?

RES: Yeah, very true we were afraid, that we will get infected unexpectedly

HM: Did you have enough PPE or was there’s lack of it wasn’t...?

RES: PPE wasn’t enough.

HM: The City of Harare wasn’t providing?

RES: It wasn’t you would find that you could be given 1 mask you spend the whole day with and go home with it.

HM: When you are supposed to spend 3 hours with it then you throw away then you will be given another one?

RES: Even if it gets wet you need to change it. We used one mask and there is no water to wash sometimes sanitizer finishes, how would you work? Or……… there is no liquid soap to wash our hands ahh the things are hard…….

HM: So now is there anything that has changed or it’s still the same?

RES: Yes, things have changed though

HM: How have the things changed?

RES: Slightly because we are told that disposable gowns are finished, and you must wear one gown for the whole day. We have many staff here and they bring a few things.

HM: So, it won’t be enough for everyone?

RES: Hmm it won’t be enough for everyone

HM: How does the COVID-19 situation…. aah compare to other outbreaks if we were to compare it to cholera or to typhoid that we once had how would it?

RES: COVID-19 was too much than cholera and typhoid. I think they were better handled than COVID-19, I don’t know if it’s the issue of money or what made COVID-19 unbearable because for a person to be tested for COVID-19 you needed a lot of money.

HM: How is that?

RES: It was too expensive as if it was not a disease like other diseases

HM: Hmm

RES: I don’t know why it was like that I don’t even understand why they used that approach that the things and supplies will be that expensive. Or else they took on that it’s a disease that is infecting rich people or what I don’t even know but it was expensive to test and all.

HM: But it affected everyone the rich, the poor, and everyone.

RES: I think that's what they thought at first, but they later saw that everyone can be affected by it but hmm it surprised me the way it was done

HM: Okay so it was worse disease was even worse than cholera and typhoid.

RES: Hmm that’s right that’s what I think

HM: And infections as well

RES: Yes, and the issue of fear is that you know if a person knows that if you get infected you are gone. People had a fear that …. even as a healthcare worker if you were told to go and work at the Out-patients Department (OPD) you would say No spare me I don’t want……. (laughing).

HM: I don’t want? (laughing)

RES: Because it was bad.

HM: Okay describe the changes to your service provisions as a response to the COVID-19 pandemic. Were there any changes to your service provision that changed, or it's still the same?

RES: I think what I can say is it changed …. people won’t enter the gate without being screened and we have a limited number we serve per day because we allow them to enter in batches and the large number will be outside the gate.

HM: The gate?

RES: Yes, we don’t want people who will be Walking around in the clinic yard

HM: Hmm

RES: Everywhere at the institution those who would want to be helped will enter in batches. I think that’s how it changed.

HM: All right, what challenges do you think mothers encountered in trying to access PMTCT services during a lockdown?

RES: The challenges?

HM: Yes, that was encountered by mothers in trying to access PMTCT services during the lockdown.

RES: As I was saying at some point, they couldn’t find test kits then there was no NVP I think it affected them a lot

HM: All right

RES: Knowing that it’s their right to get those things and they say have I booked I am supposed to get those things done and you will be told that we not doing that today and to come back some other time, there are no test kits we will test you after giving birth. Also, to be told that there is no medication go and buy.

HM: Hmm

RES: And the people you are dealing with are very poor some of them don’t even have the money, you will see that yes you are saying go and buy but

HM: Hmm

RES: You will see that they cannot afford it and it is dangerous, so I think it was hard for them

HM: What was happening and why was there no stock of Cotri and NVP what was happening?

RES: I don’t know maybe they were affected by their ordering or there……. I don’t know. I work here at maternity I don’t know what was happening at the pharmacy.

HM: What was happening did you not anticipate that those people would come for those services and the consequences it would have on children?

RES: Eh the issue that is there is a follow-up that must be done, and we say you don’t get discharged if you have not gotten your medication

HM: Hmm

RES: But there is a point I remember on the weekends even if we say you don’t have to go home without medication, and she cannot go to other satellite clinics or Tafara because they will be closed on weekends. At the same time, we cannot keep her at the facility because we do not offer food and that patient will suffer.

HM: What will she do?

RES: At the end of the day, you will end up saying go make sure that you come back to get your medication or if you can afford you will have to buy the medication for the baby.

HM: Hmm

RES: If they come back, you will give them then they will come back for testing after 6 months

HM: Okay

RES: After 6 weeks sorry

HM: So, do they follow, or do they just say it’s for nurses it’s not a big deal?

RES: I think there was a focal person who was dealing with PMTCT who was following up on the cases I’m not sure if he/she is still there

HM: Following up mothers?

RES: Yes, following up the people

HM: Okay do you think your patients had all the information they required during the lockdown, they knew where to go for PMTCT services during lockdown did they have the information?

RES: When we book them, we do give them information but it’s up to her whether she wants to take the information or she doesn’t want usually that’s the first thing that we tell them when we are starting to book them.

HM: Hmm

RES: We are going to test you if you are positive, we will give you some medication then your baby needs to be given medication like that, then also your partner we would want to test him and even other children at home

HM: Hmmm

RES: So, you wouldn’t know if a person follows

HM: What will they do?

RES: Yes, but otherwise we do try our level best so that we will not miss opportunities

HM: Did they know how to get to the clinic including the travel requirements that when they are going to the clinic, they are supposed to have this and that?

RES: We do provide that kind of information

HM: Hmm

RES: Yes

HM: How to handle themselves at the facility when they come here

RES: Everything we tell them

HM: Hmmm have you noticed any changes in the number of patients seeking care since the onset of COVID-19?

RES: Yeah, the changes are there

HM: Has it decreased, or has it increased what did it do?

RES: Number?

HM: Yeah

RES: The number I think for it to decrease they would have been told that Mabvuku poly is closed but otherwise the numbers keep going up.

HM: Okay

RES: Because that’s the major facility in this area

HM: Hmm

RES: Yes, so they would want to be seen doing the work

HM: All right social issues at home in your opinion how do you think the following factors affected women’s access and utilization of PMTCT services, HIV disclosure what was happening to women on disclosure of HIV status during this lockdown?

RES: Haa it was bad

HM: Why are you saying so?

RES: The challenge with some of these women is that they stay with their in-laws and other relatives who are not aware of their HIV status so it becomes difficult for these women to freely access services unlike before the pandemic when they could access services without telling anyone at home that they were going to the clinic. Now that everyone knows that movement is restricted, they would question their whereabouts.

HM: Hmm

RES: They spend the whole day selling but where can they sell to get money

HM: They are in lockdown….?

RES: There was no more moving around, so it was very difficult

HM: Okay

HM: How about the disclosure of their statuses?

RES: Yes, we always encourage them that you need to disclose but it’s difficult

HM: Okay but don’t you encourage the pregnant mothers to come with their partners when they are booking?

RES: Some come with their partners but some just come, you know that sometimes we meet some people who are difficult. I remember there was this other person who knew her status she was tested, and she knew, then she just went or the day that she was tested she just disappeared she never came back, and nothing was done to her.

HM: Hmmm

RES: Then she came back pregnant they saw that the person was positive and the counsellors said she was in the tablet system we tested her on this and that day, and then we asked why it is you are pretending as if she is someone new.

HM: Hmm

RES: The stories that you will start to hear there you will see that even disclosure will not come out

HM: Did she come alone, or did she come with her partner?

RES: She came alone, and they come alone it’s not everyone who brings a partner some they do but some they don’t

HM: Are you still encouraging them that please come with your partner?

RES: For those who come with a partner we make them go home quickly we serve them faster so that they can go

HM: All right

RES: So, on that, some don’t care about bringing a partner

HM: So that they can get tested together so that they know our statuses.

RES: Ahh at home there is another one that I saw she came with her boyfriend they were students from University I don’t know which university

HM: Hmm

RES: The girl got pregnant they have been tested the girl is positive the boy is negative. They went their separate ways when they left the facility it was bad.

HM: They couldn’t agree/

RES: They’re no longer agreeing

HM: The other one didn’t accept the status?

RES: Hmm it’s difficult

HM: What about childcare roles and responsibilities during COVID-19 is there anything that was affected there, what was happening to taking care of children and the parents responsibilities and their roles

RES: To children in the community?

HM: Hmm

RES: I think the children had the biggest jobs at home since they were in lockdown and not going to school, they were the ones who were spending the whole day doing housework

HM: At home where will be the mother?

RES: She will be seated whilst the children will be working for her

HM: Hmm

RES: You would see many who didn’t have money to do online lessons playing on the roads

HM: Okay

RES: Otherwise, those with money could afford to keep their children busy with schoolwork but some couldn’t

HM: What about….?

RES: And the cases of child abuse I noted that they increased especially on the first lockdown.

HM: Hmm what was happening?

RES: The rape cases of children and children being mischievous

HM: On their own?

RES: On their own

HM: Is it about supervision?

RES: It’s difficult

HM: So how did you handle those cases?

RES: There are people who were trained about the …… what is it gender-based violence and referring them to the police maybe for rehab there is somewhere they are taken I don’t know

HM: At least if cases like that were not swept under the carpet the better.

RES: I think about 2 or 3 cases that I noted

HM: Was it being caused by the lockdown or that the children no longer have anything to do they have ample time to do anything.

RES: Those who had left their family houses to rent their own houses and stay alone had to come back to the family houses because they could not afford rent and they were crowded in the small places

HM: They were staying in one place?

RES: Yes, one place

HM: Then decision-making powers both at the household and community level what was happening.

RES: Decision-making powers?

HM: Yes, was it still the same or did it change did the powers shifted or what happened at home and at the community level during the lockdown?

RES: I think that’s when we are talking about gender-based violence increased because the issues were problematic in homes.

HM: Hmm

RES: Eh maybe the mother is used to doing her things now that she is being supervised because the father is home and wants to be involved in how the kitchen is being run by the mother to see what’s going on, so it really affected some families at some point.

HM: Okay what about access and control of resources what was happening who was controlling resources at home?

RES: Will they be there…………….

HM: That is when one goes from hustling his or her dollar someone will say you had gone out so give me the money.

RES: Haa those things are difficult it all depends on the family setup, I think. Otherwise, to some its difficult but to others it makes them bonded and make their relationship stronger.

HM: Okay

RES: Some even separated because couldn’t cope (laughing)

HM: With the situation

RES: Yes

HM: The Zimbabwean government has implemented major social changes including social isolation travel restrictions closure of schools and borders what impact do you think they might have on women in your catchment area.

RES: It really affected most women a lot because some were cross-borders traders they would go there to buy their things then come and resell so that they could pay fees, rentals, and buy food for their children so it was hard

HM: They were affected greatly.

RES: They were affected

HM: Okay do you think these measures are feasible especially in your catchment area that of travel isolations social isolations travel restrictions, closure of schools and borders was it feasible?

RES: Yes, it was but a person will find a way for him/her to survive after seeing that this avenue has been closed, I need to open another you no longer stand with one thing, just because the borders imagine how many years now………..

HM: Hmm

RES: It’s now 2 years the borders are still closed so it’s helping even though they can do online what.

HM: Businesses.

RES: Some have started they’re doing it.

HM: What measures or programs are needed to mitigate the negative impacts of COVID-19 within your community?

RES: I think the provision of food

HM: Hmm

RES: There are organizations who are doing it like the World Food Programme who are assisting families who are vulnerable I think it’s a good gesture in our community

HM: Hmm

RES: And I even hear some saying that the school fees were being paid up

HM: Okay with those NGOS

RES: With those NGOs of which I think it’s supposed to be done; I think if the government schools could say children must just learn not paying fees

HM: For this period?

RES: For this era of the pandemic if they could say children can learn for free.

HM: Hmm

RES: For free then they will see in the future how it is because some they are not even capable for them to be able to find be it 15 or 25 dollars for fees.

HM: For fees

RES: It’s hard not everyone will be sponsored by the donor so it’s as hard as it is they should consider that for children to learn.

HM: Alright

RES: Yes

HM: Okay what do you think are some of the health impacts of COVID-19 including beyond the infection itself that even after it has passed beyond what are the health impacts of COVID-19 that will remain.

RES: COVID-19 we have them some families lost parents were left they are now orphans.

HM: Hmm

RES: They lost parents and to start afresh it’s hard even the way we are living now and the issue of distancing it affects some people, that aunt that would come to visit and see you. It’s no longer possible you see.

HM: Hmm

RES: Because of COVID-19 some were even affected by conditions like depression you see it's now a…

HM: It will remain there after the pandemic…….

RES: It’s other conditions that you are left with of which is bad.

HM: Okay all right, what do you think are the socio-economic impacts of COVID-19 in the short term and the long term?

RES: The socio-economic

HM: Hmm impact of COVID-19?

RES: I usually look at the SDGs here where we are saying by 2030.

HM: By 2030 or 2022?

RES: It was said 2020 now we are focusing on 2030.

HM: Yes 2030

RES: But the one that says no poverty I think poverty is now there and it’s too much, it had not decreased but it has increased.

HM: We have taken several steps back.

RES: Yes, we have drawled back I don’t think we are going forward here.

HM: Hmm

RES: Then the issue of food and security I’m not sure about the rains but not everyone was able to buy fertilizer and so forth.

HM: Hmm

RES: So, it’s a bit difficult with food and security and the issue of health that some even can’t even access health facilities they can’t afford to pay for them to get the treatment they need.

HM: Hmm

RES: Some will die in their homes and I saw it during the time of COVID-19 even a person who is not sick with a disease that could kill him/her because people had lost confidence in health facilities, a person would reach a point of dying people saying there is nowhere they are accepting people they are saying COVID-19 test in front and the money for COVID-19 test you don’t have.

HM: You don’t have.

RES: You know people would just die then you see after being tested after he/she dead and see that he/she didn’t have COVID-19 but you have left a person at home because you cannot afford a test.

HM: Hmm.

RES: It’s some of the things that are difficult

HM: All right what do you recommend should be done as a national response to COVID-19, what do you think can be done as a response national response to COVID-19?

RES: National response to COVID-19 19

HM: Yes

RES: As I have said they must keep on assessing to see which people are vulnerable and assist them with long-term projects and their life can go on.

HM: Hmm

RES: Yes, fine you gave me food today, but it will finish tomorrow then what am I going to do.

HM: Hmm

RES: They must maybe give people loans and make hospital fares affordable to everyone I think it might help cause people are going to die of disease at homes without going to the hospital to get treatment.

HM: Okay what measures or programs need to be put in place to help mitigate the negative impact of COVID-19 at the workplace and within the community?

RES: At the workplace, I think the issue of good working conditions and remuneration. I think if they can be good, people will be motivated enough that even those who are seated at home who are saying I am not going until COVID-19 has ended they will come and join us.

HM: Hmm

RES: So that we can work together unlike how it is now those with money will be saying I am not going anywhere, I am putting aside my certificate and staying at home to be safe so I think if they do that that will be good.

HM: Hmm

RES: Then in the community, if they give people money for projects, they can start to do something that will give them money tomorrow. I think it will help even at school If they can make children especially primary level learn for free, I think it will help.

HM: Hmm

RES: Those who want to send their children to expensive schools and private I think that will be fine but as we speak right now some children are seated at home they are not going to school because parents cannot afford it.

HM: Okay thank you, sister, those are all the questions we had for you about what was happening during the time of COVID-19, how were people handling the cases they were encountering the challenges they were encountering, you have given us an insight of what was happening that next time if another problem like this happens in the future we are better prepared these unlike now we were caught unaware that the health system doesn’t know what they can do where can they start from on that problem that has come to us so this will help to create a communication package that in case we encounter another problem like this they will know how to handle the situation better.

RES: Okay

HM: Thanks very much

RES: You are welcome

HM: Go inside where Concy is and talk to her there she will give you the reimbursement that you are supposed to be given

RES: Okay thank you
