# Supplementary material for: “Getting pregnant during COVID-19 was a big risk because getting help from the clinic was not easy”: COVID-19 experiences of women and healthcare providers in Harare, Zimbabwe
Source: PLOS Glob Public Health. 2024 Jan 8;4(1):e0002317. doi: 10.1371/journal.pgph.0002317 (PMC10773929; doi:10.1371/journal.pgph.0002317)
Supplement: S1 Data — (ZIP) [file pgph.0002317.s003.zip › Data/Nurses/Healthcare Worker 4.docx]

**Interviewee’s Gender: Female**

**Interviewee’s Initials: HCW 4**

**Interviewee’s Age: Around 45 years**

**Length of Interview: 29:54**

ZM: First, I am going to ask a brief background about yourself how long you have been here, and your role at the Mabvuku clinic.

RES: I guess I can mix language

ZM: You can feel free to mix Shona, and English if it’s okay,

RES: I have been here since 2014 as a sister in charge and basically more on the maternity side though I also overlap with the outpatient department.

ZM: Okay and maybe your portfolio of work, what it includes like the current assignment the duties what does your portfolio of work include?

RES: Okay basically it has to do with planning, organizing, directing control and monitoring and evaluation planning of services basically looking at the City Health goals, and we get our objectives from those.

ZM: Hmm

RES: Then we also plan in terms of human resources in terms of staffing delegation we do what and when in terms of programs that come in like from the ministry currently, I am focusing more on the elimination of mother-to-child transmission of HIV/AIDS its more on the maternity side

ZM: Hmm

RES: Then other programs which come in with other we will be doing research basically support services then organizing of resources maybe availing SOPs at the facility and maybe on job training orientation of staff, staff motivation, appraisal reports.

ZM: Hmm

RES: Supervision basically disseminating information, checking work performance, control performance checking on efficient and effective use of resources, and identifying knowledge gaps, trying to address those maybe acting as a strategic person for upward and downward information exchange.

ZM: Hmm

RES: And maybe also problem-solving decisions and also just maybe general maintenance of the facility although we work in line with the district administrator half the time they are not here.

ZM: Hmm

RES: And integrating services we work with the community we have a community linkage system with partners in terms of the provision of quality services and together with the other auxiliary departments.

ZM: So, because of this issue of COVID that came so how do you feel personally about COVID-19 does it frighten you, what how do you personally feel about the coronavirus?

RES: I think at first it was quite frightening because we were just hearing stories then when it really came into Zimbabwe people were dying and I even lost a close relative its actually yeah, it’s frightening.

ZM: Hmm

RES: And the death process it was so sudden so ahh……. frightening disease but maybe because we are now used with just working with clients and we have no other choice that’s the type of work we are doing.

ZM: Hmm

RES: We are used but at the same time it’s a bit frightening because we don’t have like adequate PPE, and we had 12 of our staff members who got infected though it was more of mild cases.

ZM: Okay and how has COVID-19 affected you personally in terms of your mental health and general wellbeing have you been at one point anxious, depressed, or stressed how was your mental health and well-being with regards to the coronavirus?

RES: Yeah, basically a bit of burnout because being the sister in charge especially the time when other staff members got positive, they really needed counseling, so it was just me being also a swab head tester. I was doing the testers with the lab staff, and the positive results the staff members needed counseling because some of them did not take it lightly.

ZM: Hmm

RES: They were really devastated so I was rather overwhelmed we had a staff shortage because most of them had to go off and I had to fight for the clinic to be closed so that at least we would give that space so that we wouldn’t infect the community, so it was rather strenuous.

ZM: Hmm

RES: Because even when you are off you still must continue communicating with them but from the phone calls you could hear that hmm, they were not okay it was a bit disheartening

ZM: Alright then just looking at the health care context in Zimbabwe can you describe it to me maybe comparing it to what was there in the 1980s like the terrain now when you look health care context in Zimbabwe now? What can you say about it?

RES: I think maybe I would say soon after independence they tried to implement the primary health care concept, then came the ESAP things went down there were problems with the health delivery system

ZM: Hmmm

RES: Then decentralization fine it was okay for the patients but in terms of quality, care looking at the economy of the country things are bad because there’s a lot of brain drain a lot of experienced personnel had left the country for greener pastures

ZM: Hmm

RES: And in terms of infrastructure for now things have just been static there is nothing moving because half the time we are just depending on donations from donors we are not independent as a nation

ZM: Hmm

RES: So basically, in terms of staff motivation in terms of staff retention, in terms of infrastructure developments things are getting down

ZM: Okay then coming back to the COVID era what sort of measures did you put in place at a personal level starting with you as an individual to try and sort of prevent or reduce the risk of transmission as a nurse as a health care worker?

RES: Yes, at the facility we begin at the facility because that’s where we would spend most of our time.

ZM: Hmm

RES: It was thorough vigorous screening at the entrance for all clients and we had a questionnaire for those basic signs the fever, the loss of test loss of smell, the headaches so we would really screen at the main entrance

ZM: Hmm

RES: And that helped to decongest in the facility and those with symptoms or with fever, we had our own designated area where we would then test because we are testing at the facility doing COVID antigen test.

ZM: Okay

RES: If the person was positive, we would then communicate with our national RRTT, or they would use their own transport to Wilkins Hospital.

ZM: Hmm

RES: So that vigorous screening limited transmission as soon as we got a patient who was infected, we tested then all those clients who would get in we had a register at the entrance they will be taken as contacts so we would also follow them up.

ZM: Hmm

RES: And besides that, screening, we would have a basin for and washing at the main entrance and the sanitizing clients and giving health information like at intervals because clients they just come and…. they didn’t know what was happening.

ZM: Hmm

RES: So we would continue emphasizing even the wearing of the cloth mask and even how to manage the ask at home washing it and also we would really emphasize on home care even for the patients issues to do with ventilation, personal hygiene the cough etiquette and we would emphasize even when the patents were inside we work as a team with the municipal police

ZM: Hmm

RES: Because they will be outside most of the time so that they could maintain that 1 meter distance the physical distancing and even consultation rooms we should make sure that its 1 patient at a time

ZM: Hmm

RES: Although this had a negative effect on the quantity in terms of numbers we would trim in terms of numbers because it was screening at the gate, a lot of procedures then trying to decongest the area

ZM: Hmmm

RES: We would also limit even in terms of those clients who are coming book for ANC because our space is a bit limited so for us to be able to maintain the one meter distance we would maybe book about 10 a day

ZM: Hmm

RES: Otherwise without COVIDwe would go even up to 30

ZM: Okay looking at these measures do you think you are managing to cope with the number with everything else bearing in mind these measures that we have just talked about

RES: Yeah I think we are coping initially when we tried to implement this it was quite a challenge you know change is a process but when most of the staff members got positive that when they release that this thing is real because you know people believe in seeing

ZM: Yeah they need to see first

RES: So even the patients because we are in a community you know the message travels fast and they were saying all the nurses at Mabvuku are dead because of (laughing) because of COVID-19, so you know that fear it was just an awareness for readiness for change

ZM: Hmm

RES: And everybody was now geared for the change and you would see everybody wearing properly the mask, trying to screen even the distancing yeah that when people became serious. Even the patients because patients now were even afraid of the nurses

ZM: Yeah okay

RES: I think it worked although in a negative way but it really worked but now I think people are used

ZM: They are relaxing again

RES: Hmm

ZM: Then looking at the uptake of PMTCT services do you think the COVIDhad an impact on the delivery of PMTCT services

RES: Yes it had a lot because issues to do with transportation because most of our clients they come from Caledonia and most of them they don’t even have cars at home they cannot afford even to call an ambulance from home

ZM: Hmm

RES: So you find they could end up delivering either at home or on the way but we had a 2 weeks of great interruption of services that was in January I think from 8^th^ to the 17^th^ of January we were closed

ZM: Hmm

RES: So it was terrible because from that time that’s when we discovered there are so many midwives in the area

ZM: Hmm

RES: Doing deliveries and they ended up having patients coming in with sepsis, some of them even not even coming for HIV testing because those 2 weeks it actually disturbed a lot

ZM: Hmm

RES: Even when we opened the transportation system was you know was just poor so half the time they would not come and it was actually interrupted even with the messages maybe they did not get the correct message in the community

ZM: Hmm

RES: Some thought we were still closed they were not sure even if you look at our statistics in January they were very low then they started picking in February but from March we can say services are almost to normal but we had quite a big interruption

ZM: What about in terms of the testing commodities and the supply of medication were there any interruption like in how you normally receive your commodities and also how you normally sent your requests In terms of getting new supplies were they any interruptions?

RES: Not as such because even when things were okay we just used the visual electronic to send orders, the orders were coming and fortunately enough we stock about 7 monthly supply that’s our minimum our average months consumption so we had stocks because patients were not coming in as many as we expected so we never ran out of stocks

ZM: Okay what about in terms of sample transportation for those that were coming for viral load, for those that were coming for HIV testing were there any interruptions in the actual transportation of samples to the lab and also in terms of getting the results

RES: Okay basically our advantage is we have a lab on this site supported by CHAI for the nucleic acid testing, at birth testing so we are doing it here even the viral load for pregnant women that’s good for us

ZM: Hmm

RES: But the only challenge was coming in terms of the FBCs and the other routine test for pregnant the otherwise in terms of HIV testing is here, the viral load is here, the at birth testing, the nucleic acid testing it’s all here and you would get the result on the same day

ZM: Ah okay

RES: So there was no interruption in as far as that was concerned

ZM: What about the ability of healthcare workers to come to work were they any challenges that they encountered during the national lockdown like the first one that was intense?

RES: Yeah they were challenges with transport it was like instead of reporting at you know the usual 8 you report after 3hrs

ZM: Hmmm

RES: Transport was a nightmare because there was so much pressure at the ZUPCO buses. You wait at the bus stop for 3 hours waiting for ZUPCO and you will get to work at 9 am and even going back home, it was the same so because of the shortage of transport so we were now working till 2 pm so that we will have time to look for transport and get home before curfew time

ZM: Hmm

RES: So I tried to devise my own duties that were not formalized I will just communicate with the DMO that this is what I am doing but it weekend, it’s like that the duty would go like those who come in the morning for that day they come and they work up to 1

ZM: Hmm

RES: Then others will come at 12 we would call it a call shift at 12 then from 12 o’clock until the next morning so this gives space for them and from 12 o’clock to 1 o’clock that’s the handover takeover

ZM: Hmm

RES: Those who had come in the morning will go off so it was like actually motivating them because those coming in the morning they would wake up very early to come here on time like say 8 o’clock will be on duty, so that is what is happening so there is no problems

ZM: Okay

RES: So they would go earlier so that they will get transport earlier to go home

ZM: Okay and in terms of PPE supplies of PPE did it have an impact in actual in terms of service delivery that maybe at one point you didn’t have enough, do you think availability of PPE affected the delivery at any given time?

RES: Yeah PPE up to now it’s still a challenge we never had adequate PPE but the advantage which came with unavailability of PPE was that people got used to COVID-19 and ended up understanding really what to wear instead of informs of risk assessment

ZM: Hmmm

RES: Initially there was no risk assessment you would just put everything because people were afraid but when we now got used to it and we tried to talk about it and you know listen to researches and whatever then that’s when people they knew now that they have to be a risk assessment

ZM: Hmm

RES: There was no need of putting just everything the Tyvek suits, the goggles the face shields for any other procedure so at least it was better but though in general PPE is just inadequate because maybe half the time they would spend maybe the whole day with one mask

ZM: Hmm

RES: So especially in labor wards but now it’s actually better the other departments were they do not need much of the PPE they now know how to do risk assessment but otherwise everybody from the municipal police would wanted a Tyvek suit it was overwhelming but now at least people are getting used

ZM: Then looking at like your experiences with previous out breaks in Zimbabwe we had cholera outbreak, we had a typhoid outbreak how does coronavirus compare or how is the situation or how does it compare to your experience of handling other out breakbeats in the past when you look at coronavirus how does it compare with cholera and typhoid?

RES: I think with coronavirus the rate was faster because I think in terms of containing the transmission I think as a country I would say we did quite well

ZM: Hmm

RES: Because maybe comparing to other outbreaks the response was not as abrupt as it was with COVID-19, I think maybe because the bigger powers were involved

ZM: Hmm

RES: So when they have declared the state of emergency I think maybe people are afraid of the police or higher powers but in terms of you know the cholera it was just a certain area, so if someone was not even exposed to that maybe didn’t not even have a relative who died of cholera or was maybe living in Budiriro we thought maybe it was just something it was a story

ZM: Hmm

RES: But with COVID-19 it involved everyone and now that maybe everybody has got WhatsApp phone hearing those mystery stories from China and everything and even on the news you would open CNN and it’s about talking these deaths which every news station was talking about people dying

ZM: Hmmm

RES: So I think people were just geared for prevention and I think nobody wants to die but with cholera it was not like as

ZM: Okay then in terms of challenges do you think mothers who wanted to access PMTCT services encountered any challenges in trying to access the services during the national lockdown?

RES: Yeah

ZM: What sought of challenges do you think they encountered?

RES: I think so because it’s unfortunately that some of them we didn’t not even meet them because the first challenge was transportation to come to the health facility because of the road blocks and the letters you know they would be asked a lot of questions

ZM: Hmmm

RES: And also issues to do with even money because most of them they are in the informal sector so really coming to the facility to them if you are not sick it was a luxury

ZM: Okay any other challenges?

RES: Ahh basically those 2 mainly

ZM: Okay do you think they had all the information they required during the national lockdown like the mothers do they know where to go for PMTCT services did they know whether the services were available what sought of information did they have during that time?

RES: Yeah I think there was confusion because they didn’t know which facilities where open because with the Eastern district this side the only facility which offers 24 hours maternity services is Mabvuku Poly

ZM: Hmm

RES: So because of you know the information that was going on in the communities the clinic is closed, all the nurses are dead some are sick, so it took time for them now to get confidence that the clinic is really working so it was more of the WhatsApp messages you know

ZM: Hmm

RES: There was no contact interaction so it was difficult because even with the community health workers it was just working using WhatsApp groups so it was not quite effective so they did not have enough information

ZM: Okay and in terms of how to handle themselves like the few that were able to access the services did they have the information to say when I get to the clinic how do I handle myself so that I reduce my risk of getting infected and also reduce the risk of infecting others with COVID-19 do you think they had all the information during the national lockdown

RES: They didn’t have even up to now they didn’t have so it’s like as clients come in we just we keep on giving information and reminding them even especially on the physical distancing they would just come even 2 benches they would squash on one bench the other bench will be empty

ZM: Hmm

RES: So you have to remind them can you maintain the distance proper wearing of masks so you know it’s something they think maybe it happens when they are at a facility or I don’t know because it’s not within them you have to keep on reminding them

ZM: Okay in terms of social issues at home do you think there were issues with regards to HIV disclosure now that everyone is at home the father is at home the mother is at home they are not traveling they are not going outside they are home. Do you think there were issues in terms of HIV status disclosure between couples during the national lockdown?

RES: There was a lot of actually domestic violence maybe because they were not used with staying together

ZM: Hmm

RES: Yeah they were issues because you could find that some of the women would not even come here and those who were coming here you know they would prefer like keeping their medications here

ZM: Hmm

RES: Issues of disclosure because say my husband is always there I normally hide then under my mattress so I cannot access them, is it okay maybe the effects we will see them maybe alter because you know with pregnancy they look well nothing is happening, we could only discover that viral load is high

ZM: hmm

RES: The issue of disclosure is a big issue we have so many cases of women who refused to disclose their status to their partners. So being in the same environment all the time, it was quite difficult, some were not taking it well while others could not observe time but would randomly take any opportunity that disrupted their routines. It was difficult and I think we will see the effects of COVID-19 on adherence soon enough.

ZM: Hmm

RES: So being in a same environment all the time it was quote difficult some were saying sister can I leave them in your office so that I can come and get, but you find that ah trying to get other strategy they say I cannot move it and give to my friends its difficult so I think we will see the effects in the near future I think

ZM: What about in terms of access to resources do you think they had access to resources during the national lockdown?

RES: They didn’t have because who were coming either they were labor or maybe those who were well informed that will not be asked at the roadblock but most of them you know with people they just have fear of the unknown

ZM: Hmm

RES: They would say we are not supposed to be moving I don’t have a letter so I will just stay at home

ZM: Then looking at the different measures that were put in place to sought of contain the spread of the virus the issues of social isolation travel restrictions the closure of schools, do you think they are feasible in the community that you service looking at Caledonia looking at Tafara, do you think these feasible strategies if you look at isolation itself is it a feasible thing looking at the setup in Caledonia, Mabvuku and Tafara

RES: They are not because somehow I think God has mercy on people because otherwise the way business is done at Caledonia you will collapse

ZM: Yeah

RES: They were not wearing masks it was business as usual maybe once in a while when the police would come but otherwise it was business as usual

ZM: hmm

RES: Otherwise physical distance ah there was nothing like that, even most of them maybe they can open one room and they could be crowded even the ventilation ha yeah it was just Gods mercy that they didn’t get otherwise Hmm the measures were not feasible and are not even feasible these communities

ZM: Okay what do you recommend to be done like in future to ensure that there is no service interruption, like if we are to have another outbreak maybe 2 years down the line or whatever, what sought of measures can be put in place by the clinics by the government to ensure that there will be no interruption of services in case we got another humanitarian emergency or another humanitarian crisis. What can be done now so that in future we don’t panic we don’t see this huge interruption?

RES: It depends now with the type of pandemic if it’s something that we now know like COVID-19, we now know what it is the IPC measures to be put in place but if it’s something really new, it’s quite difficult

ZM: Hmm

RES: Yeah it’s quite difficult but when it’s something that like COVIDwe really need to make an emergency preparedness response plan to know what do we do when these things happens

ZM: Hmm

RES: Where are the patients going and we also need to have the system decentralized that’s the most important thing

ZM: Hmm

RES: So that we have like a rapid response team at the ,you know local level because what was happening people would phone for the rapid response team they would not come

ZM: Hmm

RES: And they end up coming here sister what do we do they are not coming my father needs oxygen so at least decentralizing those facilities maybe say per if possible per district would know we are taking patients there it would quite help

ZM: Hmmm

RES: And also educating people with all forms of you know IEC material maybe through the radio even in schools so that people are always aware of what to have and what to do but otherwise this one people didn’t not have information

ZM: Okay

RES: They were just working on assumptions on what was circulating on WhatsApp groups and sometimes it was not true

ZM: Thank you so much those were all the questions that I had for you
