# Supplementary material for: “Getting pregnant during COVID-19 was a big risk because getting help from the clinic was not easy”: COVID-19 experiences of women and healthcare providers in Harare, Zimbabwe
Source: PLOS Glob Public Health. 2024 Jan 8;4(1):e0002317. doi: 10.1371/journal.pgph.0002317 (PMC10773929; doi:10.1371/journal.pgph.0002317)
Supplement: S1 Data — (ZIP) [file pgph.0002317.s003.zip › Data/Nurses/Healthcare Worker 6.docx]

**Interviewee’s Gender: Female**

**Interviewee’s Age: Around 50 years**

**Interviewee’s Initials: Healthcare worker 6**

**Length of Interview: 59:54**

CM: We are now starting our interview like what we talked right, can you tell me a bit about yourself, about your age, your marital status, your qualifications, and your designation at this clinic?

RES: My name is XXX, I’m around 50 years old, and I’m married. I started training as an SCN from 1990-91 then I…

CM: As a what?

RES: SCN

CM: SCN what is that?

RES: State Certified Nurse

CM: Okay

RES: Then I upgraded to an RGN from 1996 -1997

CM: Hmm

RES: Then from 2004 to 2005 I trained as a midwife. In 2018 I graduated with a BSc in Nursing where I studied with ZOU.

CM: Okay

RES: I am working here as a registered midwife nurse.

CM: Okay hmm

RES: Mostly, I deal with maternal care

CM: Women, infants, and those with children

RES: Yes

CM: Can you tell me how you feel personally about COVID-19 or coronavirus?

RES: If I’m looking at the thing called COVID-19/coronavirus I feel touched.

CM: Hmm

RES: Because it’s a thing that came unexpectedly and when it can we have relatives who passed away for example in the area where I stay, we lost several people.

CM: Hmm

RES: Even if we look at us here there was a time when this clinic was closed for 2 weeks after we had many positive cases and even some who were negative were ill, but we want to thank God we didn’t lose anyone

CM: Hmm okay

RES: Another thing we want to that God is that we didn’t lose any relative here, but it’s a painful thing, because if you think if you are sick coughing and what, for me, there was one time that I would spend the whole day sleeping I couldn’t wake up and I was feeling that I am very sick.

CM: Hmm

RES: Then I reached a time when I said that maybe I wasn’t tested well, why didn’t I come out positive because I was sick?

CM: Okay did COVID-19 affect your mental health and your general well-being you have talked about being sick but mentally how did it affect you?

RES: There was one time I was depressed when we were tested, I went home when I arrived told my family, and that time my family accepted me.

CM: Hmm

RES: But there was one relative of mine that you just said in a group.

CM: Family group?

RES: Yeah, then I said Aunt I’m not feeling well, and I told her that I have COVID-19 and she said ah we can’t come to your house, we are no longer able to see you, but what I’m grateful for that time was my family I would wake up and bath then I sit in my bedroom then I open the window.

CM: Hmm

RES: I had told them, and they understood but I have a grandchild who’s in form 3 the other day he what if you die in there he entered and sat with me.

CM: Hmm

RES: He said you always sleeping, and you are not eating because we have realized that with that Zumbani that we have been told by others it causes loss of appetite (laughing).

CM: It increased or?

RES: There was nothing that I was able to eat, he told me that I would die of hunger, so he told me what I used to tell him when he was sick you don’t eat the food for its satisfaction.

CM: Hmm

RES: You must eat so that you get fit and have energy. He encouraged me then I ate that porridge

CM: Hmm

RES: The other thing that made me happy was that no one was infected because I was afraid that the children might be infected because they are 12 and 17years so I was fearing that these children might get sick, but nothing happened

CM: Nothing happened

RES: Hmm

CM: That is great, I would like to ask you, you said you had depression at that time what about anxiety or stress?

RES: Yeah, it was there because I felt scared, the reason I was afraid was when we were tested, I had spent the whole day here with people who started to test positive I spent the day mingling with them

CM: The whole day?

RES: Yeah, we were doing statistics seated on the same table after spending the whole day mingling that’s when someone came to my inbox and said sister get tested because so and so has tested positive for COVID-19 that’s what I did.

CM: Hmm

RES: After I heard it. I want to appreciate that I just decided then I asked sister charge where I could get tested.

CM: Hmm

RES: I didn’t even draw back from getting tested and I came from home prepared that whatever result I would accept it.

CM: Hmm

RES: But the fear was worse, I was thinking if I fail to breathe and we heard that oxygens were being bought, they are not easily found, then I said so what am I going to do but I want to thank God, that it wasn’t severe

CM: Okay you please tell me about your portfolio of work, what work do you do at the clinic?

RES: When I am here?

CM: Hmm

RES: My main activities are…. I am a midwife, I do ANC bookings, I treat pregnant mothers and children from birth up to 6 weeks, I do deliveries in the labor ward, and I manage clients in the post-natal ward.

CM: All right

RES: I can say that 75% of the time I will be doing deliveries in the labor ward.

CM: Okay are there any current reassignments that have changed on work maybe from the COVID-19 era, is there anything that has changed reassignment that has been added on your work due to COVID-19?

RES: Yeah, we reduced the number of visitors that come to see clients, now we are saying that our clients even those who are accompanied by family members we no longer letting in everyone at this gate.

CM: Hmm

RES: If you have looked at the time, you arrived here you saw that the clients were standing outside the gate.

CM: Yes

RES: We screen them there, those who are accompanied by family members we assess the client then we see if she can walk on her own the family member remains at the gate whilst the client gets in.

CM: Hmm

RES: Then for one patient we now want one visitor on top of that we are reinforcing the issue of masking and washing their hands and sanitizing.

CM: Hmm

RES: On our side, we had to advocate for PPE because initially, it wasn’t there even the mask they were difficult to get.

CM: Hmm

RES: But now they have managed to give us, we can say it’s better because we have PPE work suits, at least if you are doing delivery, you will have 95 and it is better, but things like gumboots we don’t have.

CM: I heard you saying that at first, you didn’t have so how were you working if you didn’t have PPE work suits how were you working, you even said even the masks were a problem what we’re doing those days?

RES: That time they could say if you have a mask that you have been given you keep it

CM: Then use it for how many days?

RES: For example that time you would use it for day and night duty

CM: Day and night?

RES: Yes, or you can find some if you can

CM: Personal

RES: Yes

CM: to buy the mask from your account or buy it for yourself?

RES: Yes

CM: Then you stay with them?

RES: Yeah

CM: But now how many are you getting per

RES: Now we can say at least it's better because if you are in the labor ward, we are now able to get these surgical 3 to 4 per day then the N95 at least 2

CM: Okay

RES: When you are doing delivery to the clients when they are in pain especially since she is in the second stage for them to breathe, she must take this out, so some remove and due to the threshold of the pain at times you can’t hear each other, some will be passing, out saliva and all that.

CM: Hmm

RES: So, we make sure when it is delivery you have N95 and plastic apron and shield, that if you want goggles, you can wear

CM: Okay

RES: Because initially some of the clients could even come without a mask. Sometimes you would give them, from those that you have been given then you give her to wear because you can’t leave her like that.

CM: Okay I would want you to explain to me how the health situation is in the environment that you work in, what we call the health context that you work in how it is, are there concerns that are there about the situation of how you are working right now in COVID-19?

RES: I think the issue of time our work must be reduced so that will limit our time interacting with the patients.

CM: How many hours are you working right now?

RES: We work 8 hours per day

CM: 8 hours per day?

RES: Hmm

CM: What about those on the night shift how many do they do?

RES: Almost 10 to 12 hours ah 12 hours because it's 7 to 7

CM: 7 pm to 7 am?

RES: Yes

CM: What about those who have come for the afternoon shift what time do they finish?

RES: They finish at 7

CM: 7 in the next morning after they have started at 1?

RES: No to those who come in the afternoon shift they finish at 7 pm

CM: Okay the finish at 7 pm?

RES: Yes

CM: Oh it’s okay

RES: But due to the issue of transport problems we are trying to reduce the hours and the days of coming here if you come like……It’s a local arrangement. If you come as you start at 1 pm finish the day shift then enter into night duty

CM: Okay

RES: Then you go home one time

CM: Ooh you be having off on other days?

RES: Yes

CM: Like today if you start at 12 then finish at 7 then I enter the night shift then finish at 7 the next day there is no need for transport or going out again

RES: Yes

CM: Okay I see, so other concerns that are there besides working hours that are too much during the pandemic, what are others?

RES: I think incentive even food because it’s like if we look at this COVID-19 it suppresses a person’s immunity, diet maybe if our employer could provide diet food that if we come to work, we can eat, but you bring a lunchbox from home. We were thinking that we would be provided with a high protein diet the likes of milk, bread, and margarine but there’s nothing or even fruits but there’s nothing.

CM: Ooh okay what measures or changes are you making in response to COVID-19 as an individual, what have changed as an individual in response to the pandemic?

RES: We have minimized movements I told myself that if I came back from work, I was supposed to stay at my house and not interact with many people.

CM: Hmm

RES: Even the children even if a person knocks on the gate, I go there wearing a mask, even if I go where I go I practice social distancing, masking sanitizing, and all

CM: What about as an organization what changes took place here at the clinic in response to COVID-19?

RES: We can say the changes that took place are when people arrived at the gate we did temperature checks, those with high temperature or with symptoms if you saw the blue tent when you entered you entered the gate we put them there, we have testers that are there we notify those people

CM: Hmm

RES: Then they go and get tested then when we are taking their temperatures and entering, they wash their hands and we sanitize them and we are limiting the numbers we no longer allow them to enter in large numbers

CM: Hmm

RES: For example, at ANC we have a wide catchment area so the maximum number that we are looking for new bookings is up to 10 we work with those 10.

CM: Per day?

RES: Yes

CM: Previously how many were you booking per day?

RES: 40-50

CM: Per day?

RES: Yeah

CM: So how are you selecting those 10 per day?

RES: We say the first 10

CM: Okay first come first serve?

RES: Yes, we sometimes relax if a person is sick, we take her.

CM: Okay how do you cope with these measures or changes in your line of work, how are you seeing these changes that took place how are you coping, with the changes that happened?

RES: Initially wearing a mask was difficult saying I struggle with breathing but after seeing that you will get sick and die this is normal, we have realized that you must stay with a mask and it’s no longer a problem.

CM: Hmm

RES: Because initially during the first lockdown if you were in a mask people would look at you in wonder what you were doing, but now this is normal if you don’t have a mask people will look at you in wonder so in a way we have overcome.

CM: Okay from your perspective how are health workers perceiving the situation, your colleagues and other health workers how are they seeing the situation of COVID-19?

RES: If we look now everyone has accepted and they are seeing the reality and now everyone knows that COVID-19 is real. And worse with our situation here we know that the clinic once closed when others tested positive, and others got sick at home, and we encouraged each other to wake up and steam every morning.

CM: Hmm

RES: Everyone is practicing, and following all the regulations though here and there some are forgetting.

CM: Okay I would want to know which month was it when you closed for 2 weeks?

RES: February

CM: This last February?

RES: Ahh no it was the end of January

CM: Entering in February

RES: Yes

CM: Now we want to talk about experiences and how COVID-19 impacted PMTCT services. How has the coronavirus impacted the delivery of PMTCT services?

RES: Some of our clients were missed for example with the cases that the clinic has closed, and that person didn’t have anywhere to go to they delivered at home.

CM: Hmm

RES: Some babies missed Nevirapine the first 72 hours of their life, and some of the conditions they were giving birth in at home were dirty environments.

CM: Hmm

RES: We are seeing a number of babies that missed nevirapine dose, in the first 72 hours, or babies who were never tested for HIV and some of the conditions they were giving birth in at home were dirty environments and you see babies with all sorts of infections

CM: Okay how are you handling that situation

RES: We are testing them first we if they are positive pit them on treatment. If they are not we put them on cotrimoxazole till they are weaned off

CM: Is there anything that was affected, or did you feel that there were glitches somewhere in the whole PMTCT cascade or the whole PMTCT program looking at testing, commodities, and medicine are there any bottlenecks that have happened?

RES: Yes, it might have happened during the COVID-19 peak because of the issue that some mothers are late bookers so when they gave birth at home. Some were not tested, and some were positive and those precautions for preventing the baby were not taken and that is a disaster.

CM: Hmm

RES: And some of them we would see them maybe after they got sick or at 6 weeks or after they if they are passing by the clinic then you test her you find out that she is positive, but she has a new baby, and the baby has not received any Nevirapine at birth

CM: Okay what about in terms of retention in the care of both new and old patients, did you maintain the old patients and those who were new?

RES: Eh some partially defaulted because when they came to the clinic maybe it was closed at that time.

CM: Hmm

RES: Then due to lockdowns at times traveling was difficult a person will need a letter and at times on roadblocks, you know that it was difficult

CM: Hmm

RES: And those boys (Police officers and soldiers) who we will be at roadblocks were saying that we don’t read books we can’t read, so we were encouraging them that try to use a health center that is near to them.

CM: Okay

RES: Then there was a time when a mobile team was moving around delivering ARVs for patients.

CM: Where was this mobile team coming from was it specifically for this facility or it was done for every facility?

RES: It was moving throughout Harare I don’t know where it was from but remember that there was one.

CM: Were there people from this community who were served with that mobile clinic of ARVs?

RES: Yes

CM: Okay what about issues of sample transportation do you send your samples outside for testing?

RES: At times but most of the time they are done here we have a lab here.

CM: Okay there was no glitch or bottleneck in terms of sample transfer?

RES: Yes

CM: What about the ability of health care workers to come to work were there any bottlenecks or challenges that happened, I heard you saying due to transport challenges a person could do double shifts, adding shifts from 12pm to 7 pm from 7pm to 7am so that they could get transport in the morning?

RES: Initially during the first lockdown we were provided with transport because many people who work here come from Damofalls, Zimre Park, and Ruwa so we use lifts because no transport is straight be it the Kombis or Zupco that comes here.

CM: Hmm

RES: We were provided with transport, but it had a natural death we do not know what happened.

CM: Okay without any notice?

RES: No

CM: Without anything any communication?

RES: No

CM: Okay

RES: So, it led to staff shortages as some could not come to work and those that managed to come would be overwhelmed because the clients would want to be helped and sometimes traveling was difficult. I still remember there was one time we were walking because there were no lifts, one day I was waiting for a lift to work, and I was told by the police to go back home.

CM: Hmm

RES: People were beaten and were instructed to stay indoors. I went back home then I called the sister in charge and told her that’s what happened and was not able to report for duty.

CM: Okay was there any fear of getting infected with COVID-19 you mentioned fear a lot is there anything else that you want to share?

RES: There was fear that I could be infected, and I realized that I had to protect myself.

CM: Okay how about the issue of lack of PPE materials you have said something but now how is it, you said that in the first days, it was difficult even the mask was a problem you said that you don’t have gumboots at the moment, but what has improved from the time ,it started?

RES: Ehh now we can say it better at least we are now able to be given masks every day when we come to work, the sanitizers are there

CM: You said you are given how many?

RES: We are given these surgical masks 3-4 then N95 2, then we are given each one pair of goggles, then we have face shields we have protective suits, the gumboots are not there, then we have plastic aprons and theatre caps.

CM: Okay everyone if you arrive at work that is a pack for each day?

RES: Yes

CM: But back then you didn’t have all these things?

RES: Even soap to wash our hands we couldn’t find it, it wasn’t there

CM: I would like to know how long you worked without…when you worked without enough PPE?

RES: I can say we took a long time

CM: Can you estimate how many months or how many weeks if you can

RES: It was almost 3-4 months because we did an industrial action after we did that industrial action, and we were not paid because they were saying we were not reporting for duty.

CM: Okay

RES: We ended up coming back after seeing that at times you are seated at home there’s nothing that is working for you.

CM: Hmm

RES: And we ended up coming back, then there was an inspection that was done they said it must be written as a report as proof because the main thing that we had were just scrub suits, they added scrub suits but the challenge that is still there is the issue of gumboots we don’t have.

CM: So, what are you working with right now if you don’t have gumboots?

RES: We wear our shoes

CM: They are the ones you come in with here and go out with going back home

RES: No, we have personal crocks that stay here in our lockers, and we use them when we are at work, we do not take them home.

CM: Okay how does the coronavirus situation compare to your experiences of other disease outbreaks like cholera and typhoid, we want to compare your experience to coronavirus how is it?

RES: Ahh this one was deadly looking at typhoid and cholera that were infecting people who were poor and suffering, those were the people we know that do stay in dirty locations because they are poor, they can’t buy resources.

CM: Hmm

RES: But if we look at Coronavirus it infected rich people. I think because people with money are the ones who do anything and they eat junk food, they are the ones who can go out and have parties, they are the ones who are able to interact with many people as compared to poor people.

CM: Hmm

RES: We just eat dirty vegetables sitting at home, but these ones have a lot of interactions plus if we look at cholera it is happening in third-world countries where we are suffering.

CM: Hmm

RES: But if you look at Coronavirus it came from Europe where they are wealthy compared to down here.

CM: When you were talking, you said it came to the rich because they are the ones that do everything what are you trying to say these are the ones who eat what is it called?

RES: Junk foods

CM: Yes, Junk foods but before you mentioned junk food you said they are the ones who do anything…………...

RES: They do parties, they do clubs we can say their social interaction is too much

CM: Okay can you explain to me the changes that were there in services provision as a response to COVID-19, you have talked about other things you were seeing the patients outside then they were tested you have been found with high temperatures they were referred to the blue tent that is outside, you minimized your clients and visitors, what else what other changes did you do in response to the coronavirus pandemic?

RES: Ehh we tried to see our clients according to priority, meaning that we will be attending to the very ill quickly and we budget our time, we tried to reduce time so that people must not stay for long at the clinic.

CM: Okay

RES: A person must arrive get help and go back home.

CM: Hmm

RES: And we must not crowd people.

CM: Okay when you are saying being helped are you saying one delivers today you go back home today?

RES: Yes, especially like post-natal mothers now we are giving an allowance of at least 6 hours then we say go home.

CM: Okay

RES: Then like clients who will be coming to ANC for repeat visits we are now booking them with time because before the coronavirus we would tell them to come at 11 but now we are booking them in a diary we say the first 5 arrive at 11, the second 5 we say at half 11 or quarter to 12.

CM: Hmm

RES: Giving allowance that we would be done with these 5 as they go out the other 5 will be coming in.

CM: Okay how are you booking the 5 are you booking them over the phone, or someone must come to the clinic at register her name and they come later to do the booking how are you doing it?

RES: When they come the first time, we give them date for repeat visit we will then tell her that you come on this date at maybe 11am and they must stick to that time so that the workflow cannot be disturbed

CM: Okay

RES: We have a diary that when you are doing ANC you would check to see how many clients are coming today, but half of the time some of the clients they don’t follow the just come to book any day and time.

CM: On their desired time?

RES: Hmm

CM: So, what do you do to them let’s say I was told 1 o’clock I arrived with the group at 11 o’clock what do you do?

RES: We assess if it’s not a busy day we just attend her but if its busy we then tell her come on you prescribed time or if we have enough staff coverage, we attend her.

CM: Okay do you think your patients had all the information required during the national lockdown?

RES: With our patients what I can say is every time you meet them you have to teach them, and we just must reemphasize to them every time.

CM: Okay did they know where to go for PMTCT services during the national lockdown?

RES: Some knew some didn’t.

CM: Those who did know what did they do?

RES: Those who knew came to the clinic in labor and collected their medications and for their babies and those who did not know you would see them maybe when the baby is at 3 weeks old, and they don’t have nevirapine and the baby was not tested.

CM: Hmm

RES: And maybe what has brought them to the clinic is that the baby is sick and then to others, you could see her and she says nurse I didn’t see you around I gave birth, and I went to Newlands clinic, or I went to so and so clinic then I collected my medications and for the baby.

CM: Hmm

RES: Some they could even buy the nevirapine

CM: Okay what about how to get to the hospital including the travel requirements during lockdown did they have the knowledge?

RES: Some they had some they didn’t

CM: Okay those who had how were they doing it?

RES: For example, the previous caesarian cases could call an ambulance and go to Mbuya Nehanda, or they used their private cars, or they could even hire taxis.

CM: Okay did they know how to handle themselves at the facilities, when they were here did, they know what they were supposed to do during these lockdowns and COVID-19 when they arrived at the clinic?

RES: It remains a challenge up to now some come without any masks, some don’t even wash their hands, and some don’t even sanitize so we just continue educating them.

CM: Okay have you noticed any changes in the number of patients seeking care since the onset of the COVID-19 pandemic?

RES: There was a time that time when it was on pick.

CM: You’re talking about the December, January

RES: Yes, many people were ill so we would get many patients but as it has gone down.

CM: So you’re saying the December January time you had so many patients who were coming to seek care since the onset

RES: Yes

CM: What about back then remember our first lockdown was on January 20…. no sorry on 1 April 2020, when we had the first lockdown the one from the 1^st^ of April which kept on being extended how were the numbers?

RES: They were not much

CM: Okay December and January that’s when there was a pick how about now?

RES: Now it’s better they are few

CM: Okay your numbers have gone down?

RES: Yeah

CM: Now I want to talk about social issues at home maybe of the mothers in your opinion how do you think the following factors affected the women's access and utilization of PMTCT services, the issues of HIV status disclosure how did they affect their access and utilization of the PMTCT services?

RES: On the issue of disclosure to some the fact that both husband and wife will be at home and to others you know that they aren’t open to each other that’s what they are, they will be doing their thing in secret because they will be afraid just to say.

CM: Hmm

RES: With the issue of the lockdown for her to come will not be feasible …there was one case. I asked her why she had not come to collect the supply because she came 2 weeks later. She said sister let me you the truth….

CM: Hmm

RES: My husband is very violent; I didn’t even talk about it (status). For now, we are using condoms because I’m telling him that we should use them so that our baby will not be underweight.

CM: Hmm

RES: I didn’t tell him so for me to come here was difficult plus every day he would be home, so I just kept quiet. Then I said today you have come she said he was not home he had gone to work. They will be doing hide-and-seek games at home.

CM: Okay how about the issues of childcare roles and responsibilities

RES: Ehh

CM: Childcare roles and responsibilities during the era of COVID-19 how did they affect the women’s access and utilization of PMTCT services all the children are at home did it affect them in any way?

RES: It was a challenge to those who have children who cannot help and do things for themselves so they will stay home taking care of the children, then still on the children it was a challenge again because looking at the girl child many of them got pregnant.

CM: Hmm

RES: Eh I can say there are many teen pregnancies because the mother has left home to come here, and the child will be left doing whatever they want because they don’t have anything to do.

CM: What about access to control of resources, the resources that are at home their access to the control of resources did it affect women’s access and utilization of the PMTCT services, how did they affect them?

RES: Yes, it affected because other people who were not at work and some of the companies were not paying because you are not working, they were saying that there’s nothing we are doing so there’s no money to pay you

CM: Hmm

RES: If a mother needs to get a lift to come and collect pills and she doesn’t have money, half the time she would just stay at home because she doesn’t have money to come here.

CM: Okay about the issue of decision-making powers at both household and community level how did it affect?

RES: It affected them because for example if we look when it was said there is a mobile clinic that is coming to the communities and if the community sees you standing on that car…….

CM: So, can you explain more about the mobile clinics how they work and give drugs, how did they move how did they communicate with the clients?

RES: It was a social group I didn’t know which organization was it, but it was put on clinics that notice board and groups …. They had put dates that on these dates we are in Warren Park on these dates we are in Kuwadzana on these dates we are in Mabvuku. There was one client that told me that she given with the mobile

CM: Hmmm

RES: Then I asked her where she got them then she said they arrived in our area this one stays in Eastview and that’s where she went and collected her medication. Then I asked her how she knew then she said there’s a health promoter she’s the one who told me that they are giving pills.

CM: Okay health promoter from their community?

RES: Yes, I didn’t know where the health promoter got the information

CM: Okay

RES: But it had stigma because if you are seen standing on the mobile clinic car it would create problems.

CM: The government of Zimbabwe made major social changes that they implemented including social isolation, travel restrictions, closure of schools, and closure of borders what impact do you think they might have on women in your catchment of Mabvuku?

RES: It had a negative impact on that some of them are cross borders they will be crossing borders going to South Africa that’s their means of survival that’s the job they do for the family to survive, then the borders have closed there’s is nowhere….to others, they were closed whilst they were still there, and the children were left suffering here alone.

CM: Hmm

RES: To others, they had the children here but didn’t have anything to give them that’s why other children resorted to doing whatever they did, and they ended up having teenage pregnancies plus to these children, they were seated at home and what was being said of online teaching it’s not all of them that can afford

CM: Hmm

RES: Because they couldn’t get food, but I heard that there are donors who are helping children I heard there is PLAN International

CM: In which areas?

RES: In Mabvuku Tafara, Eastview, Ruwa and Epworth

CM: They are helping with what?

RES: They give people voucher cards that are written social support it is said that what they do if they arrive home, they assess your diet what you eat then they see if there is anyone who goes to work, how you survive mainly they will be focusing on diet.

CM: Hmm

RES: Then if they see that you can qualify, they give 12 USD per month per individual in the household

CM: Okay

RES: So that voucher card they will tell them that go and buy to the shop, they are some selected shops that they are supposed to buy from.

CM: Hmm

RES: And usually they emphasized to buy high protein diet so there are some selected shops that they are allowed to go and buy and the days they are supposed to go and buy

CM: Okay do you think these measures are feasible, especially in your community or catchment area that we have talked about that has been social isolation, travel restrictions closure of borders, and closure of schools were these feasible if we look at your community?

RES: People were just up and down especially here in Mabvuku you’ve seen at the shops they are people who were at the front of Choppies. They have moved and started selling across the road and some they were not even masking they were walking like that.

CM: Do you mean measures were not feasible at all?

RES: Partly

CM: Partly?

RES: Hmm

CM: Which ones do you say were, feasible which ones do you say were not feasible?

RES: We can say others understood that they must practice social distancing, they must wear masks and if you don’t have a letter, you must not travel, you must stay at home because if you stayed at home, you would reduce your interaction with people thereby reducing your exposure

CM: Hmm

RES: But to others, they were saying it doesn’t matter

CM: What measures or programs are needed to mitigate these negative impacts that have been brought by COVID-19 in this community? The measures that you think need to be done or programs that need to be done to mitigate the impacts in your area.

RES: I think looking especially to children who got pregnant they need support groups

CM: Support groups?

RES: Yes

CM: What else is needed?

RES: They also should be encouraged to continue going to school

CM: Hmm okay

RES: Then if possible because if we look at our country many people are not working, maybe if more jobs can be created, I don’t know how, so that at least people can be able to survive.

CM: Hmm

RES: Because what made people breach the COVID-19 rules and regulations is that they did not have food they would not just sit whilst their families suffer. The rent was needed so those are some of the things that made them breach. Maybe sometimes if we say everyone must be formally employed it’s not possible.

CM: What do you think are some of the health impacts of COVID-19 including beyond the infection itself.

RES: The…

CM: The impact of COVID-19 is beyond the infection right, if a person has been infected there was more impact people could see that a person has been infected but beyond the infection what other impact has been done by COVID-19?

RES: Stigma and discrimination because what was happening is that people would know that for example if we are in our area I still remember there was one time, I stay in Ruwa so there’s a Ruwa local board, so from the finance department they were tested and they were positive

CM: Hmm

RES: Then their houses were fumigated as I was coming from work you know the roads in location There were a lot of people standing in the road watching they said they were spraying because they have COVID. I just went and visited them.

CM: Hmm

RES: I went to see them; I went there wearing my mask the husband who had been tested was seated at the corner of the house and they were watching their TV then I talked to the wife that this would pass it’s just a phase then I just encouraged.

CM: Hmm

RES: I didn’t know the encouragement that I gave them went a long way, they later said that you are the only one who was able to come to our house and others were passing through just staring and…failing to say even a good morning. It lessened their love for the community.

CM: Hmm

RES: It chased away love yes if a person is infected, we must show each other love and at the same time you must be protecting yourself and the one who’s infected also protecting him/herself

CM: Hmm

RES: And to others, it brought even neglect that a person couldn’t find someone to help because you can imagine that you are sick, you can’t breathe you are alone you can’t cope you need someone to help you

CM: Hmm okay what do you think are the socio-economic impacts of COVID-19 short-term socio-economic impacts and long-term?

RES: Yeah economic challenge because the economy went down and if we look at some people who have started doing their business they were destroyed even if we look at for example those of Kombis (Minibusses) look at that there are other families it was their job and the kombis were banned in the roads.

CM: Hmm

RES: So is loss of employment and the other issue is that the children are not going to school the children are seated at home they have nothing to do, some children resort to drugs, and some become pregnant.

CM: Hmm

RES: There was domestic violence you spent the whole day seated at home looking at each other and there was no mealie meal and even vegetables, unintended look at how many mothers got pregnant there was no recreation (laughing) you were spending the whole day looking at each other and you end up doing the deed.

CM: What do you recommend should be done as a national response to the COVID-19 pandemic?

RES: I think as a response to this pandemic especially when they do lockdowns I think people should be given…our government should look into it they must give people food assistance because people were dying of hunger which made them breach the COVID-19 rules.

CM: Hmm

RES: People need food

CM: Hmm what else besides food assistance?

RES: Then helping them with rentals.

CM: Okay what measures or programs need to be put in place to help mitigate the negative impacts of the COVID-19 pandemic within the workplace and the community, what should we do, so that we help each other to mitigate or to remove the negative impacts of COVID-19 at our workplaces and in the community

RES: Ongoing education should continue disseminating the information and telling people that COVID-19 is real and it’s not the end of it they should also practice the measures of masking, social distancing, sanitizing

CM: It’s okay thank you that’s the end of our discussion do you have any questions or anything that you want to add on

RES: I can say if we have a relative who has encountered that problem we just have to give love, we should help each other

CM: It’s okay thank you so much we have finished our interview

RES: Thank you
